# Essential Roles of the Smc5/6 Complex in Replication through Natural Pausing Sites and Endogenous DNA Damage Tolerance

**DOI:** 10.1016/j.molcel.2015.10.023

**Published:** 2015-12-17

**Authors:** Demis Menolfi, Axel Delamarre, Armelle Lengronne, Philippe Pasero, Dana Branzei

**Affiliations:** 1 IFOM, the FIRC Institute of Molecular Oncology, Via Adamello 16, 20139, Milan, Italy; 2 IGH, Institute of Human Genetics CNRS UPR 1142, 141 rue de la Cardonille F-34396 Cedex 5, Montpellier, France

## Abstract

The essential functions of the conserved Smc5/6 complex remain elusive. To uncover its roles in genome maintenance, we established *Saccharomyces cerevisiae* cell-cycle-regulated alleles that enable restriction of Smc5/6 components to S or G2/M. Unexpectedly, the essential functions of Smc5/6 segregated fully and selectively to G2/M. Genetic screens that became possible with generated alleles identified processes that crucially rely on Smc5/6 specifically in G2/M: metabolism of DNA recombination structures triggered by endogenous replication stress, and replication through natural pausing sites located in late-replicating regions. In the first process, Smc5/6 modulates remodeling of recombination intermediates, cooperating with dissolution activities. In the second, Smc5/6 prevents chromosome fragility and toxic recombination instigated by prolonged pausing and the fork protection complex, Tof1-Csm3. Our results thus dissect Smc5/6 essential roles and reveal that combined defects in DNA damage tolerance and pausing site-replication cause recombination-mediated DNA lesions, which we propose to drive developmental and cancer-prone disorders.

## Highlights

• Cell-cycle-regulated alleles reveal Smc5/6-essential functions to segregate in G2/M
• Genetic screen with new hypomorphic allele identifies processes relying on Smc5/6
• Smc5/6 acts on recombination structures triggered by endogenous replication stress
• Smc5/6 prevents fragility and mediates replication through natural pausing sites

## Graphical Abstract

**Figure:**
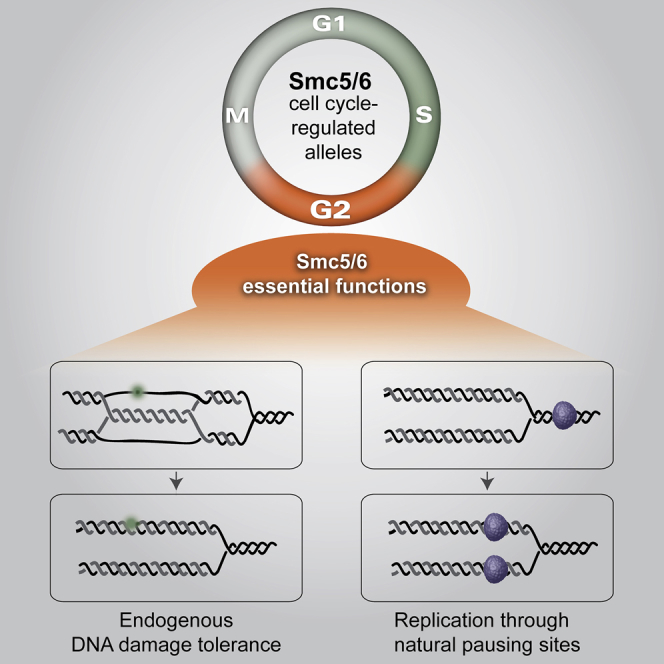

## Teaser

Menolfi et al. explore the roles of the Smc5/6 complex in proliferation. Cell-cycle-regulated alleles reveal Smc5/6 essential functions to segregate in G2/M. Genetic screens with generated alleles underpin roles for Smc5/6 in the metabolism of recombination structures triggered by endogenous replication stress, and in replication through natural pausing sites.

## Introduction

Genomic instability, a hallmark of cancer cells, is induced by DNA damage and replication stress. DNA damage response (DDR) mechanisms act as crucial barriers against genetic instability and are mediated by conserved pathways that ensure DNA damage recognition and repair (Branzei and Foiani, 2008).

The Structural Maintenance of Chromosomes (SMC) complex Smc5/6, related to cohesin and condensin, contributes to DNA repair (Lehmann, 2005). Smc5/6 also facilitates organization and segregation of repeat elements and binds to topological intermediates generated during replication (Jeppsson et al., 2014b, Murray and Carr, 2008). However, to what extent these functions contribute to its important/essential roles in cellular proliferation remains unknown. Notably, Smc5/6 contains a SUMO ligase, Mms21/Nse2, which is important for the described functions of the complex (Bermúdez-López et al., 2015, Branzei et al., 2006, Zhao and Blobel, 2005), and was recently identified to be mutated in a developmental disorder characterized by primordial dwarfism (Payne et al., 2014).

As an SMC complex, Smc5/6 is bound to exert its chromosome metabolism functions while being associated with chromatin (Jeppsson et al., 2014b). In this regard, previous studies outlined distinct binding patterns for Smc5/6 on chromosomes during and after DNA replication (Lindroos et al., 2006). In S phase, Smc5/6 associates with origins of replication (Bustard et al., 2012), whereas in G2/M, Smc5/6 binds to centromeres and various chromosome arm locations (Lindroos et al., 2006) in a manner dependent on sister chromatid cohesion and correlating with the presence of topological intermediates known as precatenanes or sister chromatid intertwinings (Jeppsson et al., 2014a, Kegel et al., 2011). However, the roles performed by Smc5/6 when bound to such chromosome addresses are not well understood.

Here we set out to investigate Smc5/6 roles in proliferation. Using *Saccharomyces cerevisiae* cell cycle-regulated alleles that enable restriction of Smc5/6 subunits to S or G2/M, we identified that Smc5/6 essential functions are manifested selectively in G2/M. Employing genome-wide genetic screens made possible by newly generated *smc5/6* alleles, we further uncovered pathways that compensate for Smc5/6 deficiency in G2/M. Our results reveal crucial roles for Smc5/6 in processing DNA structures formed in the context of endogenous error-free DNA damage tolerance (DDT) and in facilitating replication through natural pausing sites predisposed to fragility (Song et al., 2014, Tourrière and Pasero, 2007). The findings also reveal important interplay between Smc5/6 and other genome caretakers in chromosome metabolism processes that are crucial for proliferation and genome integrity.

## Results

### Smc5/6 Restriction to G2/M Allows Functional Complex Assembly and Chromosome Association

**Figure 1:**
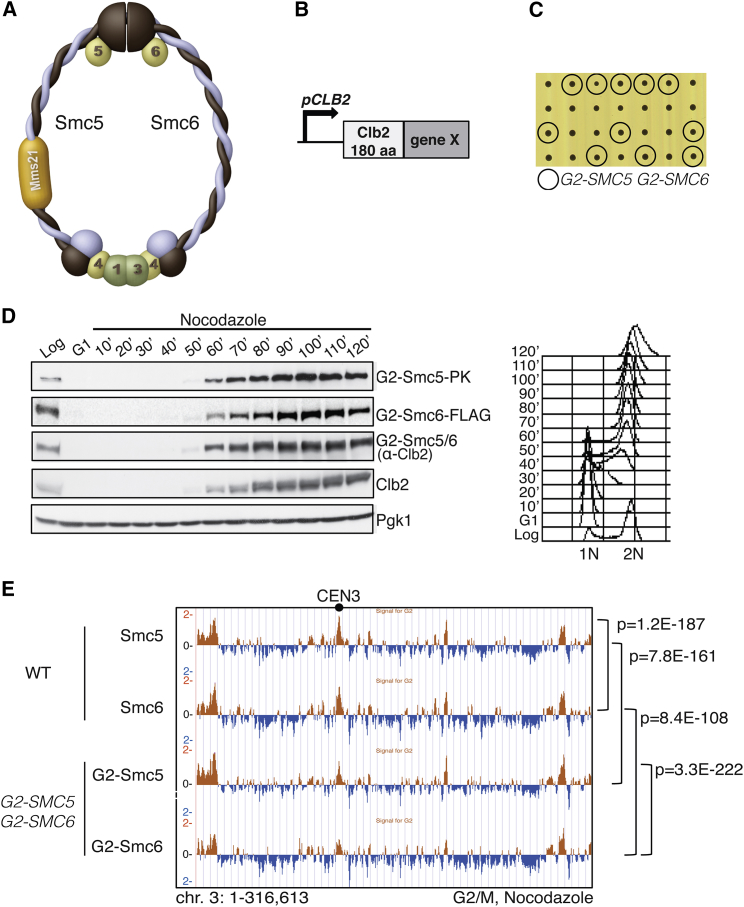
Smc5/6 Restriction to G2/M Allows Normal Proliferation and Smc5/6 Chromatin Localization in Metaphase Cells (A and B) Schematic representation of the octameric Smc5/6 complex (A) and of the Clb2-derived G2-tag (B). (C) *G2-SMC5 G2-SMC6* cells, derived from crosses between *G2-SMC5* and *G2-SMC6*, are viable. (D) *G2-SMC5* and *G2-SMC6* expression is coincident with the one of *CLB2* and restricted to G2/M. Pgk1 serves as loading control. (E) ChIP-on-chip profile of Smc5-PK, Smc6-FLAG, and G2-Smc5-PK, G2-Smc6-FLAG from G2/M-synchronized WT and *G2-SMC5 G2-SMC6* cells, respectively. Chromosome 3 is shown as example. The indicated p values relate to the genome-wide overlap between the considered ChIP-on-chip protein clusters. See also Figure S1.

To address Smc5/6 S phase functions, we limited to G2/M the expression of individual genes encoding for six components of the octameric Smc5/6, namely *SMC5*, *SMC6*, *NSE1* (encoding a putative ubiquitin ligase), *MMS21/NSE2* (encoding for a SUMO ligase), *NSE4* (encoding the subunit that connects the globular heads of Smc5 and Smc6), and *NSE5* (encoding one of the two subunits associated with the hinge domains of Smc5/6) (Figure 1A). To these ends, we swapped the individual promoters of the above-mentioned genes with a modified form of the mitotic cyclin Clb2 promoter and the N-terminal part of Clb2 that includes its degrons (Figure 1B), referred to as the G2-tag (Karras and Jentsch, 2010). To avoid difficulties in scoring lethality if Smc5/6 functions were essential during DNA replication, we constructed diploid cells heterozygous for the allele under study and recovered haploid cells with the desired G2-allele by tetrad dissection (see Figure S1A available online, and see below). Notably, the G2-tag did not interfere with cell viability and fitness for any of the six Smc5/6 components analyzed, although the restriction to G2/M was in all cases successful (Figure S1B and data not shown).

Smc5/6 associates with replication forks stalled by hydroxyurea (HU) (Bustard et al., 2012). To examine if Smc5 still binds replication forks when its SMC partner, Smc6, is restricted to G2/M, we analyzed Smc5 chromatin clusters using genome-wide chromatin immunoprecipitation (ChIP) studies (ChIP-on-chip) in HU-treated wild-type (WT) and *G2-SMC6* cells. We found that Smc5 binds in the proximity of replication forks (Figure S1C), well in line with previous reports (Bustard et al., 2012), but it does not show the bimodal distribution around replication origins characteristic of other SMC proteins, such as cohesin and Rad50 (Tittel-Elmer et al., 2012). Differently from WT, we observed almost no binding to chromatin for Smc5 in *G2-SMC6* cells (Figure S1C). Importantly, in the same strains, Smc5 chromatin clusters in G2/M were qualitatively identical in WT and *G2-SMC6* cells, indicating that Smc5/6 complexes containing the G2-Smc6 protein can efficiently assemble postreplicatively (Figure S1D).

To examine a context in which the existence of Smc5/6-like complexes consisting of Smc5 or Smc6 homodimers can be fully excluded early in S phase, we constructed a double mutant *G2-SMC5 G2-SMC6*. Notably, these cells were also characterized by normal growth (Figure 1C), although the G2-Smc5/6 variants were absent in S phase (Figure 1D). Moreover, G2/M-restricted Smc5/6 variants were correctly degraded in anaphase as predicted by the G2-tag (Figure S1E). Importantly, G2-Smc5-PK and G2-Smc6-FLAG chromatin clusters showed genome-wide statistically significant overlap to Smc5-PK and Smc6-FLAG from the corresponding WT cells in G2/M (Figure 1E). These observations demonstrate that G2-Smc5 and G2-Smc6 proteins can be assembled in a functional Smc5/6 complex in G2. Together, our observations indicate that the roles of Smc5/6 in S phase do not measurably impact on proliferation and are not essential for chromatin positioning and functions of Smc5/6 in G2/M.

### G2/M Restriction of Smc5/6 Allows Normal Replication Initiation and Replication Fork Speed

**Figure 2:**
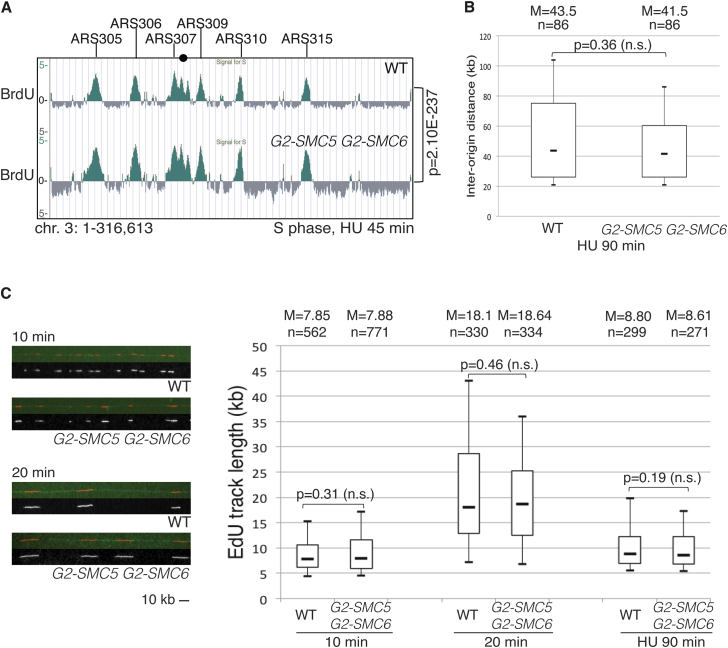
Smc5/6 Restriction to G2/M Allows Normal Origin Firing and Replication Fork Speed (A) BrdU IP-on-chip profile in WT and *G2-SMC5 G2-SMC6* cells synchronously released in media containing HU and BrdU. Chromosome 3 is shown with annotated early *ARS* regions. The p value of the genome-wide overlap between the BrdU clusters in the two strains is indicated. (B) WT and *G2-SMC5 G2-SMC6* were synchronized in G1 and released in S phase in the presence of EdU and HU. Box plot analysis of the interorigin distance (IOD) in the two samples. For each strain, the median (M) value and the number (n) of the IODs analyzed are indicated. The p value, calculated with the Mann-Whitney test, indicates nonsignificant differences between WT and *G2-SMC5 G2-SMC6*. (C) Asynchronous WT and *G2-SMC5 G2-SMC6* cells were treated with EdU, and cells were collected at 10 and 20 min afterward for molecular combing analysis. Alternatively, WT and *G2-SMC5 G2-SMC6* were synchronized in G1 and released in S phase in the presence of EdU and HU for 90 min. Examples of EdU tracks at 10 min and 20 min are reported. The box plot indicates the statistical analysis of the EdU-track length as in (B). See also Figure S2.

We further addressed if absence of Smc5/6 during S phase affects the replication program and replication fork speed. Genome-wide BrdU profiles revealed that the origin firing pattern and the firing efficiency were qualitatively identical in WT and *G2-SMC5 G2-SMC6* cells replicating in the presence of HU (Figure 2A). Consistent with this result, the interorigin distance was comparable in the two strains, as calculated by molecular combing (Figure 2B). We next examined the replication fork speed of individual forks. Both in unperturbed and HU conditions, the length of the replicated tracts was highly similar between WT and *G2-SMC5 G2-SMC6* cells (Figure 2C and data not shown). Thus, S phase-specific conditional depletion of Smc5/6 does not alter the origin-firing program and fork speed.

Smc5/6 promotes tolerance of replication-associated lesions, such as those induced by methyl-methanesulfonate, MMS (Branzei et al., 2006). We found that the replicated tract lengths were similar in WT and *G2-SMC5 G2-SMC6* cells replicating in the presence of MMS (Figure S2), indicating that Smc5/6 role in MMS tolerance is largely postreplicative. We conclude that Smc5/6 can fully provide for chromosome integrity functions both in unperturbed and DNA damaging conditions if supplied in G2/M.

### Smc5/6 Essential Functions Are Manifested in G2/M

**Figure 3:**
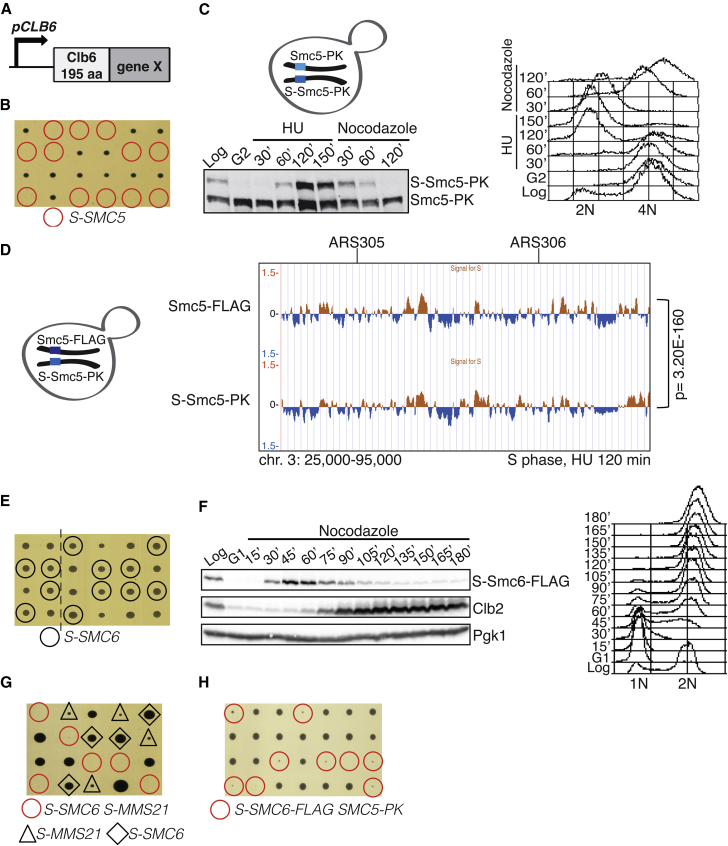
Restriction of Smc5/6 to S Phase Has Adverse Effects on Proliferation (A) Schematic representation of the Clb6-derived S-tag. (B) *S-SMC5* cells derived from sporulation of *SMC5/S-SMC5* heterozygous diploids are not viable. (C) Expression of *SMC5-PK* versus *S-SMC5-PK*. Asynchronous *SMC5/S-SMC5* diploid cells were arrested in G2/M, released in media containing HU for 150 min, and then released from HU in media containing nocodazole for 120 min. Western anti-PK and FACS are shown. (D) ChIP-on-chip profile of Smc5-FLAG and S-Smc5-PK from diploid *SMC5-FLAG/S-SMC5-PK* cells released from G2/M arrest in media containing HU for 120 min. A snapshot of chromosome 3 is reported; p value of genome-wide overlap of clusters is indicated. (E) *S-SMC6* cells are viable. The line indicates elimination of superfluous lanes from the tetrad dissection plate image. (F) *S-SMC6-FLAG* expression was monitored by western blotting using G1 cells released in media containing nocodazole. Clb2 and Pgk1 (loading control) blots are also shown. (G and H) *S-SMC6 S-MMS21* and *S-SMC6-FLAG SMC5-PK* obtained by tetrad dissection are not viable. See also Figure S3.

Next, we analyzed the consequences of restricting the expression of Smc5/6 components to S phase, using an analogous S-tag (Figure 3A) (Hombauer et al., 2011). The S-tag utilizes the promoter and the N terminus degron elements of the S phase cyclin, Clb6, to induce expression of the modified gene early in S phase and cause degradation of the encoded protein at the end of S phase (Jackson et al., 2006). While heterozygous *S-SMC5/SMC5* cells were viable, haploid *S-SMC5* cells were not (Figure 3B). To examine if this lethality is due to impairments in *S-SMC5* expression, we analyzed the two Smc5 variants, Smc5 and S-Smc5, both C-terminally tagged with PK, in the heterozygous diploid. For this purpose, we synchronized the *S-SMC5/SMC5* diploids in G2/M and released the cells in the presence of HU and then in media containing nocodazole (Figure 3C). As expected for an S-tagged variant, S-Smc5 was detected in S phase diploids at levels comparable with WT Smc5 and declined as cells reached G2/M (Figure 3C). Next, we analyzed if S-Smc5 was still functional in S phase for binding stalled replication forks. To simultaneously analyze Smc5 and S-Smc5 binding profiles, we tagged the two *SMC5* alleles of the heterozygous diploid with different C-terminal tags. ChIP-on-chip analysis of Smc5-FLAG and S-Smc5-PK chromatin clusters in diploid cells traversing S phase revealed strong similarity and statistically significant chromatin cluster overlap (Figure 3D). Thus, successful restriction of *SMC5* expression to S phase causes lethality.

Next, we applied the S-tag to other five components of Smc5/6. We observed variation in the severity of the phenotypes, commensurate with the efficiency and timeliness of the S-tagged proteins degradation (see below). Notably, four out of the six S-tagged alleles caused either lethality, as in the case of *SMC5* (Figure 3B) and *NSE4* (Figures S3A and S3B), or severe slow growth, as in the case of *MMS21* and *NSE1* (Figures S3C–S3D and data not shown). In contrast, *S-SMC6* and *S-NSE5* haploid cells were viable (Figure 3E and data not shown). Time course analysis of S-Smc6 revealed that it was correctly produced in S phase and began to be degraded when cells started to express *CLB2*, but that low levels of S-Smc6 still persisted in G2/M (Figure 3F). Moreover, both S-Smc6 and Smc6 were proficient in binding to early origins of replication and showed qualitatively identical ChIP-on-chip profiles in HU (Figure S3E), indicating that S-Smc6 is functional in S phase. The results demonstrate that the low levels of S-Smc6 persisting in G2/M are sufficient for viability and suggest that *S-SMC6* is potentially a hypomorphic allele, selectively defective in G2/M due to low amounts of Smc6, and can be used in genetic screens to uncover essential functions of Smc5/6 complex manifested in G2/M (see below).

While the persistent low amount of Smc6 in G2/M can explain the apparent incongruence in cell viability obtained by limiting *SMC5* and *SMC6* expressions to S phase, this contention further predicts that a more severe impairment of Smc5/6 function in G2/M would be detrimental to growth. Indeed, combination of *S-SMC6* and *S-MMS21* alleles resulted in lethality (Figure 3G). Moreover, when we attempted to examine if the low levels of Smc6 present in *S-SMC6* cells (Figure 3F) allow chromatin recruitment of Smc5/6 in G2/M—which we planned to address by ChIP-on-chip analysis of Smc5 and Nse4 clusters—we found that combination of the *S-SMC6* allele with PK-tagged alleles of *SMC5* and *NSE4* resulted in very severe synthetic fitness defects (Figures 3H and S3F). These findings, together with the severe effects on viability caused by limiting *SMC5*, *NSE4*, *MMS21*, and *NSE1* expression to S phase, support the notion that Smc5/6 essential functions are manifested after bulk chromosome replication, in G2/M.

### Identification of Pathways Providing for Viability in Cells with Limited Levels of Smc5/6 in G2/M

**Figure 4:**
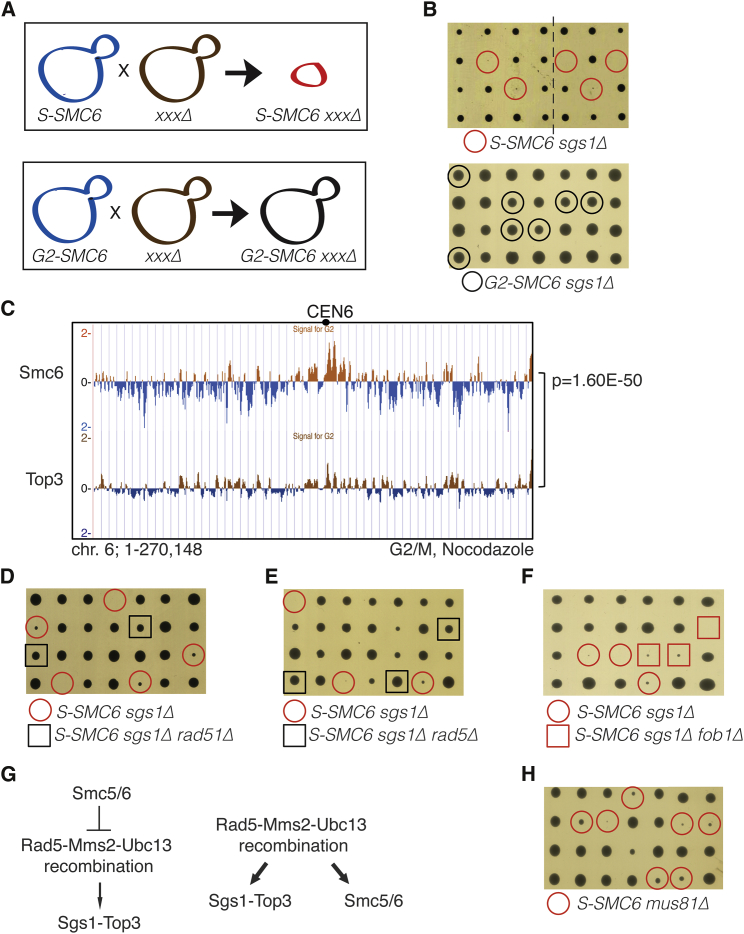
Functional Interaction between Smc5/6 and Sgs1-Top3-Rmi1 in G2/M (A) Synthetic genetic array screens conducted between *S-SMC6* and the yeast nonessential knockout library. (B) *S-SMC6*, but not *G2-SMC6*, is synthetic lethal with *sgs1Δ*. The line indicates elimination of superfluous lanes from the tetrad dissection plate image. (C) ChIP-on-chip profiles of Smc6-FLAG (the same as in Figure 1E) and Top3-FLAG from G2/M-arrested cells. Chromosome 6 is shown as example; p value of genome-wide overlap of the considered protein clusters is indicated. (D–F) Individual deletions of *RAD51* and *RAD5*, but not of *FOB1*, rescue *S-SMC6 sgs1Δ* lethality. (G) Two scenarios of Smc5/6 functions in relation to Rad5- and Rad51-dependent recombination structures formed in unperturbed conditions. (H) *S-SMC6* is synthetic lethal/sick with *mus81Δ*. See also Figure S4.

To identify pathways that act redundantly or in compensation with Smc5/6 in G2/M, we conducted robot-assisted synthetic genetic array screens with *S-SMC6* (Figure 4A). Three classes of mutations were repeatedly picked up by the screen, excluding those that appear nonselectively in other SGA screens conducted in the lab, petite mutants, and the *LGE1/BRE1* class required for S-phase cyclin gene expression (Zimmermann et al., 2011) and which likely causes lethality due to very low levels of S-Smc6. Of these, two groups comprised genes previously connected to DNA metabolism and were retained for validation and scoring for potential synthetic interactions also with *G2-SMC6*. In this way, we identified distinct pathways of chromosome integrity that are required for viability in conditions of limited amounts of Smc5/6 in G2/M. These pathways and the studies exposing their interrelation with Smc5/6 are described below.

### Smc5/6 Facilitates Resolution of Recombination Structures Formed during Endogenous DNA Damage Tolerance

A group of mutants displaying synthetic sickness/lethality with *S-SMC6*, but not with *G2-SMC6*, consisted of the Sgs1-Top3-Rmi1 (STR) complex (Figures 4B, S4A, and S4B). Moreover, we found that the genome-wide clusters of Smc5/6 and Top3 had statistically significant overlap (Figure 4C and data not shown), suggesting that Smc5/6 and Top3 may act in proximity and/or have common substrates. Both Sgs1 and Smc5/6 have been tightly linked to the processing of DNA damage tolerance (DDT) template switch intermediates induced by DNA damage (Branzei et al., 2006, Branzei et al., 2008, Choi et al., 2010, Sollier et al., 2009). Efficient formation of template switch intermediates depends on both Rad51 and Rad5/Mms2/Ubc13-mediated PCNA polyubiquitylation, a feature that distinguishes template switching from other recombination-dependent processes. Interestingly, we found that *rad51Δ*, as well as *rad5Δ*, *ubc13Δ*, and *mms2Δ* mutations, rescued the synthetic lethality of *S-SMC6 sgs1Δ* (Figures 4D, 4E, S4C, and S4D). Although both Smc5/6 and Sgs1 play important roles in regulating recombination at ribosomal DNA (rDNA) (Fricke and Brill, 2003, Torres-Rosell et al., 2005), we found that deletion of *FOB1*, a condition that largely suppresses rDNA recombination, did not rescue *S-SMC6 sgs1Δ* lethality (Figure 4F). Thus, the functional interaction between Smc5/6 and STR does not predominantly reflect rDNA-related events. In all, the above results expose a role for the Rad5 pathway in generating DNA substrates for STR and Smc5/6 following physiological levels of replication stress.

Two mutually nonexclusive scenarios can explain the Rad5-dependent genetic interactions observed between *S-SMC6* and *sgs1Δ* mutations. The first places Smc5/6 upstream and as counteracting the Rad5 pathway responsible for producing recombination substrates (Figure 4G, left panel). In the second, Smc5/6 facilitates resolution of Rad5-dependent structures, jointly or in parallel with STR (Figure 4G, right panel). Both scenarios entail an increase in recombination-mediated DDT structures in the absence of Sgs1 and Smc5/6 that would impair chromosome segregation if left unresolved (Matos et al., 2011, Szakal and Branzei, 2013). Judging from the ability of Smc5/6 to promote resolution of damage-induced recombination structures when activated in G2/M (Bermúdez-López et al., 2010), the observation that Rad5 mediates template switching in S phase (Karras et al., 2013), and that the G2/M but not the S phase Smc5/6 function is required for viability in *sgs1Δ* (Figure 4B), the model placing Smc5/6 action downstream of the Rad5 step in processing endogenous recombination DDT intermediates appears most probable (Figure 4G, right panel).

Additional evidence we obtained substantiates the above view. Specifically, we found that similarly to *sgs1Δ*, the *S-SMC6* mutation causes slow growth in *pol32Δ* cells (Figure S4E), mutated in the noncatalytic subunit of replicative polymerase δ and suffering of replication stress associated with activation of the error-free DDT branch (Karras and Jentsch, 2010). If the Rad5-dependent DNA structures requiring Smc5/6 during unperturbed proliferation are akin to those triggering template switching after DNA damage, inactivation of Smc6 in G2/M may cause an enhanced requirement for alternate resolution mediated by Mus81-Mms4 and by the Slx4 scaffold (Ashton et al., 2011, Gritenaite et al., 2014, Szakal and Branzei, 2013). Supporting this hypothesis, *S-SMC6* was slow-growing in combination with *mus81Δ*, *mms4Δ*, and *slx4Δ* (Figure 4H and data not shown). In all, the results provide evidence for endogenous Rad5 and PCNA polyubiquitylation-dependent DDT operating during unperturbed replication, and for a postreplicative role of Smc5/6 in recombination-mediated DDT activated by endogenous replication stress.

### Smc5/6 Functionally Cooperates with Rrm3 at Natural Pausing Sites

**Figure 5:**
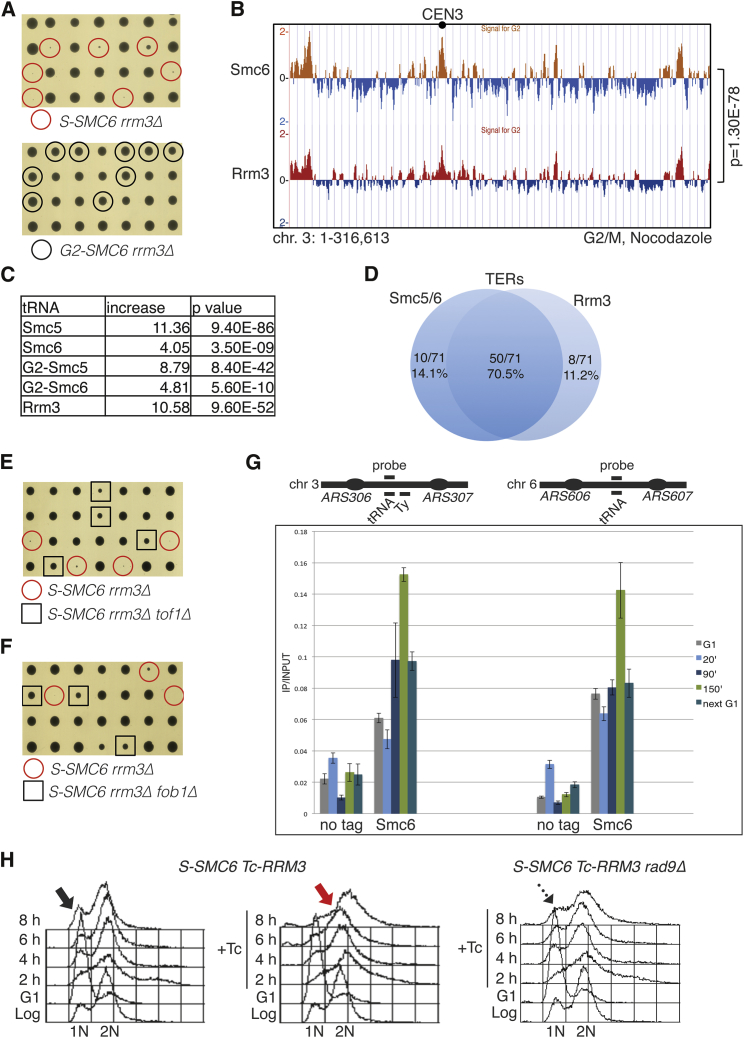
Smc5/6 and Rrm3 Are Enriched at Natural Pausing Sites and Counteract the Toxicity Associated with Tof1/Cms3- and Fob1-Mediated Pausing (A) *S-SMC6*, but not *G2-SMC6*, is synthetic lethal/sick with *rrm3Δ*. (B) ChIP-on-chip of Smc6-FLAG (the same as in Figure 1E) and Rrm3-FLAG from G2/M-synchronized cells. Chromosome 3 is shown and p value of the genome-wide overlap of clusters indicated. (C) Smc5, Smc6, G2-Smc5, G2-Smc6, and Rrm3 are significantly enriched at tRNA genes. The table reports the fold increase of each protein at tRNA genes, calculated versus the ones expected for random binding, and the p values of the significance. (D) Smc5, Smc6, and Rrm3 are enriched at pausing sites that serve as termination sites (TERs) in G2/M. Percentage of overlap and nonoverlap is shown. (E and F) Individual deletions of *TOF1* and *FOB1* rescue *S-SMC6 rrm3Δ* synthetic lethality. (G) ChIP-qPCR-monitored binding of Smc6-FLAG at pausing sites on chromosomes 3 and 6, also known as TER302 and TER603 (Fachinetti et al., 2010). The samples were collected in G1, at 20 min (early S phase), 90 min (late S phase/G2), and 150 min (G2/M) after release from G1 arrest in media containing nocodazole, and again in the following G1, after nocodazole removal and a second synchronization with α-factor. (H) *S-SMC6 Tc-RRM3* cells were synchronized in G1 and released in the absence or presence of tetracycline (Tc) for 8 hr. Samples for FACS analysis were collected every 2 hr. *S-SMC6 Tc-RRM3 rad9Δ* were analyzed in the presence of Tc. See also Figure S5 and Table S1.

Besides the STR mutant group, we identified that deletion of *RRM3* is sick/lethal in combination with *S-SMC6*, but not with *G2-SMC6* (Figure 5A). Rrm3 is a DNA helicase that facilitates fork passage through nonhistone protein-DNA complexes and through natural pausing sites, also referred to as replication fork barriers (RFBs) when they are located in the rDNA region (Azvolinsky et al., 2009, Ivessa et al., 2003, Mohanty et al., 2006). We found a statistically significant overlap between Smc5/6 and Rrm3 chromatin clusters in G2/M (Figure 5B). Moreover, similarly to Rrm3, Smc5/6 was enriched at tRNA and centromere (CEN) regions (Figures 5C and S5A), *HML* mating-type locus (Figure S5B), and pausing sites that serve as termination sites (TERs) during replication (Figure 5D; Table S1) (Fachinetti et al., 2010).

The conserved replisome-associated fork protection complex Tof1-Csm3 enforces pausing at pausing sites genome-wide (Calzada et al., 2005, Hodgson et al., 2007, Tourrière et al., 2005), and Fob1 strengthens RFB activity specifically at rDNA regions (Mohanty et al., 2006). Importantly, we found that the sickness/lethality of *S-SMC6 rrm3Δ* was rescued by individual deletions of *TOF1* and *CSM3* (Figures 5E and S5C), as well as by *FOB1* deletion (Figure 5F). This indicates that prolonged pausing and unfinished replication at rDNA (Torres-Rosell et al., 2007), which represents 8%–12% of the yeast genome, is the main contributor to the observed lethality.

Notably, however, we found Smc5/6 to be enriched at pausing sites situated outside the rDNA (Figures 5C, 5D, S5A, and S5B). Moreover, while the binding of Smc5/6 to RFBs located within rDNA was observed throughout the cell cycle (Figure S5D), we found that Smc5/6 binding to other natural pausing sites increased in late S-G2/M before declining in mitosis (Figure 5G), in parallel with an important role for Smc5/6 in *rrm3Δ* mutants manifested specifically after the end of bulk replication (Figure 5A). In addition, G2-restricted Smc5/6 variants were also enriched at natural pausing sites genome-wide (Figures 5C and S5A), indicating that this recruitment reflects a requirement for Smc5/6 at late stages of replication when most natural pausing sites are being replicated. Thus, Rrm3 and Smc5/6 colocalize to a large fraction of natural pausing sites and functionally complement each other.

To explore the molecular mechanisms underlying the growth defects in *S-SMC6 rrm3Δ* cells, we constructed conditional *S-SMC6 Tc-RRM3* cells. Inactivation of *RRM3* in *S-SMC6* by addition of tetracycline (Tc) caused prolonged G2/M arrest, as revealed by the prominent 2N DNA peak in the double mutant (Figure 5H). Deletion of genes with G2/M DNA damage checkpoint functions, such as *RAD9* and *DDC1*, but not of the spindle assembly checkpoint *MAD2*, largely alleviated the cell cycle defect of *S-SMC6 rrm3* conditional mutants (Figures 5H and S5E). As unfinished replication at rDNA does not trigger activation of the DNA damage checkpoint (Torres-Rosell et al., 2007), and Smc5/6 associates to pausing sites located outside rDNA, the results pinpoint a general role for Smc5/6 at natural pausing sites in preventing the formation of DNA lesions that can be sensed by the DNA damage checkpoint and cause mitotic delays.

### Smc5/6 Protects Natural Pausing Sites against Recombination-Mediated Fragility

**Figure 6:**
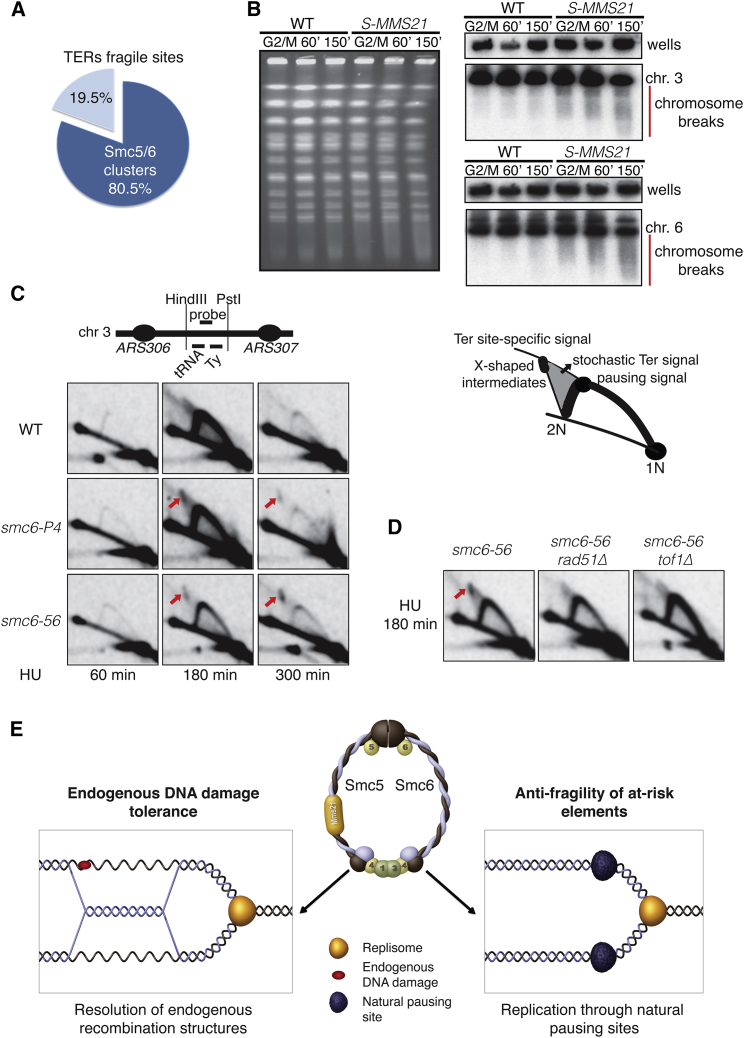
Smc5/6 Prevents Chromosome Fragility and Accumulation of Rad51- and Tof1-Dependent Recombination-like Structures at Natural Pausing Sites (A) Smc5/6 binds to 80.5% of the TERs identified as fragile sites in Song et al. (2014). (B) WT and *S-MMS21* cells were synchronized in G2/M and released in the following cell cycle in the presence of α-factor for 150 min. Samples for PFGE were collected at the indicated time points, and chromosomes were probed for regions containing natural pausing sites on chromosomes 3 and 6 represented in Figure 5G. The smearing in the gel highlighted by the red bar is representative of chromosome breakage. (C) WT, *smc6-P4*, and *smc6-56* were synchronously released from G1 arrest in S phase in the presence of HU at 30° for 2D gel analysis. Schematic representations of the 2D gel fragment analyzed and of the type of intermediates revealed by 2D gel electrophoresis. (D) *smc6-56*, *smc6-56 rad51Δ*, and *smc6-56 tof1Δ* were analyzed as in (C) at 180 min in HU following release from a G1 arrest. (E) Model summarizing the identified Smc5/6 roles that support proliferation. See also Figure S6.

While replication termination may not be generally associated with replication fork pausing (Dewar et al., 2015), a subset of natural pausing sites that serve as replication termination regions (TERs) (Fachinetti et al., 2010) were recently identified to represent hot spots of replication-associated chromosome fragility (Song et al., 2014). We found that 80% of the identified fragile TERs (Song et al., 2014) are enriched for Smc5/6 (Figure 6A; Table S1). Moreover, based on constitutively high levels of DNA damage in WT cells (Szilard et al., 2010), previous studies identified genomic loci equivalent of mammalian fragile sites in budding yeast. Interestingly, this set of fragile sites includes pausing sites such as tRNAs and mating type loci at which we found Smc5/6 to be enriched (Figures 5C and S5B).

To examine if limiting amounts of Smc5/6 in G2/M predispose to chromosome fragility, we analyzed chromosomes by pulse-field gel electrophoresis in *S-MMS21* cells (Figure S3C), more severely affected in the Smc5/6 function than *S-SMC6* (Figure 3E). Importantly, while we did not observe any major alterations in the chromosomes following staining of gels with ethidium bromide, Southern blot analysis revealed increased chromosome breakage, observed as smearing, in G2/M (Figure 6B and data not shown).

As replication-associated fragility is often associated with recombination-mediated genome rearrangements (Song et al., 2014), we addressed by 2D gel if *smc5/6* mutants previously characterized, *smc6-P4* and *smc6-56*, display an altered pattern of replication/recombination DNA structures at natural pausing sites while replicating at a permissive temperature. We chose a well-characterized region containing several DNA replication-pausing elements—tRNA, long terminal repeat (LTR) sequence, and Ty (Figure 6C)—and at which site-specific pausing, termination, and fragility were previously detected (Deshpande and Newlon, 1996, Fachinetti et al., 2010, Song et al., 2014). As chromosome breakage and recombination at pausing sites are relatively rare events (Calzada et al., 2005), but fragility is enhanced by slowing down of replication forks with various agents (Yunis et al., 1987), we added HU to increase the likelihood of detecting potential abnormalities. We used in vivo psoralen-crosslinking to prevent branch migration or other transitions during genome extraction that may obscure interpretation of the results. Replication started and proceeded with similar kinetics in all strains (Figure S6A), and replication structures and termination signals appeared efficiently (Figure 6C). Interestingly, both *smc6-P4* and *smc6-56*, but not WT cells, showed replication-associated accumulation of specific X-shaped signals (HU 3 hr) that persisted even when most cells have completed replication of the region (HU 5 hr) (Figure 6C). A similar accumulation of X-shaped intermediates was also visible in *S-MMS21* cells after 5 hr in HU (Figure S6B). Moreover, another analyzed pausing site, proximal to the centromeric region on chromosome 10 and at which we observed Smc5/6 to be enriched, showed an accumulation of X-shaped structures in *smc5/6* mutants at 5 hr in HU (Figure S6C). Importantly, the X-shaped structures, but not other replication intermediates, were dependent on Rad51 and Tof1 in both *smc6-56* and *smc6-P4* cells (Figure 6D and data not shown). To investigate genetically if these recombination events are toxic, we analyzed if, similarly to *TOF1* deletion (Figure 5E), *rad51Δ* also suppressed the lethality of *S-SMC6 rrm3Δ* cells. The triple mutants *S-SMC6 rrm3Δ rad51Δ* were viable but slow growing (Figure S6D), likely because of Rad51-dependent functions that contribute to viability in *smc5/6* mutants (Bustard et al., 2012).

Next, we analyzed if the detected recombination structures accumulating in the analyzed *smc5/6* mutants (Figures 6C, S6B, and S6C) are specific to pausing elements or are due to general stalling of replication forks caused by HU. For this purpose, we probed the genomic DNA to analyze the intermediates arising at an early efficient origin of replication, *ARS305*. Also at this locus, X-shaped structures that were not detected in WT cells accumulated in *smc5/6* mutants at late time points in HU (Figure S6E). Importantly, however, the X-shaped intermediates that formed in the proximity of origins (Figure S6E) were dependent on Rad51, but not on Tof1 (Figure S6F). Based on these results, we propose that Smc5/6 is recruited to natural pausing sites where it exerts an antifragility function by preventing prolonged Tof1-mediated fork pausing and associated toxic recombination events.

## Discussion

Differently from related SMC complexes, cohesin and condensin, the Smc5/6 functions implicated in proliferation remain hardly understood. Here, by generating cell-cycle-regulated *smc5/6* alleles that separate bulk replication functions from the ones performed later, we unveiled that the essential functions of Smc5/6 are manifested in G2/M. Using genome-wide genetic screens and selectively defective alleles, we underpinned two processes that crucially rely on Smc5/6 functions in G2/M: metabolism of endogenous DDT structures formed in response to replication stress and replication through natural pausing sites (Figure 6E). Interestingly, although both these processes relate to replication, our results indicate that the steps happening postreplicatively and at the very late stages during replication are particularly at risk and require the G2/M function of Smc5/6.

Recently, compound mutations in the *MMS21* component of Smc5/6 were shown to lead to primordial dwarfism (Payne et al., 2014), a condition observed in several replication disorders, such as Meier-Gorlin, caused by mutations in the prereplication complex (Bicknell et al., 2011). As previous work indicated that Smc5/6 associates with replicating chromatin and origins of replication (Bustard et al., 2012, Lindroos et al., 2006), a role for Smc5/6 in replication initiation or modulating fork speed would have provided a simple molecular rationale for the global growth impairments observed in *MMS21* mutated patients (Payne et al., 2014). Differently from this scenario, we found that Smc5/6 roles during S phase can be postponed to G2/M without overt perturbations in cell proliferation, replication program, or fork speed (Figures 1 and 2). Considering that origins of replication often behave as fragile sites (Di Rienzi et al., 2009), and our finding that Smc5/6 prevents fragility and recombination at natural pausing sites (Figures 6B and 6C), we propose that the binding of Smc5/6 to stalled replication forks in the proximity of origins is akin to its antifragility function at pausing sites (Figure 6E).

Growth impairments are often a common feature of DDR disorders caused by mutations in DNA repair factors (O’Driscoll et al., 2003), and defects in endogenous DDT were proposed to cause developmental problems (Klingseisen and Jackson, 2011). However, the DNA repair impairments associated with causal mutations in DDR syndromes are generally studied following treatments with ionizing radiation or various carcinogens that induce DNA damage and coincident activation of various stress response pathways. Here we provide evidence that Smc5/6 is required for DDT induced by endogenous replication stress associated with activation of Rad5/Ubc13/Mms2-mediated PCNA polyubiquitylation. In this context, Smc5/6 is crucial to compensate for deficiencies in the RecQ helicase Sgs1/BLM, mutated in cancer-prone Bloom syndrome patients, likely by its role in modulating resolution or remodeling of the emerging recombination intermediates (Figure 4G). When the postreplicative Smc5/6 levels are low, cells rely more on structure-specific endonucleases (Figure 4H) that can introduce crossovers and cause genome rearrangements (Gritenaite et al., 2014, Szakal and Branzei, 2013), indicating that low levels of Smc5/6 can predispose in the long run to genomic alterations characteristic of cancers. Interestingly, *SMC5* was recently identified as one of the genes with roles in brain metastasis development when mutated (Saunus et al., 2015). Our results indicate that this may reflect Smc5 ability to limit recombination events associated with genome landscape changes (Figure 6E). These roles of Smc5/6 may not be limited to regulating resolution of DDT recombination intermediates, but likely involve Smc5/6’s ability to prevent the formation/accumulation of toxic recombination structures at specific genomic elements that are predisposed to fragility or structural changes. Indeed, our present findings highlight that Smc5/6 is important for endogenous stress pathways that are not strictly linked to DDT. One such pathway we identified and characterized in this study is related to late-replicating zones containing natural pausing sites.

Common fragile sites (CFSs) and corresponding regions in yeast replicate very late and are prone to fragility (Cha and Kleckner, 2002, El Achkar et al., 2005, Song et al., 2014). Chromosome rearrangements at CFS drive tumor progression, but the role of these regions in normal cells is less understood. CFSs are under the crucial surveillance of the DNA damage-sensing kinase ATR (Mec1 in budding yeast) (Casper et al., 2002, Cha and Kleckner, 2002), mutated in Seckel syndrome and also characterized by primordial dwarfism (O’Driscoll et al., 2003). That the damage and replication checkpoint would monitor completion of replication at CFS is in apparent contrast with the proposed inability of the replication checkpoint to monitor incomplete replication at rDNA containing genetically programmed pausing sites (Torres-Rosell et al., 2007), and with the observation that Mec1/ATR is not required for the integrity of forks paused by RFBs (Calzada et al., 2005). However, based on our studies on Smc5/6 roles and differential recruitment at pausing sites located within rDNA and elsewhere, we propose that the two conclusions can be reconciled by considering that the chromatin environment and the nature of stalling at RFBs within rDNA are different from the ones of other natural pausing sites where single-stranded (ss) DNA formation and checkpoint activation may precede chromosome breakage (Feng et al., 2011).

Our study identifies Smc5/6 as an important factor in protecting the integrity of natural pausing sites. Besides constitutive roles in rDNA stability and at the numerous RFBs contained within the rDNA region, we find that Smc5/6 is recruited to a large fraction of known natural pausing sites late during replication (Figures 5C, 5D, and 5G). Deceleration in Smc5/6 function in G2/M increases genome fragility (Figure 6B), which further correlates with increased recombination events at natural pausing sites mediated by the replication fork protection complex, Tof1-Csm3 (Figures 6C and 6D). Importantly, the Tof1-dependent recombination events at natural pausing sites occur selectively in *smc6* mutants, but not in WT (Figures 6C and 6D). Because Tof1-Csm3 prevents fork rotation associated with precatenation at pausing sites (Schalbetter et al., 2015), it is possible that Tof1-restricted topological transitions may influence recombination and subsequent fragility at those regions. Importantly, our findings indicate that Smc5/6 is crucial in limiting those recombination events. We envisage that Smc5/6 promotes replication through natural pausing sites in one or several nonmutually exclusive ways. First, Smc5/6 may modulate fork reversal and/or processing of reversed forks (Xue et al., 2014) formed within topologically stressed regions. Second, Smc5/6 may facilitate resolution of topological constrains by promoting Top3 action on ssDNA regions contained within the positive supercoil in the unreplicated region ahead of the replication fork or on the precatenanes formed by fork rotation. In addition, Smc5/6 may stimulate resolution of the recombination intermediates induced by the topological transitions associated with prolonged pausing.

In conclusion, the present findings indicate Smc5/6 as a keystone regulator of genome integrity via its general roles in regulating DDT intermediate resolution genome-wide, as well as unique roles in modulating recombination at topologically constrained regions such as those happening during prolonged pausing (Figure 6E). We propose that coincident defects in these processes may be a common cause of developmental defects and contribute in the long run to the genome instability characteristic of cancers. Our results also provide leads for future work on understanding the interrelationship between endogenous stress response pathways and genome caretakers in chromosome maintenance processes that affect proliferation.

## Experimental Procedures

### Yeast Strains and Genetic Screens

The yeast strains used in this study are primarily derivatives of W303, and the relevant genotypes are shown in Table S2.

### Growing Conditions, Cell-Cycle Arrests, and Drug Treatments

Strains were grown at 25°C in YPDA medium unless otherwise indicated. G1 and G2 synchronizations were performed using 3–5 μg/ml of α-factor and 15–20 μg/ml of nocodazole, respectively. MMS was used at the concentration of 0.033%, HU at 200 mM, BrdU at 200 μg/ml, EdU at 50 μM, and Tetracycline at 600 μM.

### Protein Techniques, ChIP-qPCR, ChIP-on-Chip, and BrdU-IP-on-Chip

BrdU-IP, ChIP-on-chip experiments, and statistical analysis were performed as in Bermejo et al. (2009) and Fachinetti et al. (2010), and ChIP-qPCR as in Urulangodi et al. (2015).

### 2D Gels, FACS, PFGE, and Molecular Combing

2D gels and in vivo psoralen crosslinking were performed as in Giannattasio et al. (2014), FACS and pulse-field gel electrophoresis as in Branzei et al. (2006), and molecular combing experiments as in Bianco et al. (2012).

## Author Contributions

D.M. established the reagents; performed all experiments except those in Figures 2B, 2C, and S2; and analyzed the data. A.D., A.L., and P.P. performed and analyzed the combing experiments in Figures 2B, 2C, and S2. D.B. conceived and supervised the project, designed the experiments, analyzed the data, and wrote the paper.

## Accession Numbers

The microarray data are available online under the series number GSE72241 (http://www.ncbi.nlm.nih.gov/geo/query/acc.cgi?acc=GSE72241).

## Supplemental Information

Document S1. Figures S1–S6, Tables S1 and S2, and Supplemental Experimental Procedures

Document S2. Article plus Supplemental Information
