# Supplementary material for: Essential Roles of the Smc5/6 Complex in Replication through Natural Pausing Sites and Endogenous DNA Damage Tolerance
Source: Mol Cell. 2015 Dec 17;60(6):835–46. doi: 10.1016/j.molcel.2015.10.023 (PMC4691243; doi:10.1016/j.molcel.2015.10.023)
Supplement: Document S1. Figures S1–S6, Tables S1 and S2, and Supplemental Experimental Procedures [file mmc1.pdf]

Molecular Cell, Volume *60*

Supplemental Information

Essential Roles of the Smc5/6 Complex in Replication through Natural Pausing Sites and Endogenous DNA Damage Tolerance

Demis Menolfi, Axel Delamarre, Armelle Lengronne, Philippe Pasero, and Dana Branzei

SUPPLEMENTAL INFORMATION

Supplementary Figures and Legends

Figure S1, Related to Figure 1.

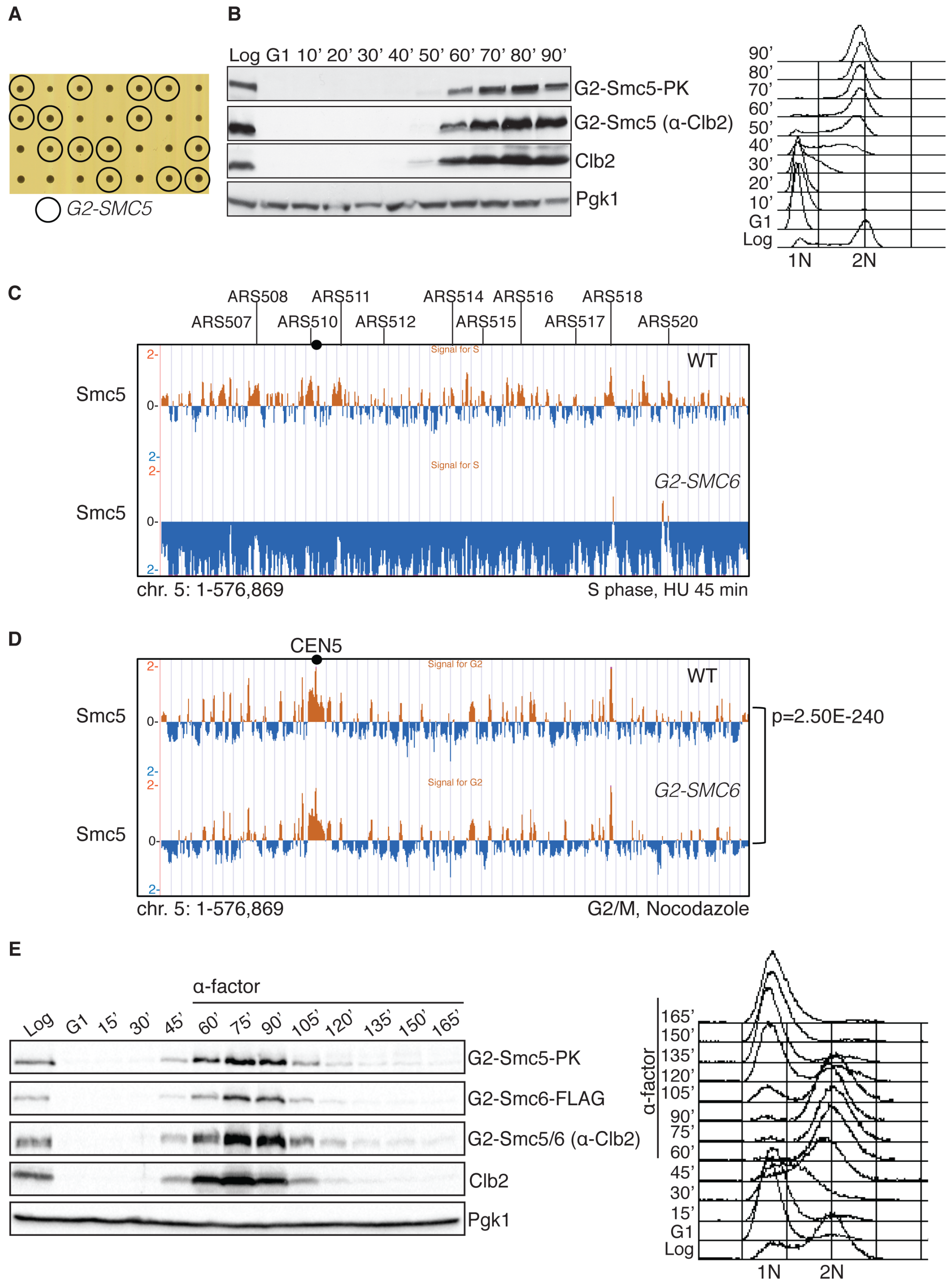

Figure S1. Restriction of individual Smc5/6 subunits to G2/M does not affect cell viability and chromatin association of the complex in G2/M. (A) Heterozygous *SMC5/G2-SMC5* cells were sporulated. *G2-SMC5* haploid cells, isolated by tetrad dissection, were characterized by normal fitness. (B) *G2-SMC5* expression occurred concurrently with the one of *CLB2* in G2/M and was not observed in S phase. FACS profile is also shown. (C) ChIP-on-chip profile of Smc5-PK in WT and *G2-SMC6* cells released from G1 arrest in media containing HU. Chromosome 5 is shown as example, with early origins of replication annotated. (D) ChIP-on-chip profile of Smc5-PK in WT and *G2-SMC6* cells in G2/M. Chromosome 5 is shown as example. The indicated p-value relates to the genome-wide overlap between the Smc5-PK clusters. (E) G2-Smc5/6 is correctly degraded in anaphase. *G2-SMC5 G2-SMC6* cells were synchronized in G1, released in normal medium, and after 60 min, α-factor was added again in order to arrest cells in the following G1. Samples for Western blot and FACS analysis were collected every 15 min.

Figure S2, Related to Figure 2.

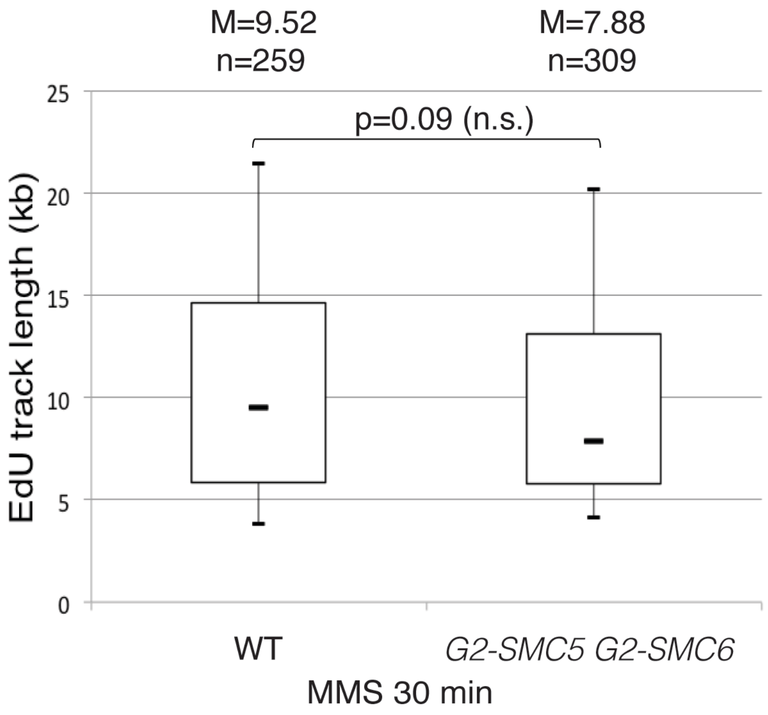

Figure S2. Restriction of Smc5/6 to G2/M does not affect fork progression following MMS treatment. WT and *G2-SMC5 G2-SMC6* cells were synchronized in G1 and released in the presence of EdU and MMS for 30 min, when samples were collected for molecular combing analysis. Box plot of the EdU-track length distribution. The median value of the length (M) and the number of the EdU tracks counted (n) are indicated for each strain. The p-value, calculated with a Mann-Whitney test, indicates that the difference observed between WT and the double mutant is not significant (n.s.).

Figure S3, Related to Figure 3.

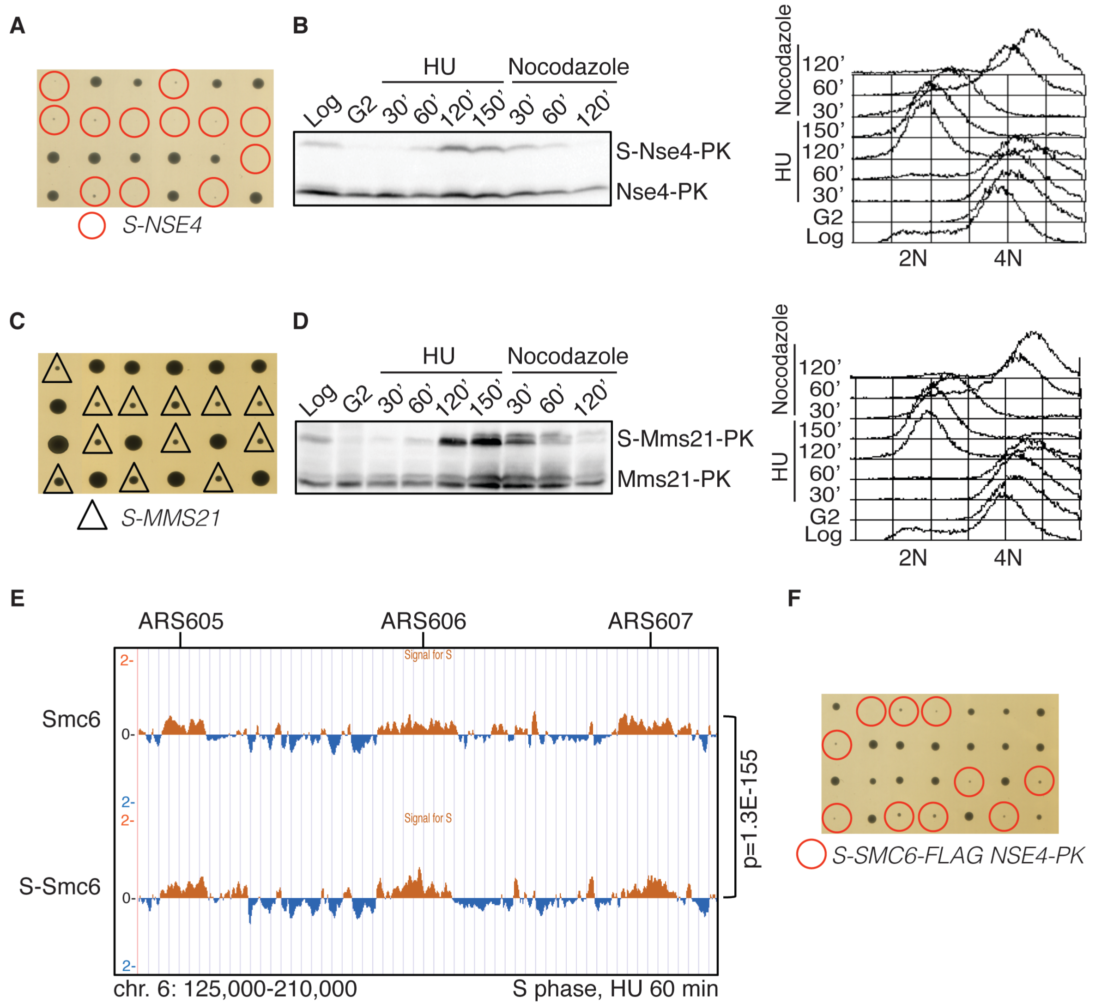

Figure S3. Restriction of Smc5/6 to S phase negatively impacts on cell viability. (A) Heterozygous *NSE4/S-NSE4* cells were sporulated and haploid cells were separated by tetrad dissection. Haploid cells corresponding to *S-NSE4* were not viable. (B) Expression of *NSE4-PK* versus *S-NSE4-PK* alleles in the heterozygous diploid. Asynchronous cells were arrested in G2 with nocodazole, released in media containing HU for 150 min, and then released in media with nocodazole for additional 120 min. Western blot anti-PK and FACS are shown. (C) *S-MMS21* cells obtained by tetrad dissection from heterozygous *MMS21/S-MMS21* cells are slow growing. (D) Expression of *MMS21-PK* versus *S-MMS21-PK* alleles in the heterozygous diploid as described in (B). (E) ChIP-on-chip of Smc6-Flag and S-Smc6-Flag from cells synchronously released from G1 arrest in the presence of HU for 60 min. A snapshot of chromosome 6 is shown with early *ARS* (*ARS605*, *ARS606*, *ARS607*) and genome-

wide p-value of the overlap between the considered protein clusters is indicated. (F) *S-SMC6-FLAG NSE4-PK* double mutants are not viable.

Figure S4, Related to Figure 4.

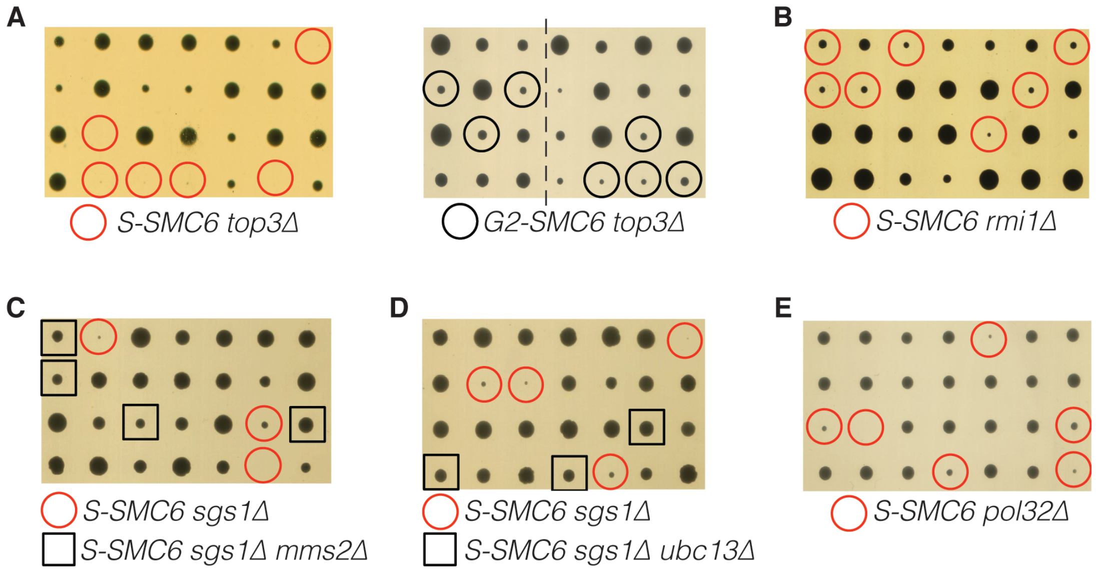

Figure S4. *S-SMC6* viability relies on multiple recombination intermediate resolvases. (A) *S-SMC6*, but not *G2-SMC6*, is synthetic lethal with *top3Δ*. The line indicates elimination of superfluous lanes from the tetrad dissection plate image. (B) *S-SMC6* is synthetic sick with *rmi1Δ*. (C, D) Individual deletions of *MMS2* and *UBC13* rescue the synthetic lethality of *S-SMC6 sgs1Δ*. (E) *S-SMC6* is synthetic lethal/sick with *pol32Δ*.

Figure S5, Related to Figure 5.

A

CEN	increase	p value
Smc5	24.39	6.10E-39
Smc6	17.99	5.80E-21
G2-Smc5	27.45	3.00E-40
G2-Smc6	18.52	2.40E-16
Rrm3	35.87	4.10E-65

B

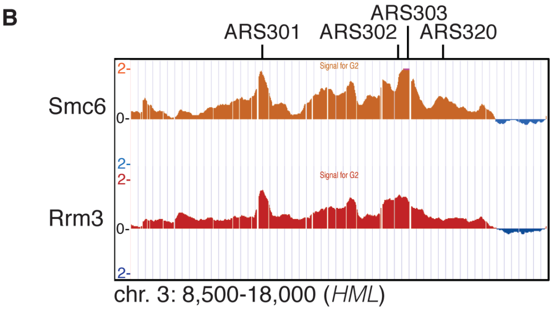

C

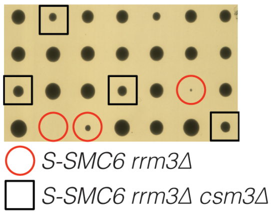

D

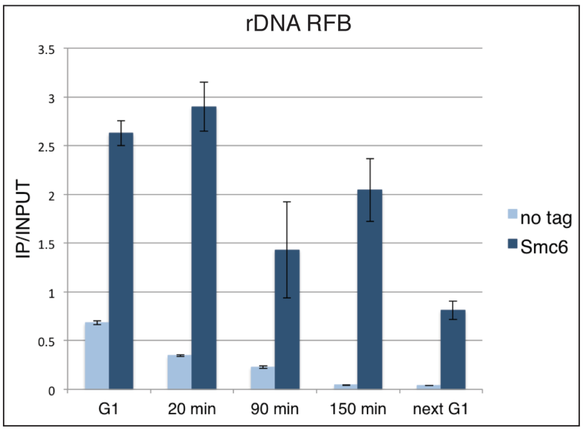

E

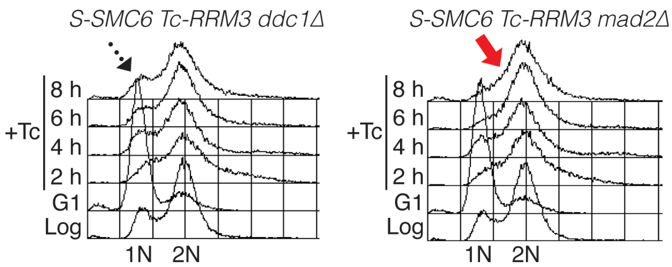

Figure S5. Smc5/6 localizes and functions at natural pausing elements. (A) Smc5, Smc6, G2-Smc5, G2-Smc6 and Rrm3 are significantly enriched at CENs. The table reports the fold increases of each protein at CENs, calculated versus the values corresponding to random binding, as well as the p-values of the enrichment significance. (B) Smc6 and Rrm3 are enriched at the mating type locus *HML*, known to be a pausing element in budding yeast (Wang et al., 2001). (C) Deletion of *CSM3* rescues the lethality of *S-SMC6 rrm3Δ*. (D) ChIP-qPCR-monitored binding affinity of Smc6-FLAG at rDNA RFB as in Figure 5G. (E) *S-SMC6 Tc-RRM3 ddc1Δ* and *S-SMC6 Tc-RRM3 mad2Δ* cells were synchronized in G1 and released in the presence

of tetracycline (Tc) for 8 hours. Samples for FACS analysis were collected every 2 hours as in Figure 5H.

Figure S6, Related to Figure 6.

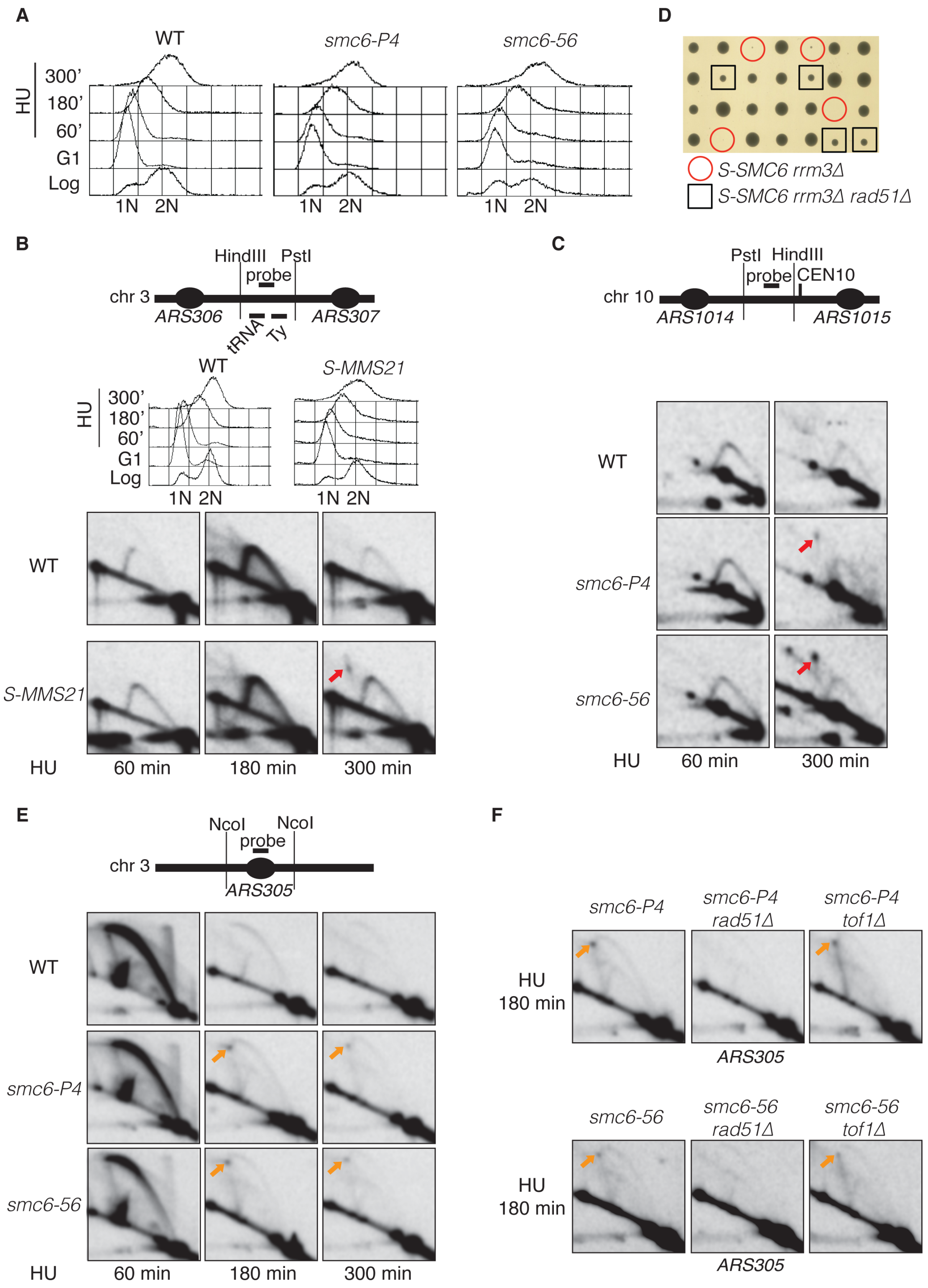

Figure S6. Different functions of Smc5/6 at early replication origins versus regions containing pausing elements. (A) FACS analysis of the 2D gel

electrophoresis experiment in Figure 6C, S6C and S6E (see below). (B) Genomic DNA from WT and *S-MMS21* cells was analyzed for replication intermediates at a pausing site on chromosome 3 as described in Figure 6C. (C) The 2D samples analyzed in Figure 6C were analyzed for another pausing site proximal to the centromere of chromosome 10. (D) Deletion of *RAD51* rescues the lethality of *S-SMC6 rrm3Δ*. (E) WT, *smc6-P4* and *smc6-56* were synchronized in G1 and released in HU at 30°C as described in Figure 6C. The same genomic DNA was digested in parallel with NcoI and analyzed for *ARS305*. (F) Genomic DNA samples isolated from strains of the indicated genotypes after release from G1 arrest in media containing HU were digested with NcoI and analyzed for *ARS305*.

Table S1, Related to Figure 5.

TERs (71)	Smc6-FLAG	Smc5-PK	Rrm3-FLAG
101	+	+	+
102	-	-	+
103	+	+	+
201	+	+	+
202	+	+	+
203	+	+	-
301	-	-	+
302	+	+	+
303	-	-	+
304	-	-	+
305	+	-	+
401	-	-	-
402	+	+	+
403	+	+	+
404	+	+	+
501	+	+	+
502	-	+	+
503	+	+	+
504	+	-	-
505	+	+	+
506	+	+	+
507	-	+	+
508	-	-	+
601	+	-	-
602	+	+	+
603	+	+	+
701	+	+	+
702	+	+	+
703	+	+	+
704	+	+	+
705	+	+	+
801	+	+	+
802	+	+	+
901	-	-	+
902	+	+	+
903	-	+	-
1001	+	+	+
1002	+	+	+
1003	+	+	+
1004	+	+	+
1005	+	+	+
1006	-	-	-
1101	+	+	-
1102	+	+	+
1103	+	+	+
1201	-	-	+
1202	+	+	+
1203	-	-	+
1204	+	+	+
1205	+	+	-
1301	+	+	+
1302	+	+	+
1303	+	+	+

1304	+	+	+
1401	-	+	-
1402	+	+	+
1403	+	+	+
1501	+	+	-
1502	+	+	+
1503	+	+	-
1504	+	+	+
1505	+	+	+
1506	-	-	-
1601	+	+	-
1602	+	+	+
1603	+	+	+
1604	+	+	+
1605	+	+	+
1606	+	+	+
1607	+	+	+
1608	+	+	+
	56	57	58
	79%	80%	81.70%

Table S1. Binding of Smc5/6 and Rrm3 to TER sites. The presence of significant clusters of binding for the protein analyzed by ChIP-on-chip is indicated by "+", lack of it with "-". The TERs are as defined in (Fachinetti et al., 2010) and the ones highlighted in blue were identified as fragile sites in (Song et al., 2014).

Table S2, Related to Figures 1-6. List of *Saccharomyces cerevisiae* strains used in this study.

Strain	Genotype	Source
**FY1363**	Mata *ade2-1 can1-100 his3-11,-15 leu2-3,112 trp1-1 ura3-1 RAD5+* (W303)	Lab collection
**FY0090**	Mata *his3-delta200 leu2-3, 112 lys2-801 trp1-1 (am) ura3-52* (DF5)	Lab collection
**HY1465**	W303 Mata *sgs1Δ::HIS3MX6*	Lab collection
**HY1473**	W303 Mata *mus81Δ::KANMX4*	Lab collection
**HY1545**	W303 Mata *sgs1Δ::HIS3MX6 rad5Δ::HPHMX4*	Lab collection
**HY1547**	W303 Mata *sgs1Δ::HIS3MX6 ubc13Δ::HPHMX4*	Lab collection
**HY1549**	W303 Mata *sgs1Δ::HIS3MX6 mms2Δ::HPHMX4*	Lab collection
**HY2721**	DF5 Matα *pol32Δ::klTRP1*	Lab collection
**HY2736**	W303 Mata *G2::NATNT2-SMC6*	This study
**HY2806**	W303 Mata *SMC6-6HIS-3FLAG::KANMX4*	This study
**HY3156**	W303 Mata *SMC5-9PK::HIS3MX6*	This study
**HY3158**	W303 Mata *G2::NATNT2-SMC6 SMC5-9PK::HIS3MX6*	This study
**HY3159**	W303 Mata *G2::NATNT2-SMC5-9PK::HIS3MX6*	This study
**HY3167**	W303 Mata *S::NATNT2-SMC6*	This study
**HY3168**	W303 Matα *S::NATNT2-SMC6*	This study
**HY3170**	W303 Mata *S::NATNT2-SMC6-6HIS-3FLAG::KANMX4*	This study
**HY3172**	W303 Mata *G2::NATNT2-SMC6-6HIS-3FLAG::KANMX4 G2::NATNT2-SMC5-9PK::HIS3MX6*	This study
**HY3386**	W303 Mata *S::NATNT2-SMC6-6HIS-3FLAG::KANMX4 rad51Δ::LEU2*	This study
**HY3447**	W303 Mata *SMC6-6HIS-3FLAG::KANMX4 SMC5-9PK::HIS3MX6*	This study
**HY3611**	W303 Mata *rmi1Δ::KANMX4*	Lab collection
**HY3807**	W303 Mata *TOP3-6HIS-3FLAG::KANMX4*	This study
**HY3840**	W303 Mata *ubc13Δ::HPHMX4*	Lab collection
**HY3841**	W303 Matα *mms2Δ::HPHMX4*	Lab collection
**HY3973**	W303 Matα *S::NATNT2-SMC6-6HIS-3FLAG::KANMX4*	This study
**HY4421**	W303 Mata *S::NATNT2-SMC6 tof1Δ::HIS3MX6*	This study
**HY4422**	W303 Mata *S::NATNT2-SMC6 csm3Δ::HPHMX4*	This study
**HY4425**	W303 Mata *S::NATNT2-SMC6 fob1Δ::HIS3MX6*	This study
**HY4896**	W303 Mata *ura3::URA3/GPD-TK(7X) G2::NATNT2-SMC6-6HIS-3FLAG::KANMX4 G2::NATNT2-SMC5-9PK-HIS3MX6*	This study
**HY4898**	W303 Mata *S::NATNT2-MMS21*	This study
**HY4904**	W303 Matα *S::NATNT2-SMC6 mms2Δ::HPHMX4*	This study
**HY4905**	W303 Mata *S::NATNT2-SMC6 ubc13Δ::HPHMX4*	This study
**HY4906**	W303 Mata/α *SMC5-9PK::HIS3MX6/S::NATNT2-SMC5-9PK::TRP1*	This study
**HY4909**	DF5 Mata *S::NATNT2-SMC6*	This study
**HY4915**	W303 Mata *S::NATNT2-SMC6 rad5Δ::HPHMX4*	This study
**HY4916**	W303 Mata *RRM3-10FLAG::KANMX4*	This study
**HY5163**	W303 Mata/α *MMS21-9PK::HIS3MX6/S::NATNT2-MMS21-9PK::TRP1*	This study

**HY5274**	W303 Mata *S::NATNT2-SMC6-6HIS-3FLAG::KANMX4 pAHD1-tc3-3HA-RRM3 (NAT)*	This study
**HY5276**	DF5 Mata *S::NATNT2-SMC6 pol32Δ::klTRP1*	This study
**HY5324**	W303 Mata/α *SMC5-6HIS-3FLAG::KANMX4/S::NATNT2-SMC5-9PK::TRP1*	This study
**HY5396**	W303 Mata *S::NATNT2-SMC6-6HIS-3FLAG::KANMX4 pAHD1-tc3-3HA-RRM3 (NAT) rad9Δ::HIS3MX6*	This study
**HY5398**	W303 Mata *S::NATNT2-SMC6-6HIS-3FLAG::KANMX4 pAHD1-tc3-3HA-RRM3 (NAT) ddc1Δ::HIS3MX6*	This study
**HY5400**	W303 Mata *S::NATNT2-SMC6-6HIS-3FLAG::KANMX4 pAHD1-tc3-3HA-RRM3 (NAT) mad2Δ:HPHMX4*	This study
**HY5433**	W303 Mata/α *NSE4-9PK::HIS3MX6/S::NATNT2-NSE4-9PK::TRP1*	This study
**HY5845**	W303 Mata *S::NATNT2-SMC6-6HIS-3FLAG::KANMX4 rad51Δ::LEU2 sgs1Δ::HIS3MX6*	This study
**HY5846**	W303 Mata *S::NATNT2-SMC6 rad5Δ::HPHMX4 sgs1Δ::HIS3MX6*	This study
**HY5847**	W303 Mata *S::NATNT2-SMC6 ubc13Δ::HPHMX4 sgs1Δ::HIS3MX6*	This study
**HY5848**	W303 Mata *S::NATNT2-SMC6 mms2Δ::HPHMX4 sgs1Δ::HIS3MX6*	This study
**HY5849**	W303 Mata *S::NATNT2-SMC6 mus81Δ::KANMX4*	This study
**HY5850**	W303 Mata *S::NATNT2-SMC6 rmi1Δ::KANMX4*	This study
**HY5851**	W303 Mata *G2::NATNT2-SMC6 top3Δ::KANMX4*	This study
**HY5853**	W303 Mata *S::NATNT2-SMC6 rrm3Δ::HIS3MX6 rad51Δ::LEU2*	This study
**HY5854**	W303 Mata *S::NATNT2-SMC6 rrm3Δ::HIS3MX6 tof1Δ::KANMX4*	This study
**HY5855**	W303 Mata *S::NATNT2-SMC6 rrm3Δ::HIS3MX6 csm3Δ::HPHMX4*	This study
**HY5856**	W303 Mata *S::NATNT2-SMC6 rrm3Δ::HIS3MX6 fob1Δ::HIS3MX6*	This study
**HY5861**	W303 Mata *G2::NATNT2-SMC6 sgs1Δ::HIS3MX6*	This study
**HY5862**	W303 Mata *G2::NATNT2-SMC6 rrm3Δ::HIS3MX6*	This study
**HY5890**	W303 Mata *smc6-56-13MYC::KANMX4 tof1Δ::HPHMX4*	This study
**HY5892**	W303 Mata *smc6-P4-13MYC::KANMX4 tof1Δ::HPHMX4*	This study
**HY5940**	W303 Mata *NSE4-9PK::HIS3MX6*	This study
**FY1002**	W303 Matα *rad51Δ::LEU2*	Lab collection
**FY1110**	W303 Mata *ura3::URA3/GPD-TK(7X)*	Foiani lab
**FY1267**	W303 Mata *smc6-56-13MYC::HIS3MX6 rad51Δ::LEU2*	Zhao lab
**FY1332**	W303 Mata *smc6-P4-13MYC::KANMX4*	Zhao lab
**FY1432**	W303 Mata *smc6-56-13MYC::KANMX4*	Zhao lab
**FY1535**	W303 Mata *smc6-P4-13MYC::KANMX4 rad51Δ::LEU2*	Zhao lab
**FY1765**	W303 Matα *rrm3Δ::HIS3MX6*	Foiani lab
**FY1766**	W303 Matα *top3Δ::KANMX4*	Foiani lab
**FY1856**	W303 Mata *ura3::URA3/GPD-TK(7X) [pRS415-hent1-LEU2]*	This study
**FY1857**	W303 Mata *ura3::URA3/GPD-TK(7X) G2::NATNT2-SMC6-6HIS-3FLAG::KANMX4 G2::NATNT2-SMC5-9PK-HIS3MX6 [pRS415-hent1-LEU2]*	This study

SUPPLEMENTAL EXPERIMENTAL PROCEDURES

Chromatin immunoprecipitation (ChIP)-on-chip and statistical analysis

Chromatin immunoprecipitation was carried out as previously described (Bermejo et al., 2009a; Bermejo et al., 2009b). Briefly, cells were collected at the indicated experimental conditions and crosslinked with 1% formaldehyde. Cells were washed twice with ice-cold TBS 1X, suspended in lysis buffer supplemented with 1 mM PMSF and 1X antiproteolytic cocktail (Complete protease inhibitor tablets, Roche) and lysed with FastPrep-24 (MP Biomedicals). Chromatin was shared to a size of 300-500 bp by sonication. IP reactions, with anti-FLAG (F1804, SIGMA) or anti-PK SV5-Pk1 (AbD Serotech) conjugated to Dynabeads Protein A (Invitrogen), were allowed to proceed overnight at 4°C. After washing and eluting the ChIP fractions from beads, crosslinks were reversed at 65°C overnight for both SUP and IP. After Proteinase K (Roche) treatment, DNA was extracted twice by phenol/chlorophorm/isoamylalcol. Following precipitation with ethanol and RNase A (SIGMA) treatment, DNA was purified using QIAquick PCR purification kit (QIAGEN). For ChIP-on-chip, DNA was amplified using WGA kit (SIGMA) following manufacturer's instructions. 4 µg of DNA from SUP and IP samples were hybridized to GeneChip *S. cerevisiae* Tiling 1.0R Array (Affimetrix) as described (Bermejo et al., 2009b). Evaluation of the significance of protein cluster distributions within the different genomic areas and protein-binding correlations was performed by confrontation to the model of the null hypothesis distribution generated by a Monte Carlo-like simulation as previously described (Bermejo et al., 2009a). The significance of the overlap between proteins clusters was evaluated as in (Bermejo et al., 2009a).

The microarray data are available online at the following link:
http://www.ncbi.nlm.nih.gov/geo/query/acc.cgi?acc=GSE72241.

ChIP-qPCR

ChIP-qPCR was performed using QuantiFast (SYBR Green PCR kit, QIAGEN) according to the manufacturer's recommendations and each real-time was performed at least in triplicate using a Roche LightCycler 480 system. The results were analyzed with absolute quantification/2nd derivative maximum (Roche LightCycler 480) and the 2(-

ΔC(t)) method as previously described (Livak and Schmittgen, 2001). Error bars represent standard deviations.

Antibodies

Monoclonal Anti-FLAG M2 (SIGMA, F1804), monoclonal anti-PK (AbD Serotech, SV5-Pk1), monoclonal anti-Pgk1 22C5 antibody (Invitrogen, A6457), polyclonal anti-Clb2 (Santa Cruz Biotechnology, y-180) were used. The BrdU-IP-on-chip analysis was carried out employing the anti-BrdU antibody MBL M1-11-3.

Pulse-Field Gel Electrophoresis (PFGE)

Genomic DNA samples were processed in low melting point agarose plugs (Branzei et al., 2006). For each time point analyzed agarose plugs containing 5 X 10^7 cells were incubated for 1 h at 37°C in 1 M sorbitol, 0.06 M EDTA, 0.1 M sodium citrate, 1 mg/ml Zymolyase and 0.2% β-mercaptoethanol. The plugs were then incubated in 0.5 M EDTA, 1% sarkosyl, 1mg/ml RNase A for 1 h at 37°C. Then, 2 mg/ml of Proteinase K was added and left overnight at 37°C. After extensive washing in buffer containing 10 mM Tris pH 7.5 and 50 mM EDTA, the agarose plugs were inserted in 0.9% agarose TBE 0.5X gel. Pulse-Field Gel Electrophoresis was performed for 10 h at 190V with 60s pulses, followed by 10h with 90 s pulses, in TBE 0.5X at 10°C. The gel was stained for 30 min with 0.5 μg/ml ethidium bromide and scanned on FluorImager (Molecular Dynamics). After PFGE, DNA was depurinated in 0.25 N HCl for 10 min, denaturated in 0.5M NaOH and 1.5M NaCl for 20 min, neutralized in 1M ammonium acetate and 0.02M NaOH for 20 min, and transferred to a nitrocellulose membrane (Whatman Protran) by Southern Blotting. Hybridization was done with probes recognizing pausing sites on chromosomes 3 and 6.

2D Gel Electrophoresis

Cells were synchronized in G1 with α-factor at 25°C and released in media containing HU 0.2 M at 30°C. Samples were collected at the indicated time points and incubated with Sodium Azide 1% for 30 min on ice. *In vivo* psoralen crosslinking and DNA extraction with CTAB were performed as in (Giannattasio et al., 2014). DNA samples were digested with *Hind*III and *Pst*I and analyzed with probes recognizing natural pausing sites on chromosomes 3 and 10. In parallel, DNA samples were digested with NcoI and analyzed with a probe against *ARS305*.

Supplemental References

Bermejo, R., Capra, T., Gonzalez-Huici, V., Fachinetti, D., Cocito, A., Natoli, G., Katou, Y., Mori, H., Kurokawa, K., Shirahige, K., *et al.* (2009a). Genome-organizing factors Top2 and Hmo1 prevent chromosome fragility at sites of S phase transcription. Cell *138*, 870-884.

Bermejo, R., Katou, Y.M., Shirahige, K., and Foiani, M. (2009b). ChIP-on-chip analysis of DNA topoisomerases. Methods Mol. Biol. *582*, 103-118.

Branzei, D., Sollier, J., Liberi, G., Zhao, X., Maeda, D., Seki, M., Enomoto, T., Ohta, K., and Foiani, M. (2006). Ubc9- and mms21-mediated sumoylation counteracts recombinogenic events at damaged replication forks. Cell *127*, 509-522.

Fachinetti, D., Bermejo, R., Cocito, A., Minardi, S., Katou, Y., Kanoh, Y., Shirahige, K., Azvolinsky, A., Zakian, V.A., and Foiani, M. (2010). Replication termination at eukaryotic chromosomes is mediated by Top2 and occurs at genomic loci containing pausing elements. Mol. Cell *39*, 595-605.

Giannattasio, M., Zwicky, K., Follonier, C., Foiani, M., Lopes, M., and Branzei, D. (2014). Visualization of recombination-mediated damage bypass by template switching. Nat. Struct. Mol. Biol. *21*, 884-892.

Livak, K.J., and Schmittgen, T.D. (2001). Analysis of relative gene expression data using real-time quantitative PCR and the 2(-Delta Delta C(T)) Method. Methods *25*, 402-408.

Song, W., Dominska, M., Greenwell, P.W., and Petes, T.D. (2014). Genome-wide high-resolution mapping of chromosome fragile sites in Saccharomyces cerevisiae. Proc. Natl. Acad. Sci. USA *111*, E2210-2218.

Wang, Y., Vujcic, M., and Kowalski, D. (2001). DNA replication forks pause at silent origins near the HML locus in budding yeast. Mol. Cell. Biol. *21*, 4938-4948.
