# Supplementary material for: Essential Roles of the Smc5/6 Complex in Replication through Natural Pausing Sites and Endogenous DNA Damage Tolerance
Source: Mol Cell. 2015 Dec 17;60(6):835–46. doi: 10.1016/j.molcel.2015.10.023 (PMC4691243; doi:10.1016/j.molcel.2015.10.023)
Supplement: Document S2. Article plus Supplemental Information [file mmc2.pdf]

Article

Molecular Cell

Essential Roles of the Smc5/6 Complex in Replication through Natural Pausing Sites and Endogenous DNA Damage Tolerance

Graphical Abstract

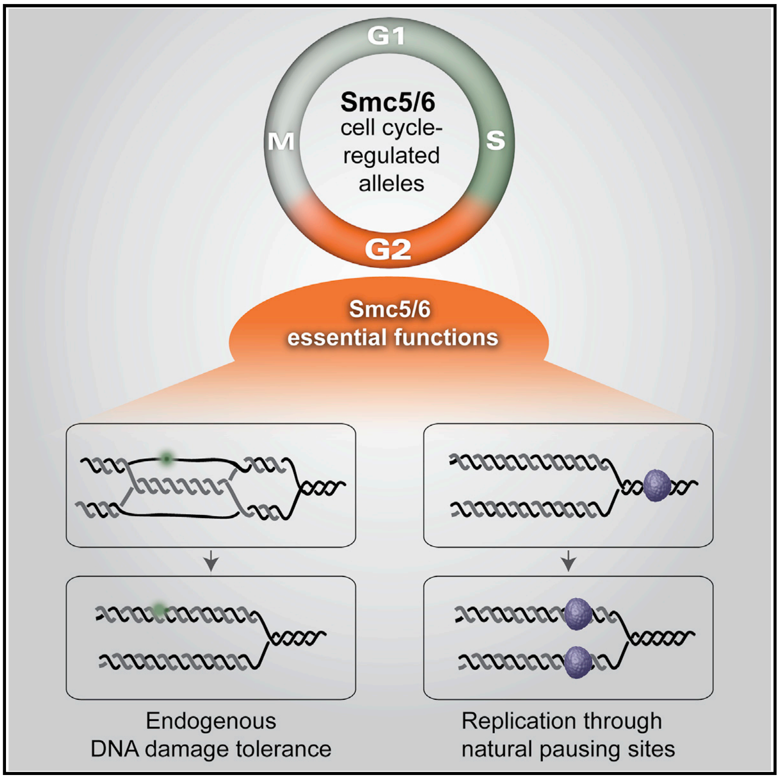

Authors

Demis Menolfi, Axel Delamarre, Armelle Lengronne, Philippe Pasero, Dana Branzei

Correspondence

dana.branzei@ifom.eu

In Brief

Menolfi et al. explore the roles of the Smc5/6 complex in proliferation. Cell-cycle-regulated alleles reveal Smc5/6 essential functions to segregate in G2/M. Genetic screens with generated alleles underpin roles for Smc5/6 in the metabolism of recombination structures triggered by endogenous replication stress, and in replication through natural pausing sites.

Highlights

- Cell-cycle-regulated alleles reveal Smc5/6-essential functions to segregate in G2/M
- Genetic screen with new hypomorphic allele identifies processes relying on Smc5/6
- Smc5/6 acts on recombination structures triggered by endogenous replication stress
- Smc5/6 prevents fragility and mediates replication through natural pausing sites

Accession Numbers

GSE72241

Menolfi et al., 2015, Molecular Cell *60*, 835–846
December 17, 2015 ©2015 The Authors
http://dx.doi.org/10.1016/j.molcel.2015.10.023

Molecular Cell
Article

Essential Roles of the Smc5/6 Complex in Replication through Natural Pausing Sites and Endogenous DNA Damage Tolerance

Demis Menolfi,[1] Axel Delamarre,[2] Armelle Lengronne,[2] Philippe Pasero,[2] and Dana Branzei[1,*]
[1]IFOM, the FIRC Institute of Molecular Oncology, Via Adamello 16, 20139, Milan, Italy
[2]IGH, Institute of Human Genetics CNRS UPR 1142, 141 rue de la Cardonille F-34396 Cedex 5, Montpellier, France
*Correspondence: dana.branzei@ifom.eu
http://dx.doi.org/10.1016/j.molcel.2015.10.023
This is an open access article under the CC BY license (http://creativecommons.org/licenses/by/4.0/).

SUMMARY

The essential functions of the conserved Smc5/6 complex remain elusive. To uncover its roles in genome maintenance, we established *Saccharomyces cerevisiae* cell-cycle-regulated alleles that enable restriction of Smc5/6 components to S or G2/M. Unexpectedly, the essential functions of Smc5/6 segregated fully and selectively to G2/M. Genetic screens that became possible with generated alleles identified processes that crucially rely on Smc5/6 specifically in G2/M: metabolism of DNA recombination structures triggered by endogenous replication stress, and replication through natural pausing sites located in late-replicating regions. In the first process, Smc5/6 modulates remodeling of recombination intermediates, cooperating with dissolution activities. In the second, Smc5/6 prevents chromosome fragility and toxic recombination instigated by prolonged pausing and the fork protection complex, Tof1-Csm3. Our results thus dissect Smc5/6 essential roles and reveal that combined defects in DNA damage tolerance and pausing site-replication cause recombination-mediated DNA lesions, which we propose to drive developmental and cancer-prone disorders.

INTRODUCTION

Genomic instability, a hallmark of cancer cells, is induced by DNA damage and replication stress. DNA damage response (DDR) mechanisms act as crucial barriers against genetic instability and are mediated by conserved pathways that ensure DNA damage recognition and repair (Branzei and Foiani, 2008).

The Structural Maintenance of Chromosomes (SMC) complex Smc5/6, related to cohesin and condensin, contributes to DNA repair (Lehmann, 2005). Smc5/6 also facilitates organization and segregation of repeat elements and binds to topological intermediates generated during replication (Jeppsson et al., 2014b; Murray and Carr, 2008). However, to what extent these functions contribute to its important/essential roles in cellular proliferation remains unknown. Notably, Smc5/6 contains a SUMO ligase, Mms21/Nse2, which is important for the described functions of the complex (Bermúdez-López et al., 2015; Branzei et al., 2006; Zhao and Blobel, 2005), and was recently identified to be mutated in a developmental disorder characterized by primordial dwarfism (Payne et al., 2014).

As an SMC complex, Smc5/6 is bound to exert its chromosome metabolism functions while being associated with chromatin (Jeppsson et al., 2014b). In this regard, previous studies outlined distinct binding patterns for Smc5/6 on chromosomes during and after DNA replication (Lindroos et al., 2006). In S phase, Smc5/6 associates with origins of replication (Bustard et al., 2012), whereas in G2/M, Smc5/6 binds to centromeres and various chromosome arm locations (Lindroos et al., 2006) in a manner dependent on sister chromatid cohesion and correlating with the presence of topological intermediates known as precatenanes or sister chromatid intertwinings (Jeppsson et al., 2014a; Kegel et al., 2011). However, the roles performed by Smc5/6 when bound to such chromosome addresses are not well understood.

Here we set out to investigate Smc5/6 roles in proliferation. Using *Saccharomyces cerevisiae* cell cycle-regulated alleles that enable restriction of Smc5/6 subunits to S or G2/M, we identified that Smc5/6 essential functions are manifested selectively in G2/M. Employing genome-wide genetic screens made possible by newly generated *smc5/6* alleles, we further uncovered pathways that compensate for Smc5/6 deficiency in G2/M. Our results reveal crucial roles for Smc5/6 in processing DNA structures formed in the context of endogenous error-free DNA damage tolerance (DDT) and in facilitating replication through natural pausing sites predisposed to fragility (Song et al., 2014; Tourrière and Pasero, 2007). The findings also reveal important interplay between Smc5/6 and other genome caretakers in chromosome metabolism processes that are crucial for proliferation and genome integrity.

RESULTS

Smc5/6 Restriction to G2/M Allows Functional Complex Assembly and Chromosome Association

To address Smc5/6 S phase functions, we limited to G2/M the expression of individual genes encoding for six components of the octameric Smc5/6, namely *SMC5*, *SMC6*, *NSE1* (encoding

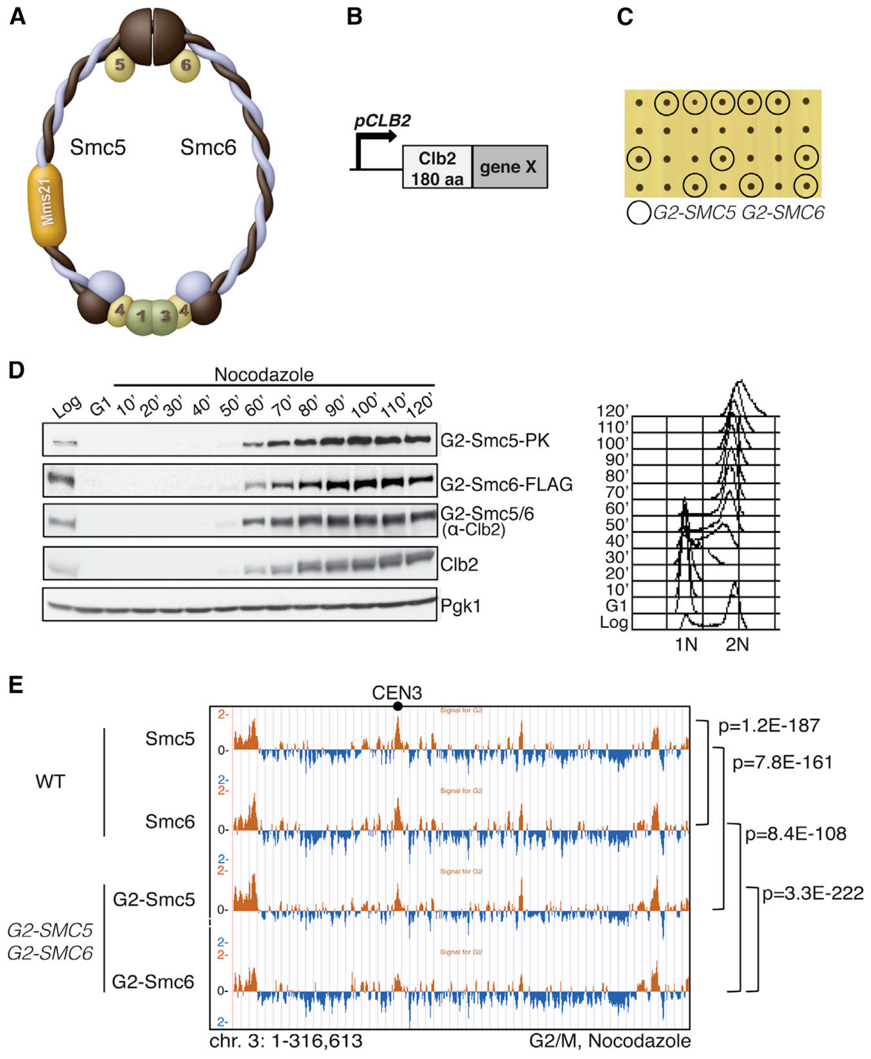

Figure 1. Smc5/6 Restriction to G2/M Allows Normal Proliferation and Smc5/6 Chromatin Localization in Metaphase Cells

(A and B) Schematic representation of the octameric Smc5/6 complex (A) and of the Clb2-derived G2-tag (B).

(C) *G2-SMC5 G2-SMC6* cells, derived from crosses between *G2-SMC5* and *G2-SMC6*, are viable.

(D) *G2-SMC5* and *G2-SMC6* expression is coincident with the one of *CLB2* and restricted to G2/M. Pgk1 serves as loading control.

(E) ChIP-on-chip profile of Smc5-PK, Smc6-FLAG, and G2-Smc5-PK, G2-Smc6-FLAG from G2/M-synchronized WT and *G2-SMC5 G2-SMC6* cells, respectively. Chromosome 3 is shown as example. The indicated p values relate to the genome-wide overlap between the considered ChIP-on-chip protein clusters.

See also Figure S1.

a putative ubiquitin ligase), *MMS21/NSE2* (encoding for a SUMO ligase), *NSE4* (encoding the subunit that connects the globular heads of Smc5 and Smc6), and *NSE5* (encoding one of the two subunits associated with the hinge domains of Smc5/6) (Figure 1A). To these ends, we swapped the individual promoters of the above-mentioned genes with a modified form of the mitotic cyclin Clb2 promoter and the N-terminal part of Clb2 that includes its degrons (Figure 1B), referred to as the G2-tag (Karras and Jentsch, 2010). To avoid difficulties in scoring lethality if Smc5/6 functions were essential during DNA replication, we constructed diploid cells heterozygous for the allele under study and recovered haploid cells with the desired G2-allele by tetrad dissection (see Figure S1A available online, and see below). Notably, the G2-tag did not interfere with cell viability and fitness for any of the six Smc5/6 components analyzed, although the restriction to G2/M was in all cases successful (Figure S1B and data not shown).

Smc5/6 associates with replication forks stalled by hydroxyurea (HU) (Bustard et al., 2012). To examine if Smc5 still binds replication forks when its SMC partner, Smc6, is restricted to G2/M, we analyzed Smc5 chromatin clusters using genome-wide chromatin immunoprecipitation (ChIP) studies (ChIP-on-chip) in HU-treated wild-type (WT) and *G2-SMC6* cells. We found that Smc5 binds in the proximity of replication forks (Figure S1C), well in line with previous reports (Bustard et al., 2012), but it does not show the bimodal distribution around replication origins characteristic of other SMC proteins, such as cohesin and Rad50 (Tittel-Elmer et al., 2012). Differently from WT, we observed almost no binding to chromatin for Smc5 in *G2-SMC6* cells (Figure S1C). Importantly, in the same strains, Smc5 chromatin clusters in G2/M were qualitatively identical in WT and *G2-SMC6* cells, indicating that Smc5/6 complexes containing the G2-Smc6 protein can efficiently assemble postreplicatively (Figure S1D).

To examine a context in which the existence of Smc5/6-like complexes consisting of Smc5 or Smc6 homodimers can be fully excluded early in S phase, we constructed a double mutant *G2-SMC5 G2-SMC6*. Notably, these cells were also characterized by normal growth (Figure 1C), although the G2-Smc5/6 variants were absent in S phase (Figure 1D). Moreover, G2/M-restricted Smc5/6 variants were correctly degraded in anaphase as predicted by the G2-tag (Figure S1E). Importantly, G2-Smc5-PK and G2-Smc6-FLAG chromatin clusters showed genome-wide statistically significant overlap to Smc5-PK and Smc6-FLAG from the corresponding WT cells in G2/M (Figure 1E). These observations demonstrate that G2-Smc5 and G2-Smc6 proteins can be assembled in a functional Smc5/6 complex in G2. Together, our observations indicate that the roles of Smc5/6 in S phase do not measurably impact on proliferation and are not essential for chromatin positioning and functions of Smc5/6 in G2/M.

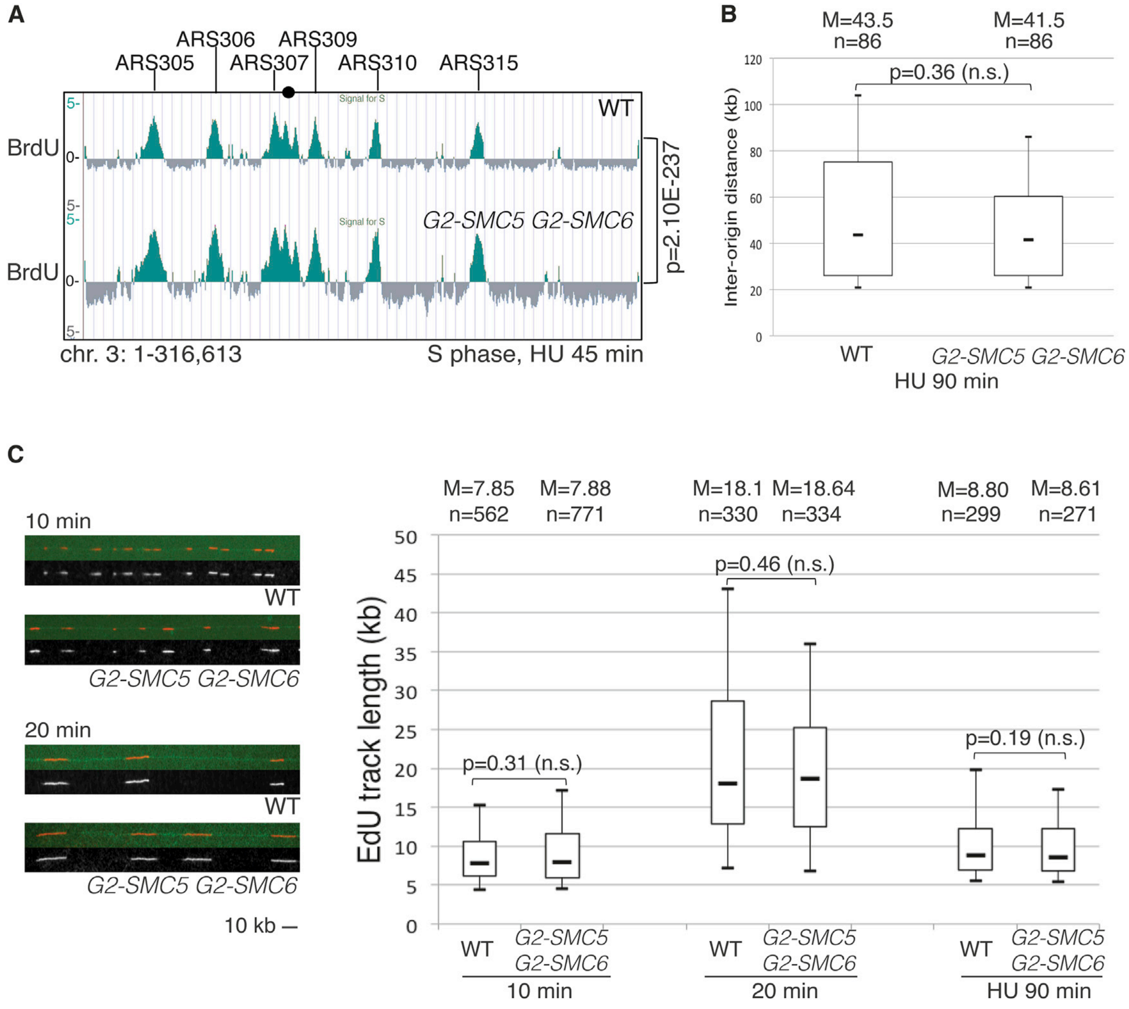

Figure 2. Smc5/6 Restriction to G2/M Allows Normal Origin Firing and Replication Fork Speed

(A) BrdU IP-on-chip profile in WT and *G2-SMC5 G2-SMC6* cells synchronously released in media containing HU and BrdU. Chromosome 3 is shown with annotated early *ARS* regions. The p value of the genome-wide overlap between the BrdU clusters in the two strains is indicated.

(B) WT and *G2-SMC5 G2-SMC6* were synchronized in G1 and released in S phase in the presence of EdU and HU. Box plot analysis of the interorigin distance (IOD) in the two samples. For each strain, the median (M) value and the number (n) of the IODs analyzed are indicated. The p value, calculated with the Mann-Whitney test, indicates nonsignificant differences between WT and *G2-SMC5 G2-SMC6*.

(C) Asynchronous WT and *G2-SMC5 G2-SMC6* cells were treated with EdU, and cells were collected at 10 and 20 min afterward for molecular combing analysis. Alternatively, WT and *G2-SMC5 G2-SMC6* were synchronized in G1 and released in S phase in the presence of EdU and HU for 90 min. Examples of EdU tracks at 10 min and 20 min are reported. The box plot indicates the statistical analysis of the EdU-track length as in (B).

See also Figure S2.

G2/M Restriction of Smc5/6 Allows Normal Replication Initiation and Replication Fork Speed

We further addressed if absence of Smc5/6 during S phase affects the replication program and replication fork speed. Genome-wide BrdU profiles revealed that the origin firing pattern and the firing efficiency were qualitatively identical in WT and *G2-SMC5 G2-SMC6* cells replicating in the presence of HU (Figure 2A). Consistent with this result, the interorigin distance was comparable in the two strains, as calculated by molecular combing (Figure 2B). We next examined the replication fork speed of individual forks. Both in unperturbed and HU conditions, the length of the replicated tracts was highly similar between WT and *G2-SMC5 G2-SMC6* cells (Figure 2C and data not shown). Thus, S phase-specific conditional depletion of Smc5/6 does not alter the origin-firing program and fork speed.

Smc5/6 promotes tolerance of replication-associated lesions, such as those induced by methyl-methanesulfonate, MMS (Branzei et al., 2006). We found that the replicated tract lengths were similar in WT and *G2-SMC5 G2-SMC6* cells replicating in the presence of MMS (Figure S2), indicating that Smc5/6 role in MMS tolerance is largely postreplicative. We conclude that Smc5/6 can fully provide for chromosome integrity functions both in unperturbed and DNA damaging conditions if supplied in G2/M.

Smc5/6 Essential Functions Are Manifested in G2/M

Next, we analyzed the consequences of restricting the expression of Smc5/6 components to S phase, using an analogous S-tag (Figure 3A) (Hombauer et al., 2011). The S-tag utilizes the promoter and the N terminus degron elements of the S phase cyclin, Clb6, to induce expression of the modified gene early in S phase and cause degradation of the encoded protein at the end of S phase (Jackson et al., 2006). While heterozygous *S-SMC5/SMC5* cells were viable, haploid *S-SMC5* cells were not (Figure 3B). To examine if this lethality is due to impairments in *S-SMC5* expression, we analyzed the two Smc5 variants, Smc5 and S-Smc5, both C-terminally tagged with PK, in the heterozygous diploid. For this purpose, we synchronized the *S-SMC5/SMC5* diploids in G2/M and released the cells in the presence of HU and then in media containing nocodazole (Figure 3C). As expected for an S-tagged variant, S-Smc5 was detected in S phase diploids at levels comparable with WT Smc5 and declined as cells reached G2/M (Figure 3C). Next, we analyzed if S-Smc5 was still functional in S phase for binding stalled replication forks. To simultaneously analyze Smc5 and S-Smc5 binding profiles, we tagged the two *SMC5* alleles of the heterozygous diploid with different C-terminal tags. ChIP-on-chip analysis of Smc5-FLAG and S-Smc5-PK chromatin clusters in diploid cells traversing S phase revealed strong similarity and statistically significant chromatin cluster overlap (Figure 3D). Thus, successful restriction of *SMC5* expression to S phase causes lethality.

Next, we applied the S-tag to other five components of Smc5/6. We observed variation in the severity of the phenotypes, commensurate with the efficiency and timeliness of the S-tagged proteins degradation (see below). Notably, four out of the six S-tagged alleles caused either lethality, as in the case of *SMC5* (Figure 3B) and *NSE4* (Figures S3A and S3B), or severe slow growth, as in the case of *MMS21* and *NSE1* (Figures S3C–S3D and data not shown). In contrast, *S-SMC6* and *S-NSE5* haploid cells were viable (Figure 3E and data not shown). Time course analysis of S-Smc6 revealed that it was correctly produced in S phase and began to be degraded when cells started to express *CLB2*, but that low levels of S-Smc6 still persisted in G2/M (Figure 3F). Moreover, both S-Smc6 and Smc6 were proficient in binding to early origins of replication and showed qualitatively identical ChIP-on-chip profiles in HU (Figure S3E), indicating that S-Smc6 is functional in S phase. The results demonstrate that the low levels of S-Smc6 persisting in G2/M are sufficient for viability and suggest that *S-SMC6* is potentially a hypomorphic allele, selectively defective in G2/M due to low amounts of Smc6, and can be used in genetic screens to uncover essential functions of Smc5/6 complex manifested in G2/M (see below).

While the persistent low amount of Smc6 in G2/M can explain the apparent incongruence in cell viability obtained by limiting *SMC5* and *SMC6* expressions to S phase, this contention further predicts that a more severe impairment of Smc5/6 function in G2/M would be detrimental to growth. Indeed, combination of *S-SMC6* and *S-MMS21* alleles resulted in lethality (Figure 3G). Moreover, when we attempted to examine if the low levels of Smc6 present in *S-SMC6* cells (Figure 3F) allow chromatin recruitment of Smc5/6 in G2/M—which we planned to address by ChIP-on-chip analysis of Smc5 and Nse4 clusters—we found that combination of the *S-SMC6* allele with PK-tagged alleles of *SMC5* and *NSE4* resulted in very severe synthetic fitness defects (Figures 3H and S3F). These findings, together with the severe effects on viability caused by limiting *SMC5*, *NSE4*, *MMS21*, and *NSE1* expression to S phase, support the notion that Smc5/6 essential functions are manifested after bulk chromosome replication, in G2/M.

Identification of Pathways Providing for Viability in Cells with Limited Levels of Smc5/6 in G2/M

To identify pathways that act redundantly or in compensation with Smc5/6 in G2/M, we conducted robot-assisted synthetic genetic array screens with *S-SMC6* (Figure 4A). Three classes of mutations were repeatedly picked up by the screen, excluding those that appear nonselectively in other SGA screens conducted in the lab, petite mutants, and the *LGE1/BRE1* class required for S-phase cyclin gene expression (Zimmermann et al., 2011) and which likely causes lethality due to very low levels of S-Smc6. Of these, two groups comprised genes previously connected to DNA metabolism and were retained for validation and scoring for potential synthetic interactions also with *G2-SMC6*. In this way, we identified distinct pathways of chromosome integrity that are required for viability in conditions of limited amounts of Smc5/6 in G2/M. These pathways and the studies exposing their interrelation with Smc5/6 are described below.

Smc5/6 Facilitates Resolution of Recombination Structures Formed during Endogenous DNA Damage Tolerance

A group of mutants displaying synthetic sickness/lethality with *S-SMC6*, but not with *G2-SMC6*, consisted of the Sgs1-Top3-Rmi1 (STR) complex (Figures 4B, S4A, and S4B). Moreover, we found that the genome-wide clusters of Smc5/6 and Top3 had statistically significant overlap (Figure 4C and data not shown), suggesting that Smc5/6 and Top3 may act in proximity and/or have common substrates. Both Sgs1 and Smc5/6 have been tightly linked to the processing of DNA damage tolerance (DDT) template switch intermediates induced by DNA damage (Branzei et al., 2006, 2008; Choi et al., 2010; Sollier et al., 2009). Efficient formation of template switch intermediates depends on both Rad51 and Rad5/Mms2/Ubc13-mediated PCNA polyubiquitylation, a feature that distinguishes template switching from other recombination-dependent processes. Interestingly, we found that *rad51Δ*, as well as *rad5Δ*, *ubc13Δ*, and *mms2Δ* mutations, rescued the synthetic lethality of *S-SMC6 sgs1Δ* (Figures 4D, 4E, S4C, and S4D). Although both Smc5/6 and Sgs1 play important roles in regulating recombination at ribosomal DNA (rDNA) (Fricke and Brill, 2003; Torres-Rosell et al., 2005), we found that deletion of *FOB1*, a condition that largely suppresses rDNA recombination, did not rescue *S-SMC6 sgs1Δ* lethality (Figure 4F). Thus, the functional interaction between Smc5/6 and STR does not predominantly reflect rDNA-related events. In all, the above results expose a role for the Rad5 pathway in generating DNA substrates for STR and Smc5/6 following physiological levels of replication stress.

Two mutually nonexclusive scenarios can explain the Rad5-dependent genetic interactions observed between *S-SMC6* and *sgs1Δ* mutations. The first places Smc5/6 upstream and

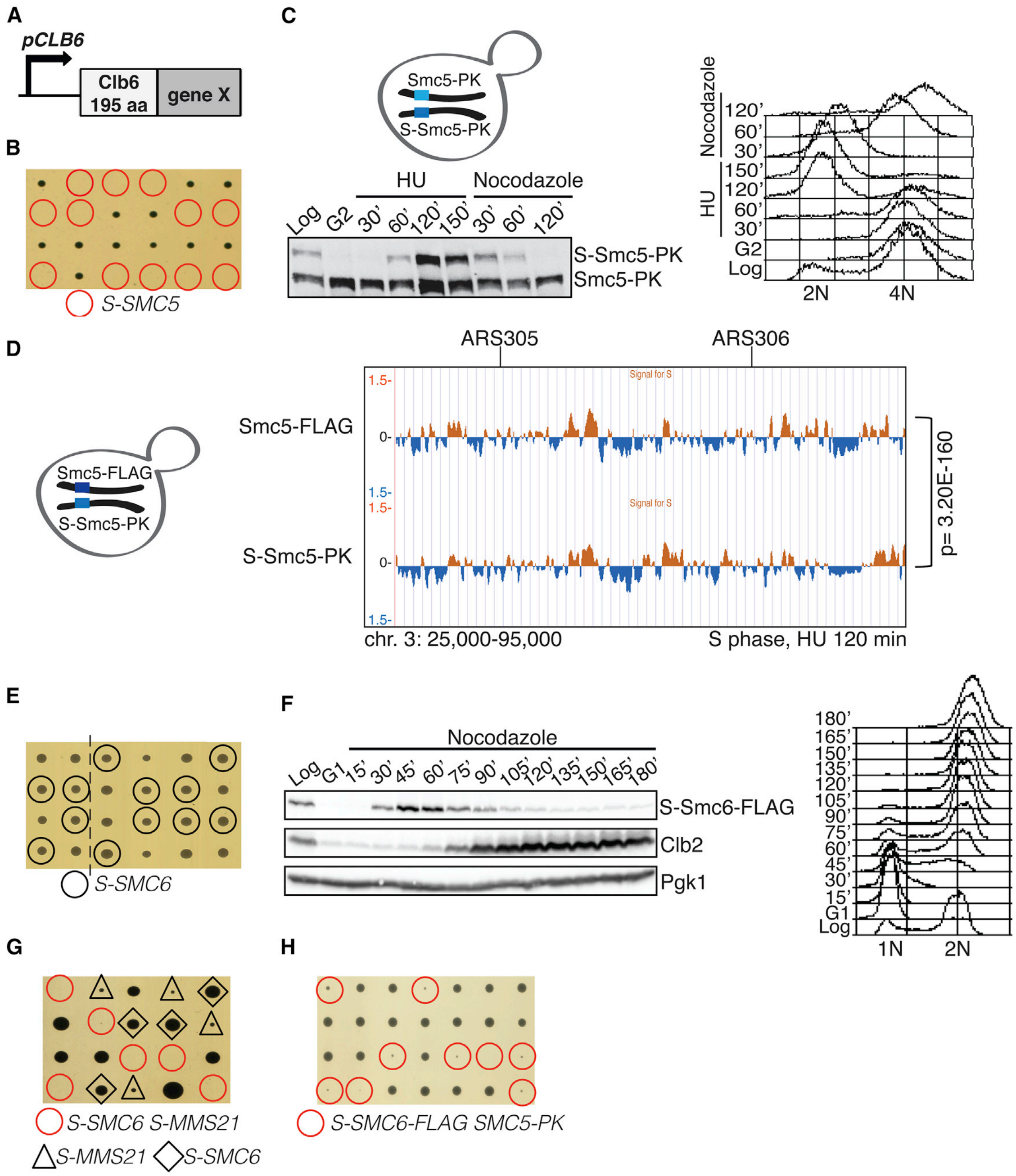

Figure 3. Restriction of Smc5/6 to S Phase Has Adverse Effects on Proliferation

(A) Schematic representation of the Clb6-derived S-tag.

(B) *S-SMC5* cells derived from sporulation of *SMC5/S-SMC5* heterozygous diploids are not viable.

(C) Expression of *SMC5-PK* versus *S-SMC5-PK*. Asynchronous *SMC5/S-SMC5* diploid cells were arrested in G2/M, released in media containing HU for 150 min, and then released from HU in media containing nocodazole for 120 min. Western anti-PK and FACS are shown.

(D) ChIP-on-chip profile of Smc5-FLAG and S-Smc5-PK from diploid *SMC5-FLAG/S-SMC5-PK* cells released from G2/M arrest in media containing HU for 120 min. A snapshot of chromosome 3 is reported; p value of genome-wide overlap of clusters is indicated.

(E) *S-SMC6* cells are viable. The line indicates elimination of superfluous lanes from the tetrad dissection plate image.

(F) *S-SMC6-FLAG* expression was monitored by western blotting using G1 cells released in media containing nocodazole. Clb2 and Pgk1 (loading control) blots are also shown.

(G and H) *S-SMC6 S-MMS21* and *S-SMC6-FLAG SMC5-PK* obtained by tetrad dissection are not viable.

See also Figure S3.

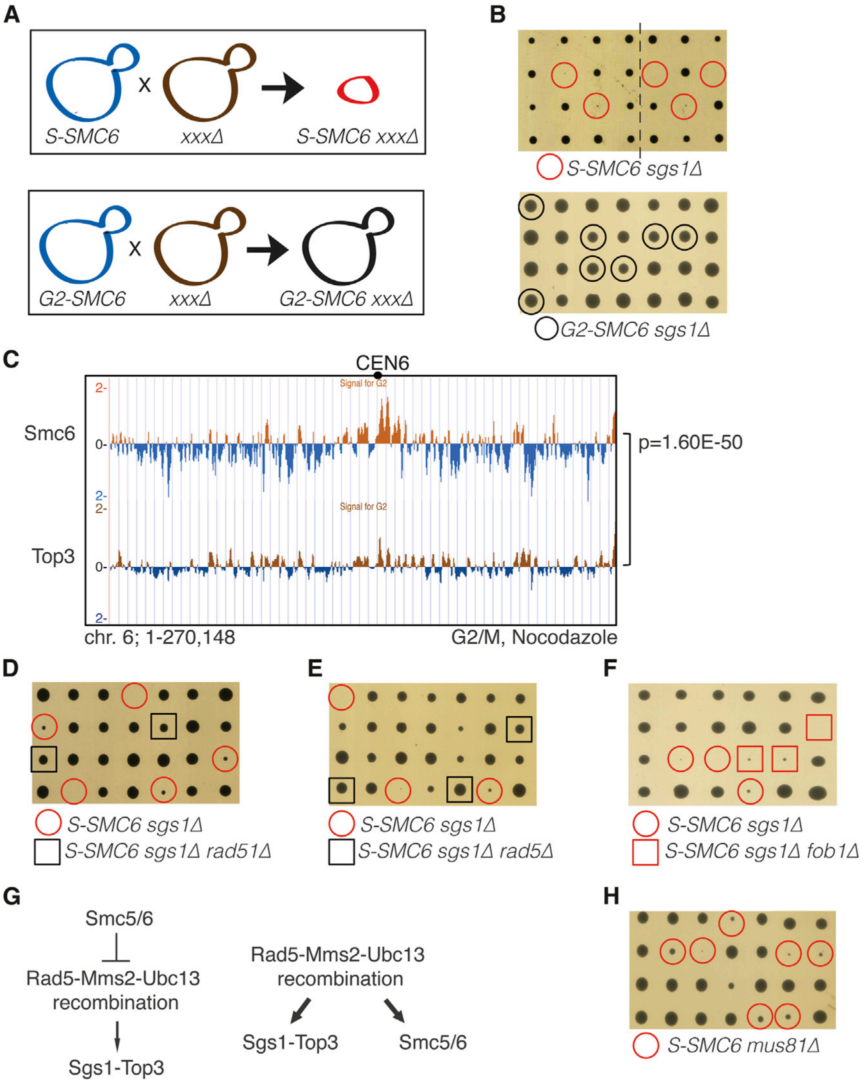

Figure 4. Functional Interaction between Smc5/6 and Sgs1-Top3-Rmi1 in G2/M

(A) Synthetic genetic array screens conducted between *S-SMC6* and the yeast nonessential knockout library.

(B) *S-SMC6*, but not *G2-SMC6*, is synthetic lethal with *sgs1Δ*. The line indicates elimination of superfluous lanes from the tetrad dissection plate image.

(C) ChIP-on-chip profiles of Smc6-FLAG (the same as in Figure 1E) and Top3-FLAG from G2/M-arrested cells. Chromosome 6 is shown as example; p value of genome-wide overlap of the considered protein clusters is indicated.

(D–F) Individual deletions of *RAD51* and *RAD5*, but not of *FOB1*, rescue *S-SMC6 sgs1Δ* lethality.

(G) Two scenarios of Smc5/6 functions in relation to Rad5- and Rad51-dependent recombination structures formed in unperturbed conditions.

(H) *S-SMC6* is synthetic lethal/sick with *mus81Δ*.

See also Figure S4.

as counteracting the Rad5 pathway responsible for producing recombination substrates (Figure 4G, left panel). In the second, Smc5/6 facilitates resolution of Rad5-dependent structures, jointly or in parallel with STR (Figure 4G, right panel). Both scenarios entail an increase in recombination-mediated DDT structures in the absence of Sgs1 and Smc5/6 that would impair chromosome segregation if left unresolved (Matos et al., 2011; Szakal and Branzei, 2013). Judging from the ability of Smc5/6 to promote resolution of damage-induced recombination structures when activated in G2/M (Bermúdez-López et al., 2010), the observation that Rad5 mediates template switching in S phase (Karras et al., 2013), and that the G2/M but not the S phase Smc5/6 function is required for viability in *sgs1Δ* (Figure 4B), the model placing Smc5/6 action downstream of the Rad5 step in processing endogenous recombination DDT intermediates appears most probable (Figure 4G, right panel).

Additional evidence we obtained substantiates the above view. Specifically, we found that similarly to *sgs1Δ*, the *S-SMC6* mutation causes slow growth in *pol32Δ* cells (Figure S4E), mutated in the noncatalytic subunit of replicative polymerase δ and suffering of replication stress associated with activation of the error-free DDT branch (Karras and Jentsch, 2010). If the Rad5-dependent DNA structures requiring Smc5/6 during unperturbed proliferation are akin to those triggering template switching after DNA damage, inactivation of Smc6 in G2/M may cause an enhanced requirement for alternate resolution mediated by Mus81-Mms4 and by the Slx4 scaffold (Ashton et al., 2011; Gritenaite et al., 2014; Szakal and Branzei, 2013). Supporting this hypothesis, *S-SMC6* was slow-growing in combination with *mus81Δ*, *mms4Δ*, and *slx4Δ* (Figure 4H and data not shown). In all, the results provide evidence for endogenous Rad5 and PCNA polyubiquitylation-dependent DDT operating during unperturbed replication, and for a postreplicative role of Smc5/6 in recombination-mediated DDT activated by endogenous replication stress.

Smc5/6 Functionally Cooperates with Rrm3 at Natural Pausing Sites

Besides the STR mutant group, we identified that deletion of *RRM3* is sick/lethal in combination with *S-SMC6*, but not with *G2-SMC6* (Figure 5A). Rrm3 is a DNA helicase that facilitates fork passage through nonhistone protein-DNA complexes and through natural pausing sites, also referred to as replication fork barriers (RFBs) when they are located in the rDNA region (Azvolinsky et al., 2009; Ivessa et al., 2003; Mohanty et al., 2006). We found a statistically significant overlap between Smc5/6 and Rrm3 chromatin clusters in G2/M (Figure 5B). Moreover, similarly to Rrm3, Smc5/6 was enriched at tRNA and centromere (CEN) regions (Figures 5C and S5A), *HML* mating-type locus (Figure S5B), and pausing sites that serve as

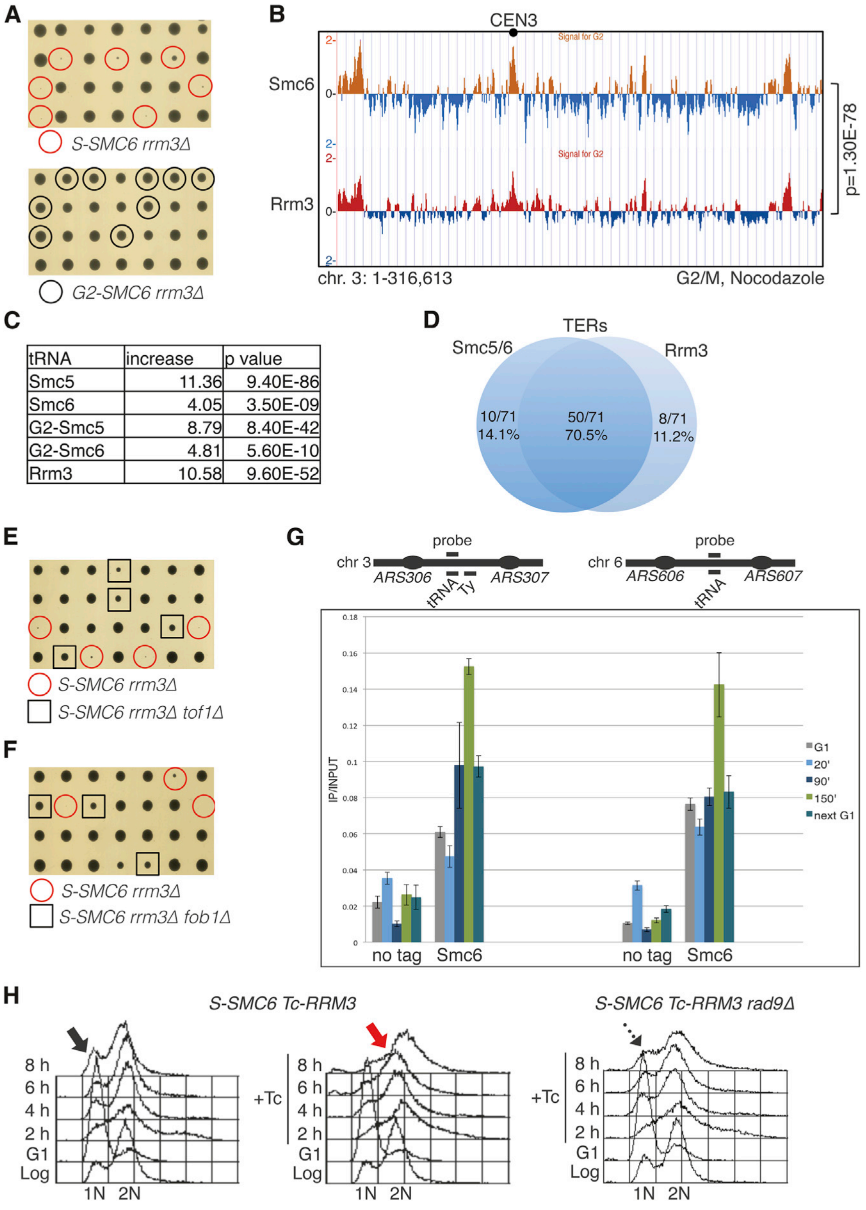

tRNA	increase	p value
Smc5	11.36	9.40E-86
Smc6	4.05	3.50E-09
G2-Smc5	8.79	8.40E-42
G2-Smc6	4.81	5.60E-10
Rrm3	10.58	9.60E-52

Figure 5. Smc5/6 and Rrm3 Are Enriched at Natural Pausing Sites and Counteract the Toxicity Associated with Tof1/Cms3- and Fob1-Mediated Pausing

(A) *S-SMC6*, but not *G2-SMC6*, is synthetic lethal/sick with *rrm3Δ*.

(B) ChIP-on-chip of Smc6-FLAG (the same as in Figure 1E) and Rrm3-FLAG from G2/M-synchronized cells. Chromosome 3 is shown and p value of the genome-wide overlap of clusters indicated.

(C) Smc5, Smc6, G2-Smc5, G2-Smc6, and Rrm3 are significantly enriched at tRNA genes. The table reports the fold increase of each protein at tRNA genes, calculated versus the ones expected for random binding, and the p values of the significance.

(D) Smc5, Smc6, and Rrm3 are enriched at pausing sites that serve as termination sites (TERs) in G2/M. Percentage of overlap and nonoverlap is shown.

(E and F) Individual deletions of *TOF1* and *FOB1* rescue *S-SMC6 rrm3Δ* synthetic lethality.

(G) ChIP-qPCR-monitored binding of Smc6-FLAG at pausing sites on chromosomes 3 and 6, also known as TER302 and TER603 (Fachinetti et al., 2010). The samples were collected in G1, at 20 min (early S phase), 90 min (late S phase/G2), and 150 min (G2/M) after release from G1 arrest in media containing nocodazole, and again in the following G1, after nocodazole removal and a second synchronization with α-factor.

(H) *S-SMC6 Tc-RRM3* cells were synchronized in G1 and released in the absence or presence of tetracycline (Tc) for 8 hr. Samples for FACS analysis were collected every 2 hr. *S-SMC6 Tc-RRM3 rad9Δ* were analyzed in the presence of Tc.

See also Figure S5 and Table S1.

termination sites (TERs) during replication (Figure 5D; Table S1) (Fachinetti et al., 2010).

The conserved replisome-associated fork protection complex Tof1-Csm3 enforces pausing at pausing sites genome-wide (Calzada et al., 2005; Hodgson et al., 2007; Tourrière et al., 2005), and Fob1 strengthens RFB activity specifically at rDNA regions (Mohanty et al., 2006). Importantly, we found that the sickness/lethality of *S-SMC6 rrm3Δ* was rescued by individual deletions of *TOF1* and *CSM3* (Figures 5E and S5C), as well as by *FOB1* deletion (Figure 5F). This indicates that prolonged pausing and unfinished replication at rDNA (Torres-Rosell et al., 2007), which represents 8%–12% of the yeast genome, is the main contributor to the observed lethality.

Notably, however, we found Smc5/6 to be enriched at pausing sites situated outside the rDNA (Figures 5C, 5D, S5A, and S5B). Moreover, while the binding of Smc5/6 to RFBs located within rDNA was observed throughout the cell cycle (Figure S5D), we found that Smc5/6 binding to other natural pausing sites increased in late S-G2/M before declining in mitosis (Figure 5G), in parallel with an important role for Smc5/6 in *rrm3Δ* mutants manifested specifically after the end of bulk replication (Figure 5A). In addition, G2-restricted Smc5/6 variants were also enriched at natural pausing sites genome-wide (Figures 5C and S5A), indicating that this recruitment reflects a requirement for Smc5/6 at late stages of replication when most natural pausing sites are being replicated. Thus, Rrm3 and Smc5/6 colocalize to a large fraction of natural pausing sites and functionally complement each other.

To explore the molecular mechanisms underlying the growth defects in *S-SMC6 rrm3Δ* cells, we constructed conditional *S-SMC6 Tc-RRM3* cells. Inactivation of *RRM3* in *S-SMC6* by addition of tetracycline (Tc) caused prolonged G2/M arrest, as revealed by the prominent 2N DNA peak in the double mutant (Figure 5H). Deletion of genes with G2/M DNA damage checkpoint functions, such as *RAD9* and *DDC1*, but not of the spindle assembly checkpoint *MAD2*, largely alleviated the cell cycle defect of

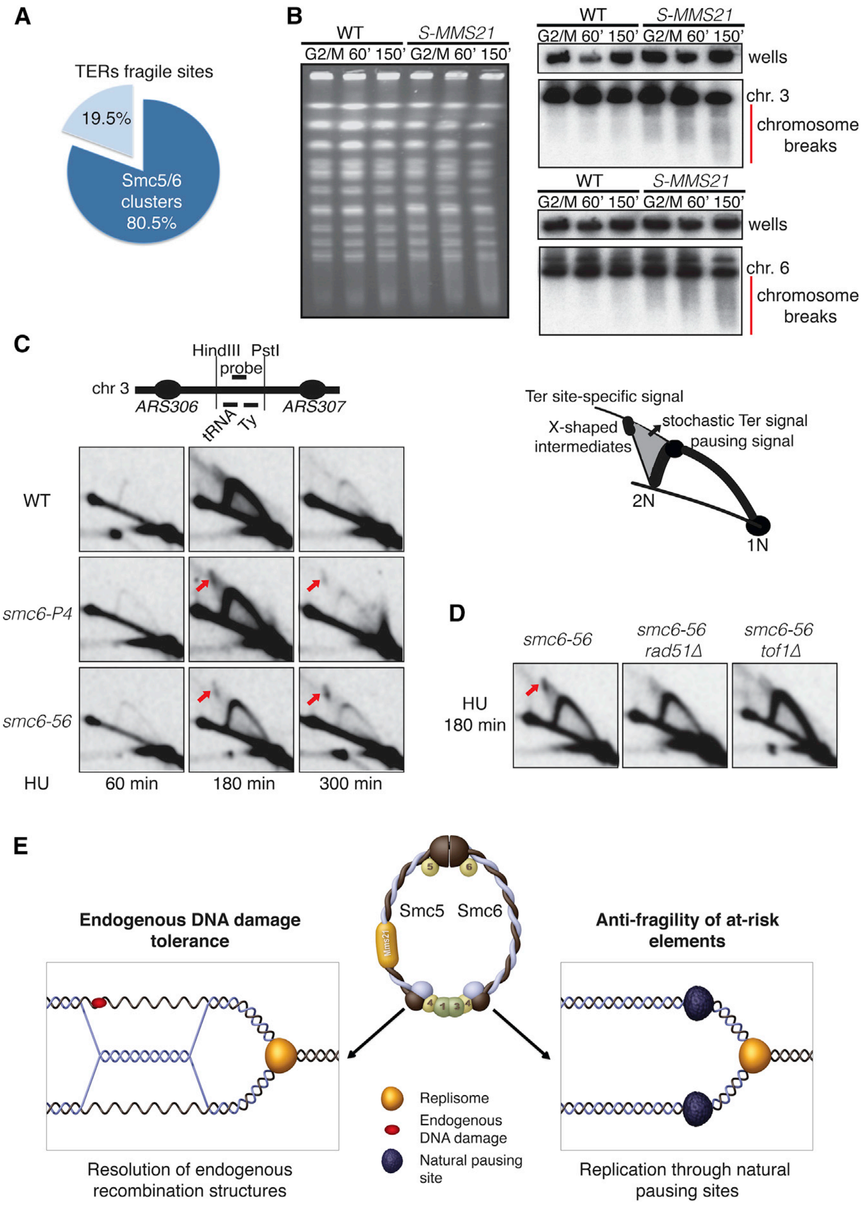

Figure 6. Smc5/6 Prevents Chromosome Fragility and Accumulation of Rad51- and Tof1-Dependent Recombination-like Structures at Natural Pausing Sites

(A) Smc5/6 binds to 80.5% of the TERs identified as fragile sites in Song et al. (2014).

(B) WT and *S-MMS21* cells were synchronized in G2/M and released in the following cell cycle in the presence of α-factor for 150 min. Samples for PFGE were collected at the indicated time points, and chromosomes were probed for regions containing natural pausing sites on chromosomes 3 and 6 represented in Figure 5G. The smearing in the gel highlighted by the red bar is representative of chromosome breakage.

(C) WT, *smc6-P4*, and *smc6-56* were synchronously released from G1 arrest in S phase in the presence of HU at 30° for 2D gel analysis. Schematic representations of the 2D gel fragment analyzed and of the type of intermediates revealed by 2D gel electrophoresis.

(D) *smc6-56*, *smc6-56 rad51Δ*, and *smc6-56 tof1Δ* were analyzed as in (C) at 180 min in HU following release from a G1 arrest.

(E) Model summarizing the identified Smc5/6 roles that support proliferation.

See also Figure S6.

S-SMC6 rrm3 conditional mutants (Figures 5H and S5E). As unfinished replication at rDNA does not trigger activation of the DNA damage checkpoint (Torres-Rosell et al., 2007), and Smc5/6 associates to pausing sites located outside rDNA, the results pinpoint a general role for Smc5/6 at natural pausing sites in preventing the formation of DNA lesions that can be sensed by the DNA damage checkpoint and cause mitotic delays.

Smc5/6 Protects Natural Pausing Sites against Recombination-Mediated Fragility

While replication termination may not be generally associated with replication fork pausing (Dewar et al., 2015), a subset of natural pausing sites that serve as replication termination regions (TERs) (Fachinetti et al., 2010) were recently identified to represent hot spots of replication-associated chromosome fragility (Song et al., 2014). We found that 80% of the identified fragile TERs (Song et al., 2014) are enriched for Smc5/6 (Figure 6A; Table S1). Moreover, based on constitutively high levels of DNA damage in WT cells (Szilard et al., 2010), previous studies identified genomic loci equivalent of mammalian fragile sites in budding yeast. Interestingly, this set of fragile sites includes pausing sites such as tRNAs and mating type loci at which we found Smc5/6 to be enriched (Figures 5C and S5B).

To examine if limiting amounts of Smc5/6 in G2/M predispose to chromosome fragility, we analyzed chromosomes by pulse-field gel electrophoresis in *S-MMS21* cells (Figure S3C), more severely affected in the Smc5/6 function than *S-SMC6* (Figure 3E). Importantly, while we did not observe any major alterations in the chromosomes following staining of gels with ethidium bromide, Southern blot analysis revealed increased chromosome breakage, observed as smearing, in G2/M (Figure 6B and data not shown).

As replication-associated fragility is often associated with recombination-mediated genome rearrangements (Song et al., 2014), we addressed by 2D gel if *smc5/6* mutants previously characterized, *smc6-P4* and *smc6-56*, display an altered pattern of replication/recombination DNA structures at natural pausing sites while replicating at a permissive temperature. We chose a well-characterized region containing several DNA replication-pausing elements—tRNA, long terminal repeat (LTR) sequence, and Ty (Figure 6C)—and at which site-specific pausing,

termination, and fragility were previously detected (Deshpande and Newlon, 1996; Fachinetti et al., 2010; Song et al., 2014). As chromosome breakage and recombination at pausing sites are relatively rare events (Calzada et al., 2005), but fragility is enhanced by slowing down of replication forks with various agents (Yunis et al., 1987), we added HU to increase the likelihood of detecting potential abnormalities. We used in vivo psoralen-crosslinking to prevent branch migration or other transitions during genome extraction that may obscure interpretation of the results. Replication started and proceeded with similar kinetics in all strains (Figure S6A), and replication structures and termination signals appeared efficiently (Figure 6C). Interestingly, both *smc6-P4* and *smc6-56*, but not WT cells, showed replication-associated accumulation of specific X-shaped signals (HU 3 hr) that persisted even when most cells have completed replication of the region (HU 5 hr) (Figure 6C). A similar accumulation of X-shaped intermediates was also visible in *S-MMS21* cells after 5 hr in HU (Figure S6B). Moreover, another analyzed pausing site, proximal to the centromeric region on chromosome 10 and at which we observed Smc5/6 to be enriched, showed an accumulation of X-shaped structures in *smc5/6* mutants at 5 hr in HU (Figure S6C). Importantly, the X-shaped structures, but not other replication intermediates, were dependent on Rad51 and Tof1 in both *smc6-56* and *smc6-P4* cells (Figure 6D and data not shown). To investigate genetically if these recombination events are toxic, we analyzed if, similarly to *TOF1* deletion (Figure 5E), *rad51Δ* also suppressed the lethality of *S-SMC6 rrm3Δ* cells. The triple mutants *S-SMC6 rrm3Δ rad51Δ* were viable but slow growing (Figure S6D), likely because of Rad51-dependent functions that contribute to viability in *smc5/6* mutants (Bustard et al., 2012).

Next, we analyzed if the detected recombination structures accumulating in the analyzed *smc5/6* mutants (Figures 6C, S6B, and S6C) are specific to pausing elements or are due to general stalling of replication forks caused by HU. For this purpose, we probed the genomic DNA to analyze the intermediates arising at an early efficient origin of replication, *ARS305*. Also at this locus, X-shaped structures that were not detected in WT cells accumulated in *smc5/6* mutants at late time points in HU (Figure S6E). Importantly, however, the X-shaped intermediates that formed in the proximity of origins (Figure S6E) were dependent on Rad51, but not on Tof1 (Figure S6F). Based on these results, we propose that Smc5/6 is recruited to natural pausing sites where it exerts an antifragility function by preventing prolonged Tof1-mediated fork pausing and associated toxic recombination events.

DISCUSSION

Differently from related SMC complexes, cohesin and condensin, the Smc5/6 functions implicated in proliferation remain hardly understood. Here, by generating cell-cycle-regulated *smc5/6* alleles that separate bulk replication functions from the ones performed later, we unveiled that the essential functions of Smc5/6 are manifested in G2/M. Using genome-wide genetic screens and selectively defective alleles, we underpinned two processes that crucially rely on Smc5/6 functions in G2/M: metabolism of endogenous DDT structures formed in response to replication stress and replication through natural pausing sites (Figure 6E). Interestingly, although both these processes relate to replication, our results indicate that the steps happening post-replicatively and at the very late stages during replication are particularly at risk and require the G2/M function of Smc5/6.

Recently, compound mutations in the *MMS21* component of Smc5/6 were shown to lead to primordial dwarfism (Payne et al., 2014), a condition observed in several replication disorders, such as Meier-Gorlin, caused by mutations in the prereplication complex (Bicknell et al., 2011). As previous work indicated that Smc5/6 associates with replicating chromatin and origins of replication (Bustard et al., 2012; Lindroos et al., 2006), a role for Smc5/6 in replication initiation or modulating fork speed would have provided a simple molecular rationale for the global growth impairments observed in *MMS21* mutated patients (Payne et al., 2014). Differently from this scenario, we found that Smc5/6 roles during S phase can be postponed to G2/M without overt perturbations in cell proliferation, replication program, or fork speed (Figures 1 and 2). Considering that origins of replication often behave as fragile sites (Di Rienzi et al., 2009), and our finding that Smc5/6 prevents fragility and recombination at natural pausing sites (Figures 6B and 6C), we propose that the binding of Smc5/6 to stalled replication forks in the proximity of origins is akin to its antifragility function at pausing sites (Figure 6E).

Growth impairments are often a common feature of DDR disorders caused by mutations in DNA repair factors (O'Driscoll et al., 2003), and defects in endogenous DDT were proposed to cause developmental problems (Klingseisen and Jackson, 2011). However, the DNA repair impairments associated with causal mutations in DDR syndromes are generally studied following treatments with ionizing radiation or various carcinogens that induce DNA damage and coincident activation of various stress response pathways. Here we provide evidence that Smc5/6 is required for DDT induced by endogenous replication stress associated with activation of Rad5/Ubc13/Mms2-mediated PCNA polyubiquitylation. In this context, Smc5/6 is crucial to compensate for deficiencies in the RecQ helicase Sgs1/BLM, mutated in cancer-prone Bloom syndrome patients, likely by its role in modulating resolution or remodeling of the emerging recombination intermediates (Figure 4G). When the postreplicative Smc5/6 levels are low, cells rely more on structure-specific endonucleases (Figure 4H) that can introduce crossovers and cause genome rearrangements (Gritenaite et al., 2014; Szakal and Branzei, 2013), indicating that low levels of Smc5/6 can predispose in the long run to genomic alterations characteristic of cancers. Interestingly, *SMC5* was recently identified as one of the genes with roles in brain metastasis development when mutated (Saunus et al., 2015). Our results indicate that this may reflect Smc5 ability to limit recombination events associated with genome landscape changes (Figure 6E). These roles of Smc5/6 may not be limited to regulating resolution of DDT recombination intermediates, but likely involve Smc5/6's ability to prevent the formation/accumulation of toxic recombination structures at specific genomic elements that are predisposed to fragility or structural changes. Indeed, our present findings highlight that Smc5/6 is important for endogenous stress pathways that are not strictly linked to DDT. One such pathway we identified and

characterized in this study is related to late-replicating zones containing natural pausing sites.

Common fragile sites (CFSs) and corresponding regions in yeast replicate very late and are prone to fragility (Cha and Kleckner, 2002; El Achkar et al., 2005; Song et al., 2014). Chromosome rearrangements at CFS drive tumor progression, but the role of these regions in normal cells is less understood. CFSs are under the crucial surveillance of the DNA damage-sensing kinase ATR (Mec1 in budding yeast) (Casper et al., 2002; Cha and Kleckner, 2002), mutated in Seckel syndrome and also characterized by primordial dwarfism (O'Driscoll et al., 2003). That the damage and replication checkpoint would monitor completion of replication at CFS is in apparent contrast with the proposed inability of the replication checkpoint to monitor incomplete replication at rDNA containing genetically programmed pausing sites (Torres-Rosell et al., 2007), and with the observation that Mec1/ATR is not required for the integrity of forks paused by RFBs (Calzada et al., 2005). However, based on our studies on Smc5/6 roles and differential recruitment at pausing sites located within rDNA and elsewhere, we propose that the two conclusions can be reconciled by considering that the chromatin environment and the nature of stalling at RFBs within rDNA are different from the ones of other natural pausing sites where single-stranded (ss) DNA formation and checkpoint activation may precede chromosome breakage (Feng et al., 2011).

Our study identifies Smc5/6 as an important factor in protecting the integrity of natural pausing sites. Besides constitutive roles in rDNA stability and at the numerous RFBs contained within the rDNA region, we find that Smc5/6 is recruited to a large fraction of known natural pausing sites late during replication (Figures 5C, 5D, and 5G). Deceleration in Smc5/6 function in G2/M increases genome fragility (Figure 6B), which further correlates with increased recombination events at natural pausing sites mediated by the replication fork protection complex, Tof1-Csm3 (Figures 6C and 6D). Importantly, the Tof1-dependent recombination events at natural pausing sites occur selectively in *smc6* mutants, but not in WT (Figures 6C and 6D). Because Tof1-Csm3 prevents fork rotation associated with precatenation at pausing sites (Schalbetter et al., 2015), it is possible that Tof1-restricted topological transitions may influence recombination and subsequent fragility at those regions. Importantly, our findings indicate that Smc5/6 is crucial in limiting those recombination events. We envisage that Smc5/6 promotes replication through natural pausing sites in one or several nonmutually exclusive ways. First, Smc5/6 may modulate fork reversal and/or processing of reversed forks (Xue et al., 2014) formed within topologically stressed regions. Second, Smc5/6 may facilitate resolution of topological constrains by promoting Top3 action on ssDNA regions contained within the positive supercoil in the unreplicated region ahead of the replication fork or on the precatenanes formed by fork rotation. In addition, Smc5/6 may stimulate resolution of the recombination intermediates induced by the topological transitions associated with prolonged pausing.

In conclusion, the present findings indicate Smc5/6 as a keystone regulator of genome integrity via its general roles in regulating DDT intermediate resolution genome-wide, as well as unique roles in modulating recombination at topologically constrained regions such as those happening during prolonged pausing (Figure 6E). We propose that coincident defects in these processes may be a common cause of developmental defects and contribute in the long run to the genome instability characteristic of cancers. Our results also provide leads for future work on understanding the interrelationship between endogenous stress response pathways and genome caretakers in chromosome maintenance processes that affect proliferation.

EXPERIMENTAL PROCEDURES

Yeast Strains and Genetic Screens

The yeast strains used in this study are primarily derivatives of W303, and the relevant genotypes are shown in Table S2.

Growing Conditions, Cell-Cycle Arrests, and Drug Treatments

Strains were grown at 25°C in YPDA medium unless otherwise indicated. G1 and G2 synchronizations were performed using 3–5 μg/ml of α-factor and 15–20 μg/ml of nocodazole, respectively. MMS was used at the concentration of 0.033%, HU at 200 mM, BrdU at 200 μg/ml, EdU at 50 μM, and Tetracycline at 600 μM.

Protein Techniques, ChIP-qPCR, ChIP-on-Chip, and BrdU-IP-on-Chip

BrdU-IP, ChIP-on-chip experiments, and statistical analysis were performed as in Bermejo et al. (2009) and Fachinetti et al. (2010), and ChIP-qPCR as in Urulangodi et al. (2015).

2D Gels, FACS, PFGE, and Molecular Combing

2D gels and in vivo psoralen crosslinking were performed as in Giannattasio et al. (2014), FACS and pulse-field gel electrophoresis as in Branzei et al. (2006), and molecular combing experiments as in Bianco et al. (2012).

ACCESSION NUMBERS

The microarray data are available online under the series number GSE72241 (http://www.ncbi.nlm.nih.gov/geo/query/acc.cgi?acc=GSE72241).

SUPPLEMENTAL INFORMATION

Supplemental Information includes six figures, two tables, and Supplemental Experimental Procedures and can be found with this article at http://dx.doi.org/10.1016/j.molcel.2015.10.023.

AUTHOR CONTRIBUTIONS

D.M. established the reagents; performed all experiments except those in Figures 2B, 2C, and S2; and analyzed the data. A.D., A.L., and P.P. performed and analyzed the combing experiments in Figures 2B, 2C, and S2. D.B. conceived and supervised the project, designed the experiments, analyzed the data, and wrote the paper.

ACKNOWLEDGMENTS

We thank Cogentech facility for hybridization of Affymetrix chips and production of raw data files; C. Lucca for assistance with robot-assisted screens; M. Foiani, I. Psakhye, M. Giannattasio, and T. Abe for critical reading of the manuscript; F. Castellucci for demonstrating ChIP-on-chip experiments; W. Carotenuto for help with microarray data analysis; and B. Szakal for reagents and help with the artistic work. This work was supported by the ERC (Starting Grant 242928), AIRC (IG 14171) and Fondazione Telethon (GGP12160) grants (to D.B.), and Ligue contre le Cancer and ANR (to P.P.). A.L. thanks the Region Languedoc-Roussillon (Programme Chercheur d'Avenir) and ARC for support. A.D. was supported by fellowships from the French Ministry of Research and Fondation Recherche Médicale. D.M. was partially supported by a FIRC fellowship.

Received: September 11, 2015
Revised: October 2, 2015
Accepted: October 9, 2015
Published: November 19, 2015

REFERENCES

Ashton, T.M., Mankouri, H.W., Heidenblut, A., McHugh, P.J., and Hickson, I.D. (2011). Pathways for Holliday junction processing during homologous recombination in Saccharomyces cerevisiae. Mol. Cell. Biol. *31*, 1921–1933.

Azvolinsky, A., Giresi, P.G., Lieb, J.D., and Zakian, V.A. (2009). Highly transcribed RNA polymerase II genes are impediments to replication fork progression in Saccharomyces cerevisiae. Mol. Cell *34*, 722–734.

Bermejo, R., Capra, T., Gonzalez-Huici, V., Fachinetti, D., Cocito, A., Natoli, G., Katou, Y., Mori, H., Kurokawa, K., Shirahige, K., and Foiani, M. (2009). Genome-organizing factors Top2 and Hmo1 prevent chromosome fragility at sites of S phase transcription. Cell *138*, 870–884.

Bermúdez-López, M., Ceschia, A., de Piccoli, G., Colomina, N., Pasero, P., Aragón, L., and Torres-Rosell, J. (2010). The Smc5/6 complex is required for dissolution of DNA-mediated sister chromatid linkages. Nucleic Acids Res. *38*, 6502–6512.

Bermúdez-López, M., Pociño-Merino, I., Sánchez, H., Bueno, A., Guasch, C., Almedawar, S., Bru-Virgili, S., Garí, E., Wyman, C., Reverter, D., et al. (2015). ATPase-dependent control of the Mms21 SUMO ligase during DNA repair. PLoS Biol. *13*, e1002089.

Bianco, J.N., Poli, J., Saksouk, J., Bacal, J., Silva, M.J., Yoshida, K., Lin, Y.L., Tourrière, H., Lengronne, A., and Pasero, P. (2012). Analysis of DNA replication profiles in budding yeast and mammalian cells using DNA combing. Methods *57*, 149–157.

Bicknell, L.S., Bongers, E.M., Leitch, A., Brown, S., Schoots, J., Harley, M.E., Aftimos, S., Al-Aama, J.Y., Bober, M., Brown, P.A., et al. (2011). Mutations in the pre-replication complex cause Meier-Gorlin syndrome. Nat. Genet. *43*, 356–359.

Branzei, D., and Foiani, M. (2008). Regulation of DNA repair throughout the cell cycle. Nat. Rev. Mol. Cell Biol. *9*, 297–308.

Branzei, D., Sollier, J., Liberi, G., Zhao, X., Maeda, D., Seki, M., Enomoto, T., Ohta, K., and Foiani, M. (2006). Ubc9- and mms21-mediated sumoylation counteracts recombinogenic events at damaged replication forks. Cell *127*, 509–522.

Branzei, D., Vanoli, F., and Foiani, M. (2008). SUMOylation regulates Rad18-mediated template switch. Nature *456*, 915–920.

Bustard, D.E., Menolfi, D., Jeppsson, K., Ball, L.G., Dewey, S.C., Shirahige, K., Sjögren, C., Branzei, D., and Cobb, J.A. (2012). During replication stress, non-SMC element 5 (NSE5) is required for Smc5/6 protein complex functionality at stalled forks. J. Biol. Chem. *287*, 11374–11383.

Calzada, A., Hodgson, B., Kanemaki, M., Bueno, A., and Labib, K. (2005). Molecular anatomy and regulation of a stable replisome at a paused eukaryotic DNA replication fork. Genes Dev. *19*, 1905–1919.

Casper, A.M., Nghiem, P., Arlt, M.F., and Glover, T.W. (2002). ATR regulates fragile site stability. Cell *111*, 779–789.

Cha, R.S., and Kleckner, N. (2002). ATR homolog Mec1 promotes fork progression, thus averting breaks in replication slow zones. Science *297*, 602–606.

Choi, K., Szakal, B., Chen, Y.H., Branzei, D., and Zhao, X. (2010). The Smc5/6 complex and Esc2 influence multiple replication-associated recombination processes in Saccharomyces cerevisiae. Mol. Biol. Cell *21*, 2306–2314.

Deshpande, A.M., and Newlon, C.S. (1996). DNA replication fork pause sites dependent on transcription. Science *272*, 1030–1033.

Dewar, J.M., Budzowska, M., and Walter, J.C. (2015). The mechanism of DNA replication termination in vertebrates. Nature *525*, 345–350.

Di Rienzi, S.C., Collingwood, D., Raghuraman, M.K., and Brewer, B.J. (2009). Fragile genomic sites are associated with origins of replication. Genome Biol. Evol. *1*, 350–363.

El Achkar, E., Gerbault-Seureau, M., Muleris, M., Dutrillaux, B., and Debatisse, M. (2005). Premature condensation induces breaks at the interface of early and late replicating chromosome bands bearing common fragile sites. Proc. Natl. Acad. Sci. USA *102*, 18069–18074.

Fachinetti, D., Bermejo, R., Cocito, A., Minardi, S., Katou, Y., Kanoh, Y., Shirahige, K., Azvolinsky, A., Zakian, V.A., and Foiani, M. (2010). Replication termination at eukaryotic chromosomes is mediated by Top2 and occurs at genomic loci containing pausing elements. Mol. Cell *39*, 595–605.

Feng, W., Di Rienzi, S.C., Raghuraman, M.K., and Brewer, B.J. (2011). Replication stress-induced chromosome breakage is correlated with replication fork progression and is preceded by single-stranded DNA formation. G3 (Bethesda) *1*, 327–335.

Fricke, W.M., and Brill, S.J. (2003). Slx1-Slx4 is a second structure-specific endonuclease functionally redundant with Sgs1-Top3. Genes Dev. *17*, 1768–1778.

Giannattasio, M., Zwicky, K., Follonier, C., Foiani, M., Lopes, M., and Branzei, D. (2014). Visualization of recombination-mediated damage bypass by template switching. Nat. Struct. Mol. Biol. *21*, 884–892.

Gritenaite, D., Princz, L.N., Szakal, B., Bantele, S.C., Wendeler, L., Schilbach, S., Habermann, B.H., Matos, J., Lisby, M., Branzei, D., and Pfander, B. (2014). A cell cycle-regulated Slx4-Dpb11 complex promotes the resolution of DNA repair intermediates linked to stalled replication. Genes Dev. *28*, 1604–1619.

Hodgson, B., Calzada, A., and Labib, K. (2007). Mrc1 and Tof1 regulate DNA replication forks in different ways during normal S phase. Mol. Biol. Cell *18*, 3894–3902.

Hombauer, H., Srivatsan, A., Putnam, C.D., and Kolodner, R.D. (2011). Mismatch repair, but not heteroduplex rejection, is temporally coupled to DNA replication. Science *334*, 1713–1716.

Ivessa, A.S., Lenzmeier, B.A., Bessler, J.B., Goudsouzian, L.K., Schnakenberg, S.L., and Zakian, V.A. (2003). The Saccharomyces cerevisiae helicase Rrm3p facilitates replication past nonhistone protein-DNA complexes. Mol. Cell *12*, 1525–1536.

Jackson, L.P., Reed, S.I., and Haase, S.B. (2006). Distinct mechanisms control the stability of the related S-phase cyclins Clb5 and Clb6. Mol. Cell. Biol. *26*, 2456–2466.

Jeppsson, K., Carlborg, K.K., Nakato, R., Berta, D.G., Lilienthal, I., Kanno, T., Lindqvist, A., Brink, M.C., Dantuma, N.P., Katou, Y., et al. (2014a). The chromosomal association of the Smc5/6 complex depends on cohesion and predicts the level of sister chromatid entanglement. PLoS Genet. *10*, e1004680.

Jeppsson, K., Kanno, T., Shirahige, K., and Sjögren, C. (2014b). The maintenance of chromosome structure: positioning and functioning of SMC complexes. Nat. Rev. Mol. Cell Biol. *15*, 601–614.

Karras, G.I., and Jentsch, S. (2010). The RAD6 DNA damage tolerance pathway operates uncoupled from the replication fork and is functional beyond S phase. Cell *141*, 255–267.

Karras, G.I., Fumasoni, M., Sienski, G., Vanoli, F., Branzei, D., and Jentsch, S. (2013). Noncanonical role of the 9-1-1 clamp in the error-free DNA damage tolerance pathway. Mol. Cell *49*, 536–546.

Kegel, A., Betts-Lindroos, H., Kanno, T., Jeppsson, K., Ström, L., Katou, Y., Itoh, T., Shirahige, K., and Sjögren, C. (2011). Chromosome length influences replication-induced topological stress. Nature *471*, 392–396.

Klingseisen, A., and Jackson, A.P. (2011). Mechanisms and pathways of growth failure in primordial dwarfism. Genes Dev. *25*, 2011–2024.

Lehmann, A.R. (2005). The role of SMC proteins in the responses to DNA damage. DNA Repair (Amst.) *4*, 309–314.

Lindroos, H.B., Ström, L., Itoh, T., Katou, Y., Shirahige, K., and Sjögren, C. (2006). Chromosomal association of the Smc5/6 complex reveals that it functions in differently regulated pathways. Mol. Cell *22*, 755–767.

Matos, J., Blanco, M.G., Maslen, S., Skehel, J.M., and West, S.C. (2011). Regulatory control of the resolution of DNA recombination intermediates during meiosis and mitosis. Cell *147*, 158–172.

Mohanty, B.K., Bairwa, N.K., and Bastia, D. (2006). The Tof1p-Csm3p protein complex counteracts the Rrm3p helicase to control replication termination of Saccharomyces cerevisiae. Proc. Natl. Acad. Sci. USA *103*, 897–902.

Murray, J.M., and Carr, A.M. (2008). Smc5/6: a link between DNA repair and unidirectional replication? Nat. Rev. Mol. Cell Biol. *9*, 177–182.

O'Driscoll, M., Ruiz-Perez, V.L., Woods, C.G., Jeggo, P.A., and Goodship, J.A. (2003). A splicing mutation affecting expression of ataxia-telangiectasia and Rad3-related protein (ATR) results in Seckel syndrome. Nat. Genet. *33*, 497–501.

Payne, F., Colnaghi, R., Rocha, N., Seth, A., Harris, J., Carpenter, G., Bottomley, W.E., Wheeler, E., Wong, S., Saudek, V., et al. (2014). Hypomorphism in human NSMCE2 linked to primordial dwarfism and insulin resistance. J. Clin. Invest. *124*, 4028–4038.

Saunus, J.M., Quinn, M.C., Patch, A.M., Pearson, J.V., Bailey, P.J., Nones, K., McCart Reed, A.E., Miller, D., Wilson, P.J., Al-Ejeh, F., et al. (2015). Integrated genomic and transcriptomic analysis of human brain metastases identifies alterations of potential clinical significance. J. Pathol. *237*, 363–378.

Schalbetter, S.A., Mansoubi, S., Chambers, A.L., Downs, J.A., and Baxter, J. (2015). Fork rotation and DNA precatenation are restricted during DNA replication to prevent chromosomal instability. Proc. Natl. Acad. Sci. USA *112*, E4565–E4570.

Sollier, J., Driscoll, R., Castellucci, F., Foiani, M., Jackson, S.P., and Branzei, D. (2009). The Saccharomyces cerevisiae Esc2 and Smc5-6 proteins promote sister chromatid junction-mediated intra-S repair. Mol. Biol. Cell *20*, 1671–1682.

Song, W., Dominska, M., Greenwell, P.W., and Petes, T.D. (2014). Genome-wide high-resolution mapping of chromosome fragile sites in Saccharomyces cerevisiae. Proc. Natl. Acad. Sci. USA *111*, E2210–E2218.

Szakal, B., and Branzei, D. (2013). Premature Cdk1/Cdc5/Mus81 pathway activation induces aberrant replication and deleterious crossover. EMBO J. *32*, 1155–1167.

Szilard, R.K., Jacques, P.E., Laramée, L., Cheng, B., Galicia, S., Bataille, A.R., Yeung, M., Mendez, M., Bergeron, M., Robert, F., and Durocher, D. (2010). Systematic identification of fragile sites via genome-wide location analysis of gamma-H2AX. Nat. Struct. Mol. Biol. *17*, 299–305.

Tittel-Elmer, M., Lengronne, A., Davidson, M.B., Bacal, J., François, P., Hohl, M., Petrini, J.H., Pasero, P., and Cobb, J.A. (2012). Cohesin association to replication sites depends on rad50 and promotes fork restart. Mol. Cell *48*, 98–108.

Torres-Rosell, J., Machín, F., Farmer, S., Jarmuz, A., Eydmann, T., Dalgaard, J.Z., and Aragón, L. (2005). SMC5 and SMC6 genes are required for the segregation of repetitive chromosome regions. Nat. Cell Biol. *7*, 412–419.

Torres-Rosell, J., De Piccoli, G., Cordon-Preciado, V., Farmer, S., Jarmuz, A., Machin, F., Pasero, P., Lisby, M., Haber, J.E., and Aragón, L. (2007). Anaphase onset before complete DNA replication with intact checkpoint responses. Science *315*, 1411–1415.

Tourrière, H., and Pasero, P. (2007). Maintenance of fork integrity at damaged DNA and natural pause sites. DNA Repair (Amst.) *6*, 900–913.

Tourrière, H., Versini, G., Cordón-Preciado, V., Alabert, C., and Pasero, P. (2005). Mrc1 and Tof1 promote replication fork progression and recovery independently of Rad53. Mol. Cell *19*, 699–706.

Urulangodi, M., Sebesta, M., Menolfi, D., Szakal, B., Sollier, J., Sisakova, A., Krejci, L., and Branzei, D. (2015). Local regulation of the Srs2 helicase by the SUMO-like domain protein Esc2 promotes recombination at sites of stalled replication. Genes Dev. *29*, 2067–2080.

Xue, X., Choi, K., Bonner, J., Chiba, T., Kwon, Y., Xu, Y., Sanchez, H., Wyman, C., Niu, H., Zhao, X., and Sung, P. (2014). Restriction of replication fork regression activities by a conserved SMC complex. Mol. Cell *56*, 436–445.

Yunis, J.J., Soreng, A.L., and Bowe, A.E. (1987). Fragile sites are targets of diverse mutagens and carcinogens. Oncogene *1*, 59–69.

Zhao, X., and Blobel, G. (2005). A SUMO ligase is part of a nuclear multiprotein complex that affects DNA repair and chromosomal organization. Proc. Natl. Acad. Sci. USA *102*, 4777–4782.

Zimmermann, C., Chymkowitch, P., Eldholm, V., Putnam, C.D., Lindvall, J.M., Omerzu, M., Bjørås, M., Kolodner, R.D., and Enserink, J.M. (2011). A chemical-genetic screen to unravel the genetic network of CDC28/CDK1 links ubiquitin and Rad6-Bre1 to cell cycle progression. Proc. Natl. Acad. Sci. USA *108*, 18748–18753.

Molecular Cell, Volume *60*

Supplemental Information

Essential Roles of the Smc5/6 Complex in Replication through Natural Pausing Sites and Endogenous DNA Damage Tolerance

Demis Menolfi, Axel Delamarre, Armelle Lengronne, Philippe Pasero, and Dana Branzei

SUPPLEMENTAL INFORMATION

Supplementary Figures and Legends

Figure S1, Related to Figure 1.

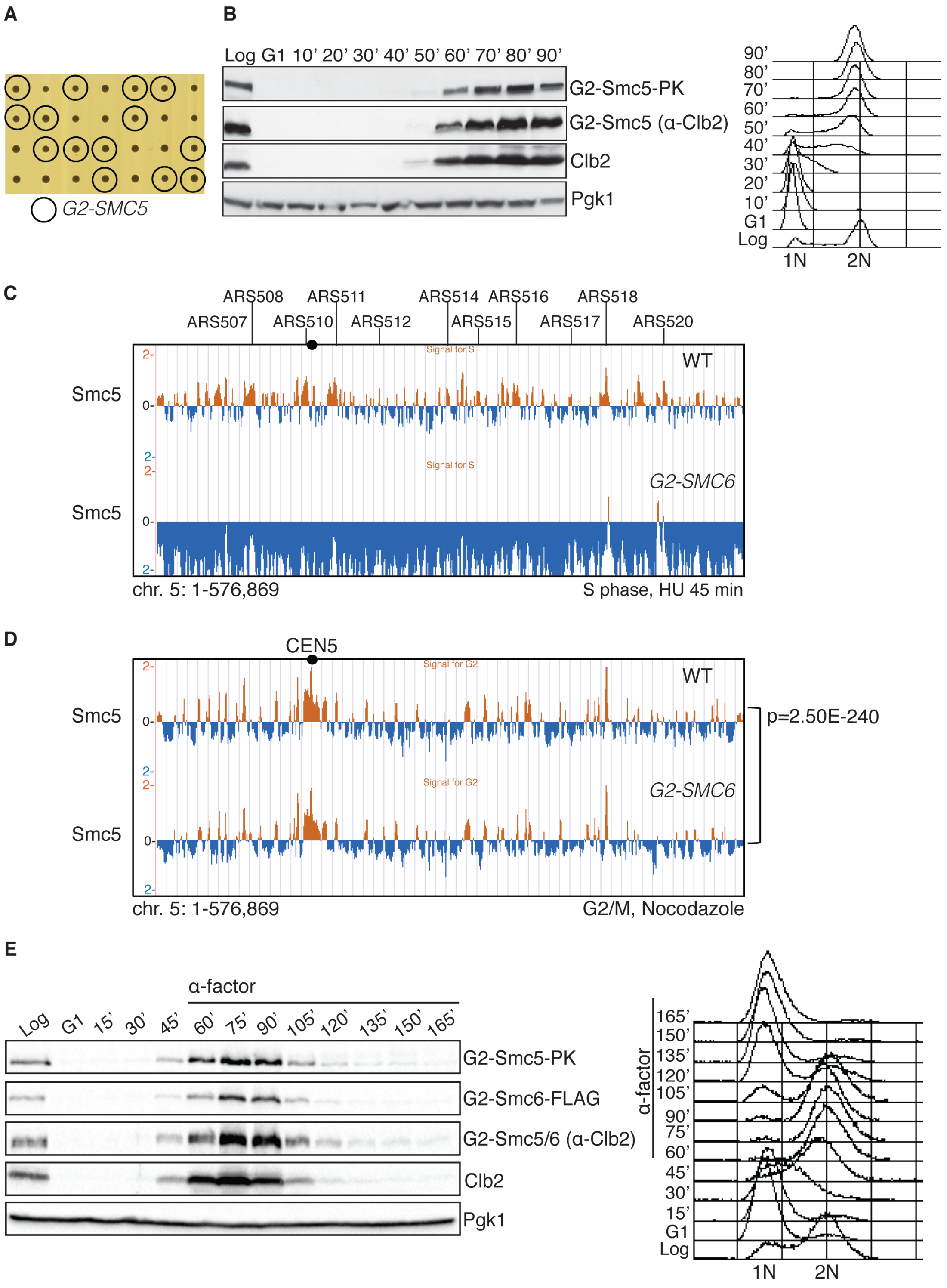

Figure S1. Restriction of individual Smc5/6 subunits to G2/M does not affect cell viability and chromatin association of the complex in G2/M. (A) Heterozygous *SMC5/G2-SMC5* cells were sporulated. *G2-SMC5* haploid cells, isolated by tetrad dissection, were characterized by normal fitness. (B) *G2-SMC5* expression occurred concurrently with the one of *CLB2* in G2/M and was not observed in S phase. FACS profile is also shown. (C) ChIP-on-chip profile of Smc5-PK in WT and *G2-SMC6* cells released from G1 arrest in media containing HU. Chromosome 5 is shown as example, with early origins of replication annotated. (D) ChIP-on-chip profile of Smc5-PK in WT and *G2-SMC6* cells in G2/M. Chromosome 5 is shown as example. The indicated p-value relates to the genome-wide overlap between the Smc5-PK clusters. (E) G2-Smc5/6 is correctly degraded in anaphase. *G2-SMC5 G2-SMC6* cells were synchronized in G1, released in normal medium, and after 60 min, α-factor was added again in order to arrest cells in the following G1. Samples for Western blot and FACS analysis were collected every 15 min.

Figure S2, Related to Figure 2.

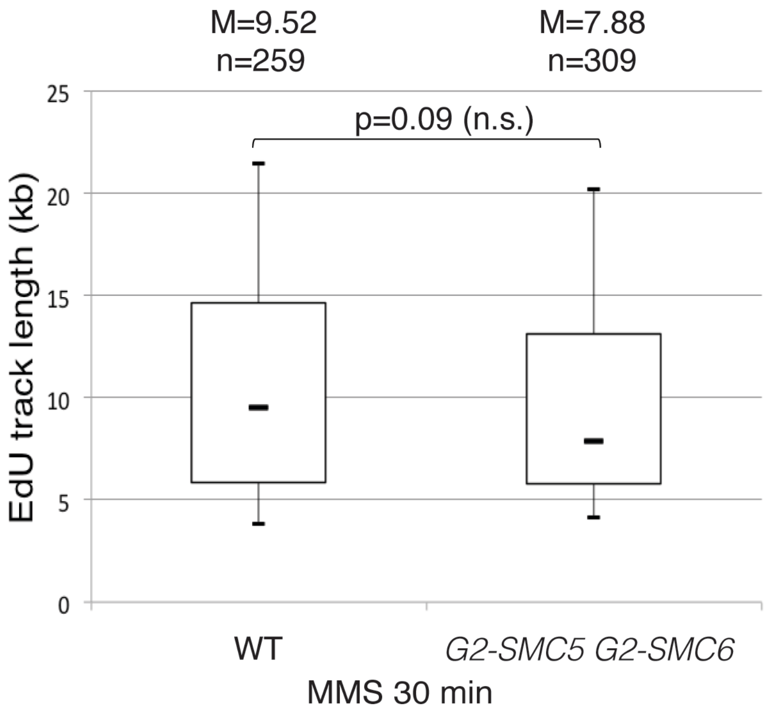

Figure S2. Restriction of Smc5/6 to G2/M does not affect fork progression following MMS treatment. WT and *G2-SMC5 G2-SMC6* cells were synchronized in G1 and released in the presence of EdU and MMS for 30 min, when samples were collected for molecular combing analysis. Box plot of the EdU-track length distribution. The median value of the length (M) and the number of the EdU tracks counted (n) are indicated for each strain. The p-value, calculated with a Mann-Whitney test, indicates that the difference observed between WT and the double mutant is not significant (n.s.).

Figure S3, Related to Figure 3.

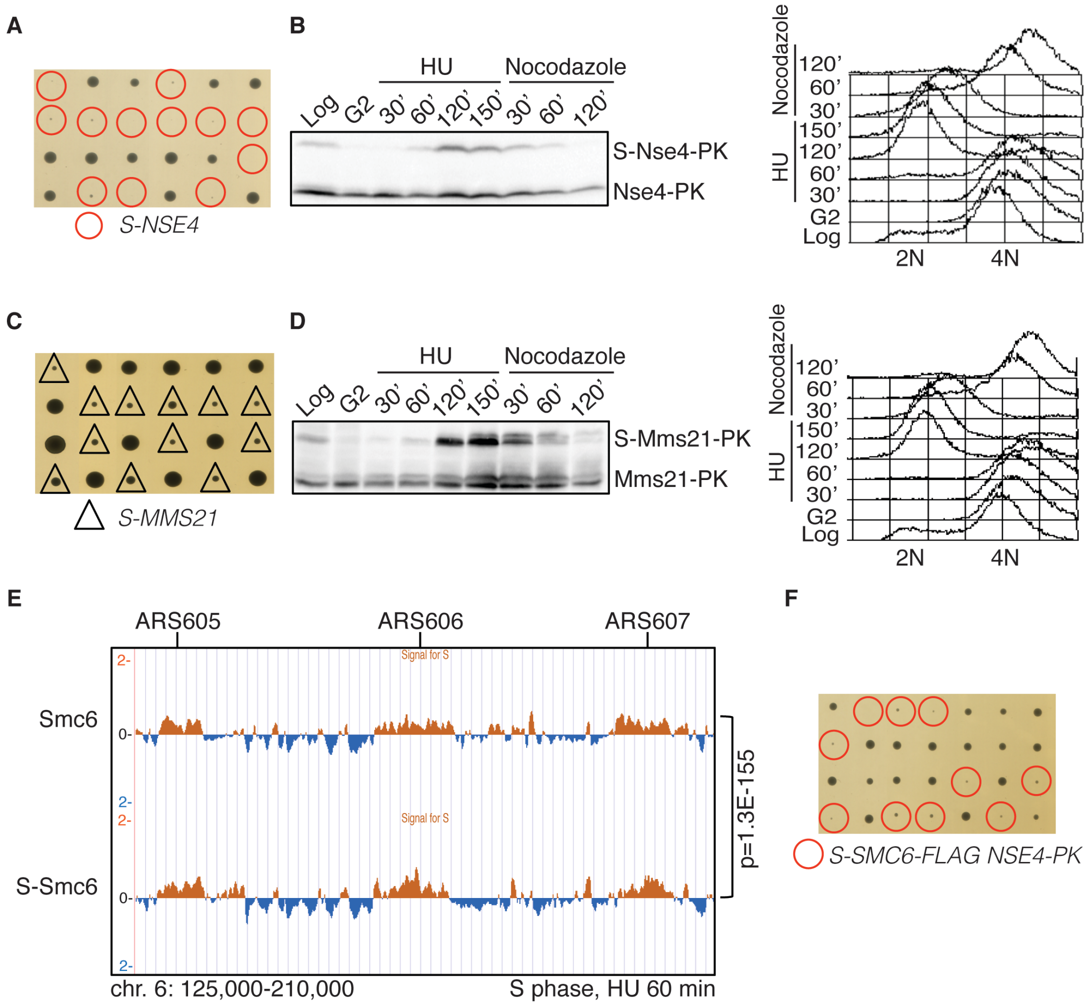

Figure S3. Restriction of Smc5/6 to S phase negatively impacts on cell viability. (A) Heterozygous *NSE4/S-NSE4* cells were sporulated and haploid cells were separated by tetrad dissection. Haploid cells corresponding to *S-NSE4* were not viable. (B) Expression of *NSE4-PK* versus *S-NSE4-PK* alleles in the heterozygous diploid. Asynchronous cells were arrested in G2 with nocodazole, released in media containing HU for 150 min, and then released in media with nocodazole for additional 120 min. Western blot anti-PK and FACS are shown. (C) *S-MMS21* cells obtained by tetrad dissection from heterozygous *MMS21/S-MMS21* cells are slow growing. (D) Expression of *MMS21-PK* versus *S-MMS21-PK* alleles in the heterozygous diploid as described in (B). (E) ChIP-on-chip of Smc6-Flag and S-Smc6-Flag from cells synchronously released from G1 arrest in the presence of HU for 60 min. A snapshot of chromosome 6 is shown with early *ARS* (*ARS605*, *ARS606*, *ARS607*) and genome-

wide p-value of the overlap between the considered protein clusters is indicated. (F) *S-SMC6-FLAG NSE4-PK* double mutants are not viable.

Figure S4, Related to Figure 4.

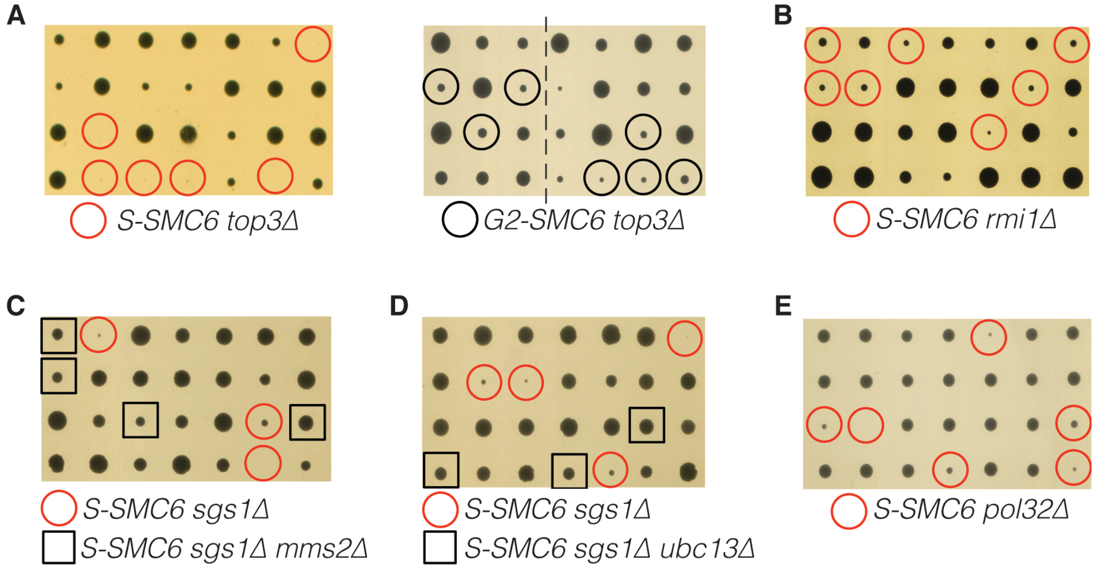

Figure S4. *S-SMC6* viability relies on multiple recombination intermediate resolvases. (A) *S-SMC6*, but not *G2-SMC6*, is synthetic lethal with *top3Δ*. The line indicates elimination of superfluous lanes from the tetrad dissection plate image. (B) *S-SMC6* is synthetic sick with *rmi1Δ*. (C, D) Individual deletions of *MMS2* and *UBC13* rescue the synthetic lethality of *S-SMC6 sgs1Δ*. (E) *S-SMC6* is synthetic lethal/sick with *pol32Δ*.

Figure S5, Related to Figure 5.

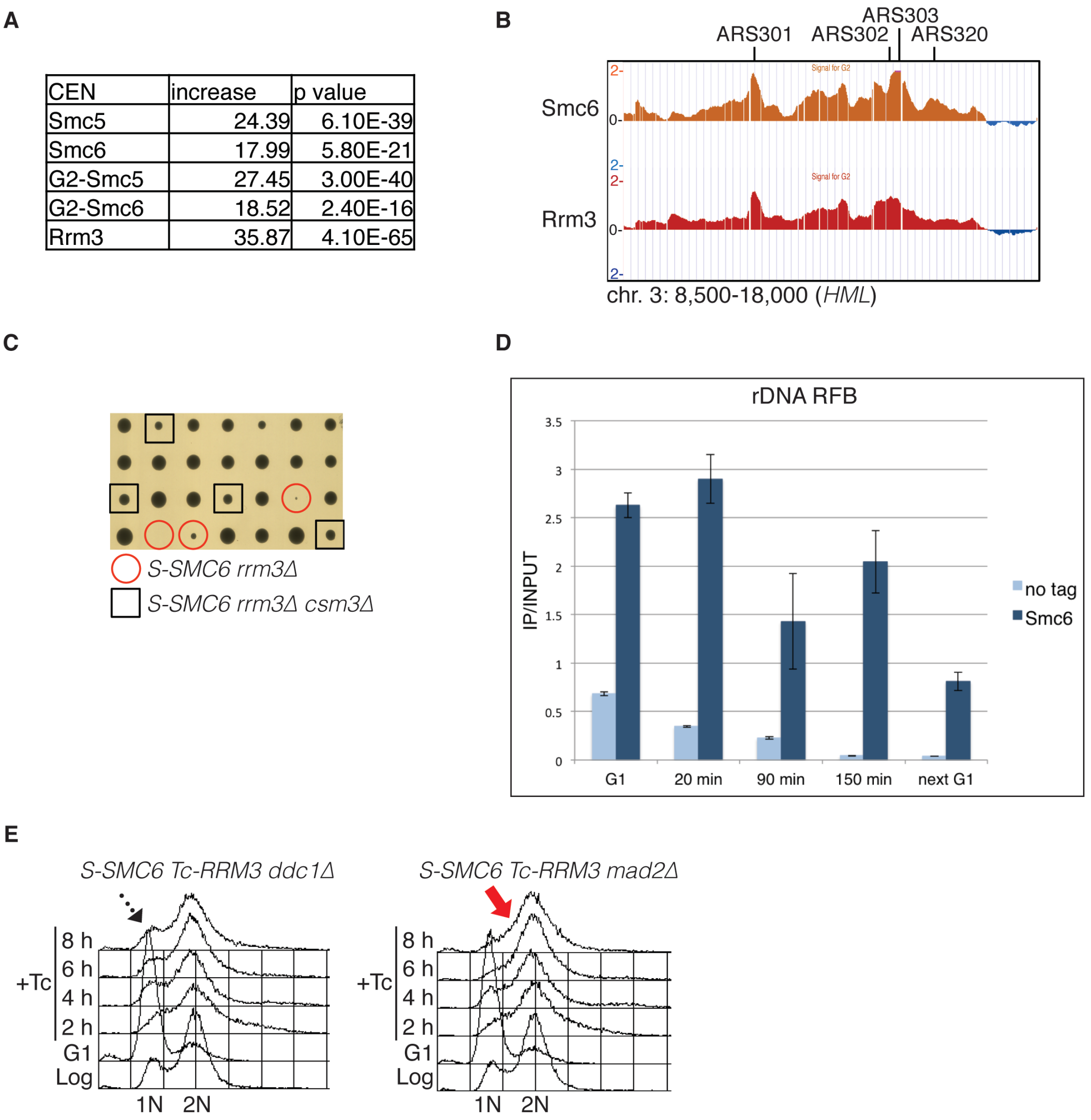

CEN	increase	p value
Smc5	24.39	6.10E-39
Smc6	17.99	5.80E-21
G2-Smc5	27.45	3.00E-40
G2-Smc6	18.52	2.40E-16
Rrm3	35.87	4.10E-65

Figure S5. Smc5/6 localizes and functions at natural pausing elements. (A) Smc5, Smc6, G2-Smc5, G2-Smc6 and Rrm3 are significantly enriched at CENs. The table reports the fold increases of each protein at CENs, calculated versus the values corresponding to random binding, as well as the p-values of the enrichment significance. (B) Smc6 and Rrm3 are enriched at the mating type locus *HML*, known to be a pausing element in budding yeast (Wang et al., 2001). (C) Deletion of *CSM3* rescues the lethality of *S-SMC6 rrm3Δ*. (D) ChIP-qPCR-monitored binding affinity of Smc6-FLAG at rDNA RFB as in Figure 5G. (E) *S-SMC6 Tc-RRM3 ddc1Δ* and *S-SMC6 Tc-RRM3 mad2Δ* cells were synchronized in G1 and released in the presence

of tetracycline (Tc) for 8 hours. Samples for FACS analysis were collected every 2 hours as in Figure 5H.

Figure S6, Related to Figure 6.

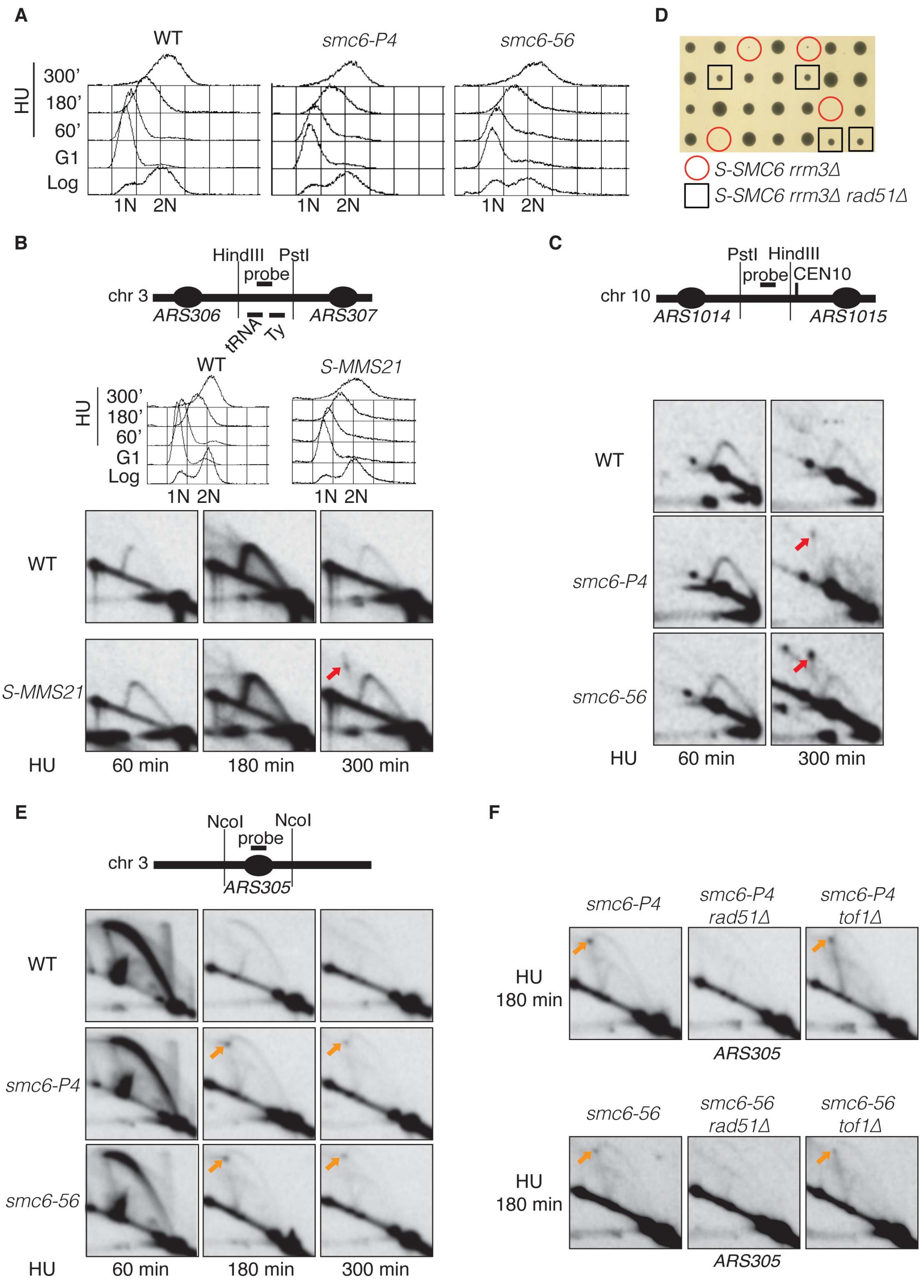

Figure S6. Different functions of Smc5/6 at early replication origins versus regions containing pausing elements. (A) FACS analysis of the 2D gel

electrophoresis experiment in Figure 6C, S6C and S6E (see below). (B) Genomic DNA from WT and *S-MMS21* cells was analyzed for replication intermediates at a pausing site on chromosome 3 as described in Figure 6C. (C) The 2D samples analyzed in Figure 6C were analyzed for another pausing site proximal to the centromere of chromosome 10. (D) Deletion of *RAD51* rescues the lethality of *S-SMC6 rrm3Δ*. (E) WT, *smc6-P4* and *smc6-56* were synchronized in G1 and released in HU at 30°C as described in Figure 6C. The same genomic DNA was digested in parallel with NcoI and analyzed for *ARS305*. (F) Genomic DNA samples isolated from strains of the indicated genotypes after release from G1 arrest in media containing HU were digested with NcoI and analyzed for *ARS305*.

Table S1, Related to Figure 5.

TERs (71)	Smc6-FLAG	Smc5-PK	Rrm3-FLAG
101	+	+	+
102	-	-	+
103	+	+	+
201	+	+	+
202	+	+	+
203	+	+	-
301	-	-	+
302	+	+	+
303	-	-	+
304	-	-	+
305	+	-	+
401	-	-	-
402	+	+	+
403	+	+	+
404	+	+	+
501	+	+	+
502	-	+	+
503	+	+	+
504	+	-	-
505	+	+	+
506	+	+	+
507	-	+	+
508	-	-	+
601	+	-	-
602	+	+	+
603	+	+	+
701	+	+	+
702	+	+	+
703	+	+	+
704	+	+	+
705	+	+	+
801	+	+	+
802	+	+	+
901	-	-	+
902	+	+	+
903	-	+	-
1001	+	+	+
1002	+	+	+
1003	+	+	+
1004	+	+	+
1005	+	+	+
1006	-	-	-
1101	+	+	-
1102	+	+	+
1103	+	+	+
1201	-	-	+
1202	+	+	+
1203	-	-	+
1204	+	+	+
1205	+	+	-
1301	+	+	+
1302	+	+	+
1303	+	+	+

1304	+	+	+
1401	-	+	-
1402	+	+	+
1403	+	+	+
1501	+	+	-
1502	+	+	+
1503	+	+	-
1504	+	+	+
1505	+	+	+
1506	-	-	-
1601	+	+	-
1602	+	+	+
1603	+	+	+
1604	+	+	+
1605	+	+	+
1606	+	+	+
1607	+	+	+
1608	+	+	+
	56	57	58
	79%	80%	81.70%

Table S1. Binding of Smc5/6 and Rrm3 to TER sites. The presence of significant clusters of binding for the protein analyzed by ChIP-on-chip is indicated by "+", lack of it with "-". The TERs are as defined in (Fachinetti et al., 2010) and the ones highlighted in blue were identified as fragile sites in (Song et al., 2014).

Table S2, Related to Figures 1-6. List of *Saccharomyces cerevisiae* strains used in this study.

Strain	Genotype	Source
**FY1363**	Mata *ade2-1 can1-100 his3-11,-15 leu2-3,112 trp1-1 ura3-1 RAD5+* (W303)	Lab collection
**FY0090**	Mata *his3-delta200 leu2-3, 112 lys2-801 trp1-1 (am) ura3-52* (DF5)	Lab collection
**HY1465**	W303 Mata *sgs1Δ::HIS3MX6*	Lab collection
**HY1473**	W303 Mata *mus81Δ::KANMX4*	Lab collection
**HY1545**	W303 Mata *sgs1Δ::HIS3MX6 rad5Δ::HPHMX4*	Lab collection
**HY1547**	W303 Mata *sgs1Δ::HIS3MX6 ubc13Δ::HPHMX4*	Lab collection
**HY1549**	W303 Mata *sgs1Δ::HIS3MX6 mms2Δ::HPHMX4*	Lab collection
**HY2721**	DF5 Matα *pol32Δ::klTRP1*	Lab collection
**HY2736**	W303 Mata *G2::NATNT2-SMC6*	This study
**HY2806**	W303 Mata *SMC6-6HIS-3FLAG::KANMX4*	This study
**HY3156**	W303 Mata *SMC5-9PK::HIS3MX6*	This study
**HY3158**	W303 Mata *G2::NATNT2-SMC6 SMC5-9PK::HIS3MX6*	This study
**HY3159**	W303 Mata *G2::NATNT2-SMC5-9PK::HIS3MX6*	This study
**HY3167**	W303 Mata *S::NATNT2-SMC6*	This study
**HY3168**	W303 Matα *S::NATNT2-SMC6*	This study
**HY3170**	W303 Mata *S::NATNT2-SMC6-6HIS-3FLAG::KANMX4*	This study
**HY3172**	W303 Mata *G2::NATNT2-SMC6-6HIS-3FLAG::KANMX4 G2::NATNT2-SMC5-9PK::HIS3MX6*	This study
**HY3386**	W303 Mata *S::NATNT2-SMC6-6HIS-3FLAG::KANMX4 rad51Δ::LEU2*	This study
**HY3447**	W303 Mata *SMC6-6HIS-3FLAG::KANMX4 SMC5-9PK::HIS3MX6*	This study
**HY3611**	W303 Mata *rmi1Δ::KANMX4*	Lab collection
**HY3807**	W303 Mata *TOP3-6HIS-3FLAG::KANMX4*	This study
**HY3840**	W303 Mata *ubc13Δ::HPHMX4*	Lab collection
**HY3841**	W303 Matα *mms2Δ::HPHMX4*	Lab collection
**HY3973**	W303 Matα *S::NATNT2-SMC6-6HIS-3FLAG::KANMX4*	This study
**HY4421**	W303 Mata *S::NATNT2-SMC6 tof1Δ::HIS3MX6*	This study
**HY4422**	W303 Mata *S::NATNT2-SMC6 csm3Δ::HPHMX4*	This study
**HY4425**	W303 Mata *S::NATNT2-SMC6 fob1Δ::HIS3MX6*	This study
**HY4896**	W303 Mata *ura3::URA3/GPD-TK(7X) G2::NATNT2-SMC6-6HIS-3FLAG::KANMX4 G2::NATNT2-SMC5-9PK-HIS3MX6*	This study
**HY4898**	W303 Mata *S::NATNT2-MMS21*	This study
**HY4904**	W303 Matα *S::NATNT2-SMC6 mms2Δ::HPHMX4*	This study
**HY4905**	W303 Mata *S::NATNT2-SMC6 ubc13Δ::HPHMX4*	This study
**HY4906**	W303 Mata/α *SMC5-9PK::HIS3MX6/S::NATNT2-SMC5-9PK::TRP1*	This study
**HY4909**	DF5 Mata *S::NATNT2-SMC6*	This study
**HY4915**	W303 Mata *S::NATNT2-SMC6 rad5Δ::HPHMX4*	This study
**HY4916**	W303 Mata *RRM3-10FLAG::KANMX4*	This study
**HY5163**	W303 Mata/α *MMS21-9PK::HIS3MX6/S::NATNT2-MMS21-9PK::TRP1*	This study

**HY5274**	W303 Mata *S::NATNT2-SMC6-6HIS-3FLAG::KANMX4 pAHD1-tc3-3HA-RRM3 (NAT)*	This study
**HY5276**	DF5 Mata *S::NATNT2-SMC6 pol32Δ::klTRP1*	This study
**HY5324**	W303 Mata/α *SMC5-6HIS-3FLAG::KANMX4/S::NATNT2-SMC5-9PK::TRP1*	This study
**HY5396**	W303 Mata *S::NATNT2-SMC6-6HIS-3FLAG::KANMX4 pAHD1-tc3-3HA-RRM3 (NAT) rad9Δ::HIS3MX6*	This study
**HY5398**	W303 Mata *S::NATNT2-SMC6-6HIS-3FLAG::KANMX4 pAHD1-tc3-3HA-RRM3 (NAT) ddc1Δ::HIS3MX6*	This study
**HY5400**	W303 Mata *S::NATNT2-SMC6-6HIS-3FLAG::KANMX4 pAHD1-tc3-3HA-RRM3 (NAT) mad2Δ:HPHMX4*	This study
**HY5433**	W303 Mata/α *NSE4-9PK::HIS3MX6/S::NATNT2-NSE4-9PK::TRP1*	This study
**HY5845**	W303 Mata *S::NATNT2-SMC6-6HIS-3FLAG::KANMX4 rad51Δ::LEU2 sgs1Δ::HIS3MX6*	This study
**HY5846**	W303 Mata *S::NATNT2-SMC6 rad5Δ::HPHMX4 sgs1Δ::HIS3MX6*	This study
**HY5847**	W303 Mata *S::NATNT2-SMC6 ubc13Δ::HPHMX4 sgs1Δ::HIS3MX6*	This study
**HY5848**	W303 Mata *S::NATNT2-SMC6 mms2Δ::HPHMX4 sgs1Δ::HIS3MX6*	This study
**HY5849**	W303 Mata *S::NATNT2-SMC6 mus81Δ::KANMX4*	This study
**HY5850**	W303 Mata *S::NATNT2-SMC6 rmi1Δ::KANMX4*	This study
**HY5851**	W303 Mata *G2::NATNT2-SMC6 top3Δ::KANMX4*	This study
**HY5853**	W303 Mata *S::NATNT2-SMC6 rrm3Δ::HIS3MX6 rad51Δ::LEU2*	This study
**HY5854**	W303 Mata *S::NATNT2-SMC6 rrm3Δ::HIS3MX6 tof1Δ::KANMX4*	This study
**HY5855**	W303 Mata *S::NATNT2-SMC6 rrm3Δ::HIS3MX6 csm3Δ::HPHMX4*	This study
**HY5856**	W303 Mata *S::NATNT2-SMC6 rrm3Δ::HIS3MX6 fob1Δ::HIS3MX6*	This study
**HY5861**	W303 Mata *G2::NATNT2-SMC6 sgs1Δ::HIS3MX6*	This study
**HY5862**	W303 Mata *G2::NATNT2-SMC6 rrm3Δ::HIS3MX6*	This study
**HY5890**	W303 Mata *smc6-56-13MYC::KANMX4 tof1Δ::HPHMX4*	This study
**HY5892**	W303 Mata *smc6-P4-13MYC::KANMX4 tof1Δ::HPHMX4*	This study
**HY5940**	W303 Mata *NSE4-9PK::HIS3MX6*	This study
**FY1002**	W303 Matα *rad51Δ::LEU2*	Lab collection
**FY1110**	W303 Mata *ura3::URA3/GPD-TK(7X)*	Foiani lab
**FY1267**	W303 Mata *smc6-56-13MYC::HIS3MX6 rad51Δ::LEU2*	Zhao lab
**FY1332**	W303 Mata *smc6-P4-13MYC::KANMX4*	Zhao lab
**FY1432**	W303 Mata *smc6-56-13MYC::KANMX4*	Zhao lab
**FY1535**	W303 Mata *smc6-P4-13MYC::KANMX4 rad51Δ::LEU2*	Zhao lab
**FY1765**	W303 Matα *rrm3Δ::HIS3MX6*	Foiani lab
**FY1766**	W303 Matα *top3Δ::KANMX4*	Foiani lab
**FY1856**	W303 Mata *ura3::URA3/GPD-TK(7X) [pRS415-hent1-LEU2]*	This study
**FY1857**	W303 Mata *ura3::URA3/GPD-TK(7X) G2::NATNT2-SMC6-6HIS-3FLAG::KANMX4 G2::NATNT2-SMC5-9PK-HIS3MX6 [pRS415-hent1-LEU2]*	This study

SUPPLEMENTAL EXPERIMENTAL PROCEDURES

Chromatin immunoprecipitation (ChIP)-on-chip and statistical analysis

Chromatin immunoprecipitation was carried out as previously described (Bermejo et al., 2009a; Bermejo et al., 2009b). Briefly, cells were collected at the indicated experimental conditions and crosslinked with 1% formaldehyde. Cells were washed twice with ice-cold TBS 1X, suspended in lysis buffer supplemented with 1 mM PMSF and 1X antiproteolytic cocktail (Complete protease inhibitor tablets, Roche) and lysed with FastPrep-24 (MP Biomedicals). Chromatin was shared to a size of 300-500 bp by sonication. IP reactions, with anti-FLAG (F1804, SIGMA) or anti-PK SV5-Pk1 (AbD Serotech) conjugated to Dynabeads Protein A (Invitrogen), were allowed to proceed overnight at 4°C. After washing and eluting the ChIP fractions from beads, crosslinks were reversed at 65°C overnight for both SUP and IP. After Proteinase K (Roche) treatment, DNA was extracted twice by phenol/chlorophorm/isoamylalcol. Following precipitation with ethanol and RNase A (SIGMA) treatment, DNA was purified using QIAquick PCR purification kit (QIAGEN). For ChIP-on-chip, DNA was amplified using WGA kit (SIGMA) following manufacturer's instructions. 4 µg of DNA from SUP and IP samples were hybridized to GeneChip *S. cerevisiae* Tiling 1.0R Array (Affimetrix) as described (Bermejo et al., 2009b). Evaluation of the significance of protein cluster distributions within the different genomic areas and protein-binding correlations was performed by confrontation to the model of the null hypothesis distribution generated by a Monte Carlo-like simulation as previously described (Bermejo et al., 2009a). The significance of the overlap between proteins clusters was evaluated as in (Bermejo et al., 2009a).

The microarray data are available online at the following link: http://www.ncbi.nlm.nih.gov/geo/query/acc.cgi?acc=GSE72241.

ChIP-qPCR

ChIP-qPCR was performed using QuantiFast (SYBR Green PCR kit, QIAGEN) according to the manufacturer's recommendations and each real-time was performed at least in triplicate using a Roche LightCycler 480 system. The results were analyzed with absolute quantification/2nd derivative maximum (Roche LightCycler 480) and the 2(-

ΔC(t)) method as previously described (Livak and Schmittgen, 2001). Error bars represent standard deviations.

Antibodies

Monoclonal Anti-FLAG M2 (SIGMA, F1804), monoclonal anti-PK (AbD Serotech, SV5-Pk1), monoclonal anti-Pgk1 22C5 antibody (Invitrogen, A6457), polyclonal anti-Clb2 (Santa Cruz Biotechnology, y-180) were used. The BrdU-IP-on-chip analysis was carried out employing the anti-BrdU antibody MBL M1-11-3.

Pulse-Field Gel Electrophoresis (PFGE)

Genomic DNA samples were processed in low melting point agarose plugs (Branzei et al., 2006). For each time point analyzed agarose plugs containing 5 X 10^7 cells were incubated for 1 h at 37°C in 1 M sorbitol, 0.06 M EDTA, 0.1 M sodium citrate, 1 mg/ml Zymolyase and 0.2% β-mercaptoethanol. The plugs were then incubated in 0.5 M EDTA, 1% sarkosyl, 1mg/ml RNase A for 1 h at 37°C. Then, 2 mg/ml of Proteinase K was added and left overnight at 37°C. After extensive washing in buffer containing 10 mM Tris pH 7.5 and 50 mM EDTA, the agarose plugs were inserted in 0.9% agarose TBE 0.5X gel. Pulse-Field Gel Electrophoresis was performed for 10 h at 190V with 60s pulses, followed by 10h with 90 s pulses, in TBE 0.5X at 10°C. The gel was stained for 30 min with 0.5 µg/ml ethidium bromide and scanned on FluorImager (Molecular Dynamics). After PFGE, DNA was depurinated in 0.25 N HCl for 10 min, denaturated in 0.5M NaOH and 1.5M NaCl for 20 min, neutralized in 1M ammonium acetate and 0.02M NaOH for 20 min, and transferred to a nitrocellulose membrane (Whatman Protran) by Southern Blotting. Hybridization was done with probes recognizing pausing sites on chromosomes 3 and 6.

2D Gel Electrophoresis

Cells were synchronized in G1 with α-factor at 25°C and released in media containing HU 0.2 M at 30°C. Samples were collected at the indicated time points and incubated with Sodium Azide 1% for 30 min on ice. *In vivo* psoralen crosslinking and DNA extraction with CTAB were performed as in (Giannattasio et al., 2014). DNA samples were digested with *Hind*III and *Pst*I and analyzed with probes recognizing natural pausing sites on chromosomes 3 and 10. In parallel, DNA samples were digested with NcoI and analyzed with a probe against *ARS305*.

Supplemental References

Bermejo, R., Capra, T., Gonzalez-Huici, V., Fachinetti, D., Cocito, A., Natoli, G., Katou, Y., Mori, H., Kurokawa, K., Shirahige, K., *et al.* (2009a). Genome-organizing factors Top2 and Hmo1 prevent chromosome fragility at sites of S phase transcription. Cell *138*, 870-884.

Bermejo, R., Katou, Y.M., Shirahige, K., and Foiani, M. (2009b). ChIP-on-chip analysis of DNA topoisomerases. Methods Mol. Biol. *582*, 103-118.

Branzei, D., Sollier, J., Liberi, G., Zhao, X., Maeda, D., Seki, M., Enomoto, T., Ohta, K., and Foiani, M. (2006). Ubc9- and mms21-mediated sumoylation counteracts recombinogenic events at damaged replication forks. Cell *127*, 509-522.

Fachinetti, D., Bermejo, R., Cocito, A., Minardi, S., Katou, Y., Kanoh, Y., Shirahige, K., Azvolinsky, A., Zakian, V.A., and Foiani, M. (2010). Replication termination at eukaryotic chromosomes is mediated by Top2 and occurs at genomic loci containing pausing elements. Mol. Cell *39*, 595-605.

Giannattasio, M., Zwicky, K., Follonier, C., Foiani, M., Lopes, M., and Branzei, D. (2014). Visualization of recombination-mediated damage bypass by template switching. Nat. Struct. Mol. Biol. *21*, 884-892.

Livak, K.J., and Schmittgen, T.D. (2001). Analysis of relative gene expression data using real-time quantitative PCR and the 2(-Delta Delta C(T)) Method. Methods *25*, 402-408.

Song, W., Dominska, M., Greenwell, P.W., and Petes, T.D. (2014). Genome-wide high-resolution mapping of chromosome fragile sites in Saccharomyces cerevisiae. Proc. Natl. Acad. Sci. USA *111*, E2210-2218.

Wang, Y., Vujcic, M., and Kowalski, D. (2001). DNA replication forks pause at silent origins near the HML locus in budding yeast. Mol. Cell. Biol. *21*, 4938-4948.
